# Supplementary material for: The importance and availability of adjustments to improve access for autistic adults who need mental and physical healthcare: findings from UK surveys
Source: BMJ Open. 2021 Mar 18;11(3):e043336. doi: 10.1136/bmjopen-2020-043336 (PMC7978247; doi:10.1136/bmjopen-2020-043336)
Supplement: Supplementary data [file bmjopen-2020-043336supp001.pdf]

Supplementary Table 1: Demographic information for the mental health and physical health samples and comparison with non-responders

	Mental Health (PAT-A)		Physical Health (IHOAP)	
**Variable**	**Responders**	**Non-responders**	**Responders**	**Non-responders**
**Total N**	537	545	407	484
**Gender N (%)**				
Male	234 (43.6)	273 (50.1)	167 (41.0)	238 (49.1)
Female	281 (52.3)	254 (46.6)	227 (55.8)	236 (48.8)
Other/rather not say	22 (4.1)	18 (3.3)	13 (3.2)	10 (2.1)
**Age: Mean (SD) [Range]**	41.3 (13.8) [17.0-77.0]	37.6 (12.9) [17.0-84.0]	44.4 (13.4) [18.0-79.0]	41.0 (13.3) [18.0-90.0]
**Ethnicity**				
White	501 (93.3)	496 (91.0)	374 (91.9)	437 (90.3)
Asian	3 (0.6)	8 (1.5)	1 (0.2)	6 (1.2)
Black	2 (0.4)	3 (0.6)	3 (0.7)	2 (0.4)
Mixed	10 (1.9)	10 (1.8)	8 (2.0)	11 (2.3)
Other/rather not say	8 (1.5)	4 (0.7)	7 (1.7)	6 (1.2)
Not reported	13 (2.4)	24 (4.4)	14 (3.4)	26 (5.4)
**Highest education**				
Postgraduate degree	94 (17.5)	67 (12.3)	72 (17.7)	69 (14.3)
Bachelor's degree	135 (25.1)	122 (22.4)	97 (23.8)	97 (20.0)
Diploma of higher education	34 (6.3)	43 (7.9)	28 (6.9)	52 (10.7)
Certificate of higher education	18 (3.4)	27 (5.0)	17 (4.2)	19 (3.9)
A-level	88 (16.4)	115 (21.1)	76 (18.7)	93 (19.2)
GCSE	108 (20.1)	86 (15.8)	71 (17.4)	85 (17.6)
Basic skills	20 (3.7)	27 (5.0)	10 (2.5)	24 (5.0)
No formal qualifications	32 (6.0)	34 (6.2)	30 (7.4)	36 (7.4)
Other	8 (1.5)	24 (4.4)	6 (1.5)	9 (1.9)
**Employment status N (%)**				
Employed without support[a]	213 (39.7)	204 (37.4)	154 (37.8)	163 (33.7)
Employed with support	9 (1.7)	8 (1.5)	5 (1.2)	10 (2.1)
Volunteer	49 (9.1)	55 (10.1)	38 (9.3)	10 (2.1)
Unemployed	170 (31.7)	188 (34.5)	137 (33.7)	202 (41.7)
Retired	30 (5.6)	20 (3.7)	25 (6.1)	24 (5.0)
Other	58 (10.8)	57 (10.5)	38 (9.3)	62 (12.8)
No response	8 (1.5)	13 (2.4)	10 (2.5)	13 (2.7)
**ASD diagnosis N (%)**				
Formal diagnosis	443 (82.5)[d]	467 (85.7)	341 (83.8)[e]	415 (85.7)
Suspected/unsure/awaiting diagnosis	94 (17.5)[d]	78 (14.3)	66 (16.2)[e]	69 (14.3)
**Age at diagnosis: Mean (SD) [Range]**	35.8 (15.8) [3.0-73.0]	30.1 (15.5) [2.0-69.0]	38.7 (14.8) [2.0-68.0]	34.4 (15.8) [2.0-86.0]
**SRS severity N (%)**				
Normal	20 (4.2)	27 (7.2)	9 (2.5)	19 (5.7)
Mild	45 (9.4)	35 (9.3)	35 (9.7)	31 (9.2)
Moderate	172 (35.8)	112 (29.8)	115 (31.9)	97 (28.9)
Severe	244 (50.7)	202 (53.7)	201 (55.8)	189 (56.3)
**SRS Mean (SD)**	110.5 (25.6)	112.9 (29.2)	115.1 (25.8)	115.6 (28.2)
**Support received**[c] **N (%)**				
Home	152 (15.5)	162 (13.6)	115 (15.2)	142 (17.4)
Employment	50 (5.1)	78 (6.5)	36 (4.8)	53 (6.5)
Health	110 (11.2)	153 (12.8)	95 (12.6)	130 (16.0)
Finance	129 (13.2)	154 (12.9)	99 (13.1)	137 (16.8)
Social	75 (7.7)	115 (9.6)	57 (7.5)	88 (10.8)
Lifelong learning	67 (6.9)	75 (6.3)	47 (6.2)	60 (7.4)
Community	78 (8.0)	110 (9.2)	68 (9.0)	79 (9.7)
Organisation	73 (7.5)	110 (9.2)	55 (7.3)	93 (11.4)

Do not receive support	244 (24.9)	236 (19.8)	183 (24.2)	32 (3.9)
**Mental health condition N (%)**				
Diagnosis	-	-	351 (86.2)	414 (85.5)
No diagnosis	-	-	56 (13.8)	70 (14.5)
**Physical health condition N (%)**				
Diagnosis	397 (73.9)	402 (73.8)	-	-
No diagnosis	140 (26.1)	143 (26.2)	-	-
**Response type**				
Electronic	415 (77.3%	446 (81.8)	305 (74.9)	396 (81.8)
Paper	122 (22.7%)	99 (18.2)	102 (25.1)	88 (18.2)

Statistical comparison of key demographic factors within and between samples and between responders and non-responders

Tests of difference between responders and non-responders on key demographic variables

Significant differences are denoted with *

Age:
Mental health sample: $t(859)= 1.655$, $p=0.949$
Physical health sample: $t(668)= 3.369$, $p=0.001$*

Gender:
Mental health sample: M,F,O,R genders Mental health sample vs Physical health sample $\chi^2(3)=2.049$, $p=0.562$; males and females only: $\chi^2(1)=0.349$, $p=0.555$
Physical health sample: M,F,O,R genders Mental health sample vs Physical health sample $\chi^2(3)=4.481$, $p=0.214$; males and females only: $\chi^2(1)=1.007$, $p=0.316$

Autism Characteristics (SRS-2 total score):
Mental health sample: $t(641.5)= -1.135$, $p=0.257$
Physical health sample: $t(496)= -0.198$, $p=0.843$

Tests of difference in autism characteristics between those reporting an autism spectrum diagnosis and those who suspect that they are autistic or are awaiting diagnosis within subsamples

Mental health sample:
112.5 (autism diagnosis), 112.4 (no autism diagnosis). $t(479)= -0.002$, $p=0.998$

Physical health sample:
115.7 (autism diagnosis), 112.3 (no autism diagnosis). $t(358)= -0.893$, $p=0.372$)
