# Supplementary material for: The importance and availability of adjustments to improve access for autistic adults who need mental and physical healthcare: findings from UK surveys
Source: BMJ Open. 2021 Mar 18;11(3):e043336. doi: 10.1136/bmjopen-2020-043336 (PMC7978247; doi:10.1136/bmjopen-2020-043336)
Supplement: Supplementary data [file bmjopen-2020-043336supp002.pdf]

Supplementary Table 2: Factor loadings for the three-factor solution for importance of adjustments to mental health services and four-factor solution for the importance of adjustments to physical health services

Adjustment	Factor	Mental Health			Physical Health			
	Factor Factor Code	**F1 SE**	**F2 SC**	**F3 CKC**	**F1 SE**	**F2 SC**	**F3 CKC**	**F4 F**
Change the sensory environment in the building that the appointment will take place in		**0.799**	0.064	0.006	**0.925**	-0.178	0.120	0.015
Locations (e.g. waiting rooms) with small numbers of people		**0.787**	-0.001	0.081	**0.621**	0.258	-0.212	0.245
Locations with low noise levels		**1.065**	-0.186	-0.054	**0.905**	-0.094	-0.051	0.064
Locations with low light levels		**0.718**	0.099	-0.055	**0.874**	0.109	0.076	-0.230
Changing the length of appointments to suit you		-0.100	**0.551**	0.212	-0.098	0.222	0.376	0.284
Offering appointments online or via apps		-0.164	**0.765**	-0.222	-0.002	**0.502**	0.009	-0.123
Changing how often you are asked to attend appointments		-0.062	**0.685**	-0.038	-0.145	**0.829**	0.063	-0.094
Give information to the clinician pre-appointment so that they can prepare		-0.159	**0.417**	0.378	-0.070	0.119	**0.671**	0.041
Provide support in relation to attending appointments (e.g. managing fears or uncertainties which might make attending difficult)		0.117	**0.511**	0.184	0.115	0.294	0.087	0.378
Appropriate distractions provided whilst waiting to be seen at appointment (e.g. tablet with headphones)		0.215	**0.680**	-0.162	0.292	**0.422**	0.001	0.039
Therapists who understand autism		0.047	-0.458	**0.970**	0.274	-0.044	0.282	0.202
Opportunity after the appointment to ask questions about conclusions		-0.244	-0.162	**0.838**	-0.137	0.010	**0.836**	-0.042
Appointments at an easily identified and accessible location		0.063	-0.057	**0.625**	0.026	-0.041	0.043	**0.668**
Appointments with an easily identified and familiar clinician		0.124	-0.110	**0.685**	-0.036	-0.253	0.026	**1.044**
Having a health summary document which can be shared with clinicians (e.g. hospital passport)		-0.019	0.168	**0.556**	0.143	-0.163	**0.861**	-0.188
A clinician who uses an approach which is informed by what you have said that you prefer (e.g. formal or informal)		0.035	0.016	**0.624**	0.066	-0.135	**0.736**	0.103
Identifying reasons that make it difficult to see a clinician or attend an appointment		0.096	0.193	**0.474**	0.053	0.230	**0.495**	0.041
Short waiting times to be seen when you attend appointments		0.268	0.169	0.295	0.002	**0.410**	-0.028	**0.421**

Key: SS = Sensory Environment, SC = Clinical and Service Context, CKC = Clinician Knowledge and Communication, F = Familiarity

Factor loadings that exceed the ≥ 0.4 loading criteria are denoted in bold
