# Supplementary material for: The importance and availability of adjustments to improve access for autistic adults who need mental and physical healthcare: findings from UK surveys
Source: BMJ Open. 2021 Mar 18;11(3):e043336. doi: 10.1136/bmjopen-2020-043336 (PMC7978247; doi:10.1136/bmjopen-2020-043336)
Supplement: Supplementary data [file bmjopen-2020-043336supp003.pdf]

Supplementary Table 3: Inter-factors correlation matrices

	Mental Health			Physical Health			
	**F1**	**F2**	**F3**	**F1**	**F2**	**F3**	**F4**
**F1**	1.000			1.000			
**F2**	0.688	1.000		0.722	1.000		
**F3**	0.732	0.785	1.000	0.595	0.727	1.000	
**F4**				0.694	0.705	0.736	1.000
