# Supplementary material for: The importance and availability of adjustments to improve access for autistic adults who need mental and physical healthcare: findings from UK surveys
Source: BMJ Open. 2021 Mar 18;11(3):e043336. doi: 10.1136/bmjopen-2020-043336 (PMC7978247; doi:10.1136/bmjopen-2020-043336)
Supplement: Supplementary data [file bmjopen-2020-043336supp004.pdf]

Supplementary Table 4: Model fit indices

Model fit statistic	Mental Health	Physical Health
**Root Mean Square Error of Approximation (RMSEA)**	0.032	0.032
**Non-Normed Fit Index (NNFI)**	0.994	0.995
**Comparative Fit Index (CFI)**	0.996	0.997
**Goodness of Fit Index (GFI)**	0.993	0.995
**Root Mean Square of Residuals (RMSR)**	0.0384	0.0361

The following cut-off values were used to determine acceptable goodness of fit:

RMSEA < 0.08
NNFI ≥ 0.95
CFI ≥ 0.90
GFI ≥ 0.95
RMSR < 0.08.
