# Supplementary material for: The importance and availability of adjustments to improve access for autistic adults who need mental and physical healthcare: findings from UK surveys
Source: BMJ Open. 2021 Mar 18;11(3):e043336. doi: 10.1136/bmjopen-2020-043336 (PMC7978247; doi:10.1136/bmjopen-2020-043336)
Supplement: Supplementary data [file bmjopen-2020-043336supp005.pdf]

Supplementary Table 5: Response frequencies for the importance of adjustments in both samples

Response option key: 1 = not at all important, 2 = not very important, 3 = neither important or unimportant, 4 = somewhat important, 5 = very important, M = missing response

Adjustment	Mental Health Services Importance Response Frequencies (N)						Physical Health Services Importance Response Frequencies (N)					
	1 Low	2	3	4	5 High	M	1 Low	2	3	4	5 High	M
Clinicians who understand autism	1	0	8	57	468	3	2	4	8	64	322	7
Changing the length of appointments to suit you	19	40	102	181	194	1	12	20	52	135	177	11
Offering appointments online or via apps	89	66	128	120	120	14	65	45	84	73	121	19
Changing how often you are asked to attend appointments	32	33	173	163	130	6	51	42	110	109	69	26
Give information to the clinician pre-appointment so that they can prepare	11	17	34	147	322	6	20	13	43	130	190	11
Opportunity after the appointment to ask questions about conclusions	5	10	31	166	321	4	8	5	27	100	253	14
Appointments at an easily identified and accessible location	5	5	43	104	374	6	6	8	30	70	281	12
Appointments with an easily identified and familiar clinician	8	11	34	112	365	7	9	9	18	78	279	14
Change the sensory environment in the building that the appointment will take place in	32	28	83	178	209	7	23	23	56	123	166	16
Locations (e.g. waiting rooms) with small numbers of people	19	25	55	162	268	8	17	14	39	116	208	13
Locations with low noise levels	10	16	32	149	322	8	11	14	21	104	244	13
Locations with low light levels	43	42	103	153	185	11	20	27	81	121	138	20
Having a health summary document which can be shared with clinicians (e.g. hospital passport)	16	17	73	152	268	11	13	15	69	100	188	22
A clinician who uses an approach which is informed by what you have said that you prefer (e.g. formal or informal)	6	6	31	167	318	9	7	15	53	117	193	22
Identifying reasons that make it difficult to see a clinician or attend an appointment	13	8	70	174	260	12	10	8	48	114	202	25
Short waiting times to be seen when you attend appointments	7	20	44	168	293	5	7	14	34	111	227	14
Provide support in relation to attending appointments (e.g. managing fears or uncertainties which might make attending difficult)	20	21	58	146	282	10	18	14	50	109	198	18
Appropriate distractions provided whilst waiting to be seen at appointment (e.g. tablet with headphones)	62	66	142	121	132	14	51	31	101	93	106	25
