# Supplementary material for: The importance and availability of adjustments to improve access for autistic adults who need mental and physical healthcare: findings from UK surveys
Source: BMJ Open. 2021 Mar 18;11(3):e043336. doi: 10.1136/bmjopen-2020-043336 (PMC7978247; doi:10.1136/bmjopen-2020-043336)
Supplement: Supplementary data [file bmjopen-2020-043336supp006.pdf]

Supplementary Table 6: Response frequencies for the availability of adjustments in both samples

Response option key: 1 = never available, 2 = rarely available, 3 = available about half the time, 4 = available most of the time, 5 = available all the time, DK = I don't know, M = missing response

Adjustment	Mental Health Services Availability Response Frequencies (N)							Physical Health Services Availability Response Frequencies (N)						
	1 Low	2	3	4	5 High	DK	M	1 Low	2	3	4	5 High	DK	M
Therapists who understand autism	81	105	32	27	14	261	17	86	84	30	23	10	146	28
Changing the length of appointments to suit you	102	52	29	32	10	292	20	96	68	25	27	16	134	41
Offering appointments online or via apps	94	32	25	26	18	321	21	76	41	35	36	45	125	49
Changing how often you are asked to attend appointments	50	46	32	37	16	335	21	65	34	21	19	8	192	68
Give information to the clinician pre-appointment so that they can prepare	73	50	31	42	23	301	17	92	56	25	29	21	139	45
Opportunity after the appointment to ask questions about conclusions	50	63	32	73	46	255	18	69	52	43	65	28	113	37
Appointments at an easily identified and accessible location	31	47	62	103	64	209	21	38	35	52	94	73	79	36
Appointments with an easily identified and familiar clinician	37	52	50	90	56	232	20	47	73	81	66	25	79	36
Change the sensory environment in the building that the appointment will take place in	108	63	26	27	13	279	21	114	55	16	14	9	124	45
Locations (e.g. waiting rooms) with small numbers of people	83	63	57	54	26	235	19	131	72	31	26	7	100	40
Locations with low noise levels	72	72	51	47	35	237	23	116	73	32	36	15	98	37
Locations with low light levels	101	60	31	36	19	261	29	129	76	12	23	6	111	50
Having a health summary document which can be shared with clinicians (e.g. hospital passport)	100	34	18	18	22	320	25	117	33	7	13	14	174	49
A clinician who uses an approach which is informed by what you have said that you prefer (e.g. formal or informal)	59	70	44	52	26	265	21	91	45	35	28	16	147	45
Identifying reasons that make it difficult to see a clinician or attend an appointment	72	55	29	37	19	300	25	92	52	23	20	11	152	57
Short waiting times to be seen when you attend appointments	78	72	43	69	25	228	22	102	97	50	29	8	79	42
Provide support in relation to attending appointments (e.g.	95	51	32	35	22	280	22	100	67	24	12	15	136	43

managing fears or uncertainties which might make attending difficult)													
Appropriate distractions provided whilst waiting to be seen at appointment (e.g. tablet with headphones)	145	36	14	21	13	281	27	138	38	10	9	13	137	62
