# Supplementary material for: The importance and availability of adjustments to improve access for autistic adults who need mental and physical healthcare: findings from UK surveys
Source: BMJ Open. 2021 Mar 18;11(3):e043336. doi: 10.1136/bmjopen-2020-043336 (PMC7978247; doi:10.1136/bmjopen-2020-043336)
Supplement: Supplementary data [file bmjopen-2020-043336supp007.pdf]

Supplementary Table 7: McNemar tests showing a comparison of the adjustments that were most important to autistic people in mental and physical health services in a paired sample

Adjustment	N	Mental Health % very important	Physical Health % very important	P value
Clinician who understands autism	217	87.6	80.2	.048
Changing the length of appointments to suit you	217	39.6	46.1	.211
Offering appointments online or via apps	206	23.8	28.6	.332
Changing how often you are asked to attend appointments	204	23.0	20.1	.526
Give information to the clinician pre-appointment so that they can prepare	214	64.5	49.5	.004
Opportunity after the appointment to ask questions about conclusions	214	62.6	64.0	.845
Appointments at an easily identified and accessible location	213	74.6	71.4	.520
Appointments with an easily identified and familiar clinician	211	69.7	72.0	.668
Change the sensory environment in the building that the appointment will take place in	210	40.0	44.8	.378
Locations (e.g. waiting rooms) with small numbers of people	213	50.2	51.2	.925
Locations with low noise levels	212	57.5	58.5	.924
Locations with low light levels	208	31.3	36.1	.373
Having a health summary document which can be shared with clinicians (e.g. hospital passport)	207	51.7	47.8	.497
A clinician who uses an approach which is informed by what you have said that you prefer (e.g. formal or informal)	206	64.6	46.6	**.001**
Identifying reasons that make it difficult to see a clinician or attend an appointment	202	47.0	51.5	.452
Short waiting times to be seen when you attend appointments	213	55.4	56.8	.848
Provide support in relation to attending appointments (e.g. managing fears or uncertainties which might make attending difficult)	208	55.8	50.0	.299
Appropriate distractions provided whilst waiting to be seen at appointment (e.g. tablet with headphones)	203	24.1	27.1	.572

* Significant P values following Simes adapted Bonferroni correction are highlighted in bold
