# Supplementary material for: The importance and availability of adjustments to improve access for autistic adults who need mental and physical healthcare: findings from UK surveys
Source: BMJ Open. 2021 Mar 18;11(3):e043336. doi: 10.1136/bmjopen-2020-043336 (PMC7978247; doi:10.1136/bmjopen-2020-043336)
Supplement: Supplementary data [file bmjopen-2020-043336supp008.pdf]

Supplementary Table 8: McNemar tests showing a comparison of the adjustments that were least available to autistic people in mental and physical health services in a paired sample

Adjustment	N	Mental Health % never available	Physical Health % never available	P value
Clinician who understands autism	65	29.2	40.0	.230
Changing the length of appointments to suit you	54	53.7	40.7	.296
Offering appointments online or via apps	43	46.5	34.9	.424
Changing how often you are asked to attend appointments	21	23.8	38.1	.549
Give information to the clinician pre-appointment so that they can prepare	54	35.2	38.9	.864
Opportunity after the appointment to ask questions about conclusions	72	20.8	25.0	.690
Appointments at an easily identified and accessible location	92	15.2	10.9	.503
Appointments with an easily identified and familiar clinician	82	14.6	17.1	.832
Change the sensory environment in the building that the appointment will take place in	51	60.8	51.0	.383
Locations (e.g. waiting rooms) with small numbers of people	75	32.0	49.3	.060
Locations with low noise levels	71	29.6	33.8	.711
Locations with low light levels	55	50.9	45.5	.701
Having a health summary document which can be shared with clinicians (e.g. hospital passport)	31	64.5	48.4	.302
A clinician who uses an approach which is informed by what you have said that you prefer (e.g. formal or informal)	58	29.5	31.0	.690
Identifying reasons that make it difficult to see a clinician or attend an appointment	43	48.8	46.5	1.000
Short waiting times to be seen when you attend appointments	91	29.7	36.3	.451
Provide support in relation to attending appointments (e.g. managing fears or uncertainties which might make attending difficult)	55	45.5	36.4	.442
Appropriate distractions provided whilst waiting to be seen at appointment (e.g. tablet with headphones)	51	74.5	72.5	1.000
