# Supplementary material for: The importance and availability of adjustments to improve access for autistic adults who need mental and physical healthcare: findings from UK surveys
Source: BMJ Open. 2021 Mar 18;11(3):e043336. doi: 10.1136/bmjopen-2020-043336 (PMC7978247; doi:10.1136/bmjopen-2020-043336)
Supplement: Author's manuscript [file bmjopen-2020-043336.draft_revisions.pdf]

BMJ Open is committed to open peer review. As part of this commitment we make the peer review history of every article we publish publicly available.

When an article is published we post the peer reviewers' comments and the authors' responses online. We also post the versions of the paper that were used during peer review. These are the versions that the peer review comments apply to.

The versions of the paper that follow are the versions that were submitted during the peer review process. They are not the versions of record or the final published versions. They should not be cited or distributed as the published version of this manuscript.

BMJ Open is an open access journal and the full, final, typeset and author-corrected version of record of the manuscript is available on our site with no access controls, subscription charges or pay-per-view fees (http://bmjopen.bmj.com).

If you have any questions on BMJ Open's open peer review process please email info.bmjopen@bmj.com

BMJ Open

The importance and availability of adjustments to improve access for autistic adults who need mental and physical healthcare: findings from a UK survey

Journal:	*BMJ Open*
Manuscript ID	bmjopen-2020-043336
Article Type:	Original research
Date Submitted by the Author:	30-Jul-2020

Complete List of Authors:	Brice, Samuel; Newcastle University Faculty of Medical Sciences, Population Health Sciences Institute<br>Rodgers, Jacqui; Newcastle University Faculty of Medical Sciences, Population Health Sciences Institute<br>Ingham, Barry; Newcastle University Faculty of Medical Sciences, Population Health Sciences Institute; Cumbria Northumberland Tyne and Wear NHS Foundation Trust<br>Mason, David; Newcastle University Faculty of Medical Sciences, Population Health Sciences Institute<br>Wilson, Colin ; Newcastle University Faculty of Medical Sciences, Population Health Sciences Institute<br>Freeston, Mark; Newcastle University Faculty of Medical Sciences, School of Psychology<br>Le Couteur , Ann; Newcastle University Faculty of Medical Sciences, Population Health Sciences Institute<br>Parr, Jeremy; Newcastle University Faculty of Medical Sciences, Population Health Sciences Institute; Cumbria Northumberland Tyne and Wear NHS Foundation Trust

Keywords:	MENTAL HEALTH, Quality in health care < HEALTH SERVICES ADMINISTRATION & MANAGEMENT, Organisational development < HEALTH SERVICES ADMINISTRATION & MANAGEMENT

SCHOLARONE™
Manuscripts

I, the Submitting Author has the right to grant and does grant on behalf of all authors of the Work (as defined in the below author licence), an exclusive licence and/or a non-exclusive licence for contributions from authors who are: i) UK Crown employees; ii) where BMJ has agreed a CC-BY licence shall apply, and/or iii) in accordance with the terms applicable for US Federal Government officers or employees acting as part of their official duties; on a worldwide, perpetual, irrevocable, royalty-free basis to BMJ Publishing Group Ltd ("BMJ") its licensees and where the relevant Journal is co-owned by BMJ to the co-owners of the Journal, to publish the Work in this journal and any other BMJ products and to exploit all rights, as set out in our licence.

The Submitting Author accepts and understands that any supply made under these terms is made by BMJ to the Submitting Author unless you are acting as an employee on behalf of your employer or a postgraduate student of an affiliated institution which is paying any applicable article publishing charge ("APC") for Open Access articles. Where the Submitting Author wishes to make the Work available on an Open Access basis (and intends to pay the relevant APC), the terms of reuse of such Open Access shall be governed by a Creative Commons licence – details of these licences and which Creative Commons licence will apply to this Work are set out in our licence referred to above.

Other than as permitted in any relevant BMJ Author's Self Archiving Policies, I confirm this Work has not been accepted for publication elsewhere, is not being considered for publication elsewhere and does not duplicate material already published. I confirm all authors consent to publication of this Work and authorise the granting of this licence.

For peer review only

The importance and availability of adjustments to improve access for autistic adults who need mental and physical healthcare: findings from a UK survey

Authors

Samuel Brice samuel.brice@ncl.ac.uk

Jacqui Rodgers jacqui.rodgers@ncl.ac.uk

Barry Ingham barry.ingham@cntw.nhs.uk

David Mason david.mason@ncl.ac.uk

Colin Wilson colin.wilson@ncl.ac.uk

Mark Freeston mark.freeston@ncl.ac.uk

Ann Le Couteur a.s.le-couteur@ncl.ac.uk

Jeremy R. Parr jeremy.parr@ncl.ac.uk (corresponding author)

For peer review only - http://bmjopen.bmj.com/site/about/guidelines.xhtml

Abstract

Objectives: To investigate autistic people's views on the importance and availability of adjustments that are designed to promote the accessibility and acceptability of mental and physical healthcare provision. To explore whether specific categories of adjustments can be identified using exploratory factor analysis and to identify any differences in their importance and availability between mental and physical healthcare.

Design: Data was from two studies, both employing a cross-sectional survey design.

Setting: Autistic adults based in the UK who had previously registered on a research cohort completed and returned the survey either online or by post depending on their preferences.

Participants: 537 autistic adults completed a survey about mental health services, 407 completed the physical health survey. Within these samples, 221 participants completed both surveys.

Primary outcome measures: Each study developed a bespoke survey to explore participants' views on mental and physical health services respectively. Both included an identical list of adjustments which participants were asked to rate based on their importance and availability.

Results: Three categories of important adjustments were identified: the sensory environment, clinical and service context, and clinician knowledge and communication. Adjustments across healthcare settings were widely rated as being important yet rarely available. Only one significant difference between the importance of adjustments available through mental and physical health services was identified. This was having access to a clinician who is willing to adapt their approach to the person's preferences ($p = .001 < .0028$).

Conclusions: Autistic people confirm the importance and limited availability of adjustments in current healthcare provision. To address this unmet need, healthcare providers must offer adjustments relating to the three identified factors as part of a personalised approach in order

For peer review only - http://bmjopen.bmj.com/site/about/guidelines.xhtml

to tackle the health inequalities faced by autistic people. Future research should focus on identifying and addressing service-provider barriers to implementation.

Strengths and limitations of this study

- This research matched published research priorities of the autism community and focused directly on the experiences of autistic people
- This study reports on key adjustments to both physical and mental health services
- Our data driven approach enabled us to determine overarching categories of adjustments that could be used to inform both policy and practice in relation to service development
- A notable proportion of participants reported that they did not have sufficient knowledge to comment on the availability of adjustments
- This study did not seek the opinions of health service providers, which may be a prudent next step for future research

For peer review only

For peer review only - http://bmjopen.bmj.com/site/about/guidelines.xhtml

Introduction

Autism Spectrum Disorder[1] is characterised by lifelong persistent difficulties with social communication and the presence of patterns of restrictive and repetitive behaviours or interests including hyper or hyporeactivity to sensory aspects of the environment (1). At least 1.1% of the United Kingdom (UK) population is autistic (2).

Autistic people are more likely to experience mental and physical health conditions (3); this persists across the lifespan (4). Poor health has been shown to predict poorer quality of life (5, 6). Autistic adults are more likely to experience physical health conditions, such as epilepsy, cardiovascular disease and diabetes (7). Anxiety and depression are particularly common, with an estimated lifetime prevalence of around 40% (8, 9). Autistic people are also more likely to experience multiple mental and physical health conditions concurrently (10). Epidemiological studies suggest autistic people are also significantly more likely to face premature mortality, with as many as 12 years less life reported for autistic adults without intellectual disability (11).

Barriers to healthcare access

Autistic adults face a number of significant barriers to health care access and treatment (12, 13). To date, the most frequently reported barriers have been considered within three factors: individual-level (e.g. barriers to effective communication with clinicians, sensory sensitivities), provider-level (e.g. lack of 'autism awareness' amongst clinicians) and system-level (e.g. convoluted referral pathways and lack of availability of specialist services). Recent research indicates that these barriers prevent autistic people from arranging and attending healthcare appointments (14). In addition, other individual factors such as difficulties coping with the

[1] We will use the term 'autism' or 'autistic person' throughout this paper, reflecting a growing consensus that this terminology is preferred by the autism community. This terminology includes previously recognised subcategories of autism, including but not limited to Asperger's Disorder, Autistic Disorder and Pervasive Developmental Disorder.

For peer review only - http://bmjopen.bmj.com/site/about/guidelines.xhtml

potential uncertainty of healthcare situations (15), and pre-existing anxiety (8) may make the experience of attending healthcare appointments especially problematic for autistic people. Further, although the UK National Health Service (NHS) is publicly funded and free at the point of access, in other countries the affordability of healthcare has been shown to be a barrier that is disproportionately more likely to affect autistic people (16). There is also evidence of a lack of training, skills and/or confidence among healthcare professionals in working with and delivering interventions to autistic people (17), which may impact on engagement and retention (12).

Several UK autism community top research priorities focus on aspects of physical and mental healthcare including 'How should service delivery for autistic people be improved and adapted in order to meet their needs?' (18). Many autistic people report dissatisfaction with the current accessibility of healthcare across both physical and mental health settings (19, 20). Several recent UK clinical guidelines have recommended that to reduce barriers to healthcare, providers should adjust their procedures and approach to best meet the individual needs of autistic people, with additional specific guidance for mental health services, to meet the needs of people with intellectual disabilities (21-23). A recent study concluded that "adjustments for communication needs are as necessary for autistic people as ramps are for wheelchair users" (14). Evidence suggests that adjustments to services do facilitate healthcare access for autistic people (13). However, the importance and availability of specific adjustments across healthcare settings is unknown.

In this study, we sought to identify autistic people's experiences regarding the importance and availability of a range of adjustments to mental and physical healthcare that have been suggested as 'good practice' in previous research and clinical guidance.

For peer review only - http://bmjopen.bmj.com/site/about/guidelines.xhtml

Aims

1. To investigate autistic people's views on the importance and availability of adjustments that are designed to promote the accessibility and acceptability of mental and physical healthcare provision.

2. To explore whether specific categories of adjustments can be identified using exploratory factor analysis and to identify any differences in their importance and availability between mental and physical healthcare.

Method

Design

This study reports on data from two UK studies – one focused on anxiety (as an exemplar of a mental health condition) and the other focused on physical health conditions. Both employed a cross-sectional survey design and were undertaken in 2018-2019.

Participants

Participants for both studies were recruited via the Adult Autism Spectrum Cohort – UK (ASC-UK; https://research.ncl.ac.uk/adultautismspectrum/), a longitudinal cohort study of autistic adults. ASC-UK recruits from a wide variety of UK sources including NHS diagnostic/health services, charitable/community organisations and also receives self-referrals. In line with previous research (e.g. 6), these studies included some participants who were awaiting autism assessment or suspected that they were autistic (18% and 16% of mental health and physical health samples respectively).

For peer review only - http://bmjopen.bmj.com/site/about/guidelines.xhtml

Participants completed paper or web-based materials, depending on their preference. Following consent, participants completed a registration questionnaire that included questions on demographics, mental and physical health, education, employment, and aspects of daily living. Participants also complete the Social Responsiveness Scale -2nd Edition (SRS-2, (24)).

The Personalised Anxiety Treatment – Autism (PAT-A) study investigated autistic people's experiences of anxiety and access to mental healthcare, with a particular focus on accessing psychological therapies, the primary treatment choice for anxiety conditions in UK clinical guidance (22). Inclusion criteria were autistic people living in the UK aged 18 years and older. 1113 autistic people aged 18 and over who had previously reported a diagnosed or suspected anxiety condition were invited to participate. 568 (51%) returned the consent form and completed the survey. Thirty-one participants consented and did not respond to any of the questions about adjustments and were not included in analyses, resulting in a final sample of 537.

The Improving the Health of Autistic People study (IHOAP) investigated the physical health of autistic people, and experiences of accessing healthcare for physical health conditions, including impact on daily life, screening services offered and taken up, and factors that made accessing health care difficult. Inclusion criteria were autistic people living in the UK aged 18 years and older. 945 autistic adults were invited to participate; 461 (49%) people consented and completed the survey. 54 participants did not respond to any survey questions about adjustments and were therefore not included in analyses, resulting in a final sample of 407.

For peer review only - http://bmjopen.bmj.com/site/about/guidelines.xhtml

There were 723 unique participants in the two studies, including 221 participants who completed both studies. Demographic information (from ASC-UK) for participants from the PAT-A and IHOAP studies is provided in Table 1.

Procedure

Eligible participants were sent an information sheet (including an 'easyread' version), consent form and survey, either by post or email depending on their contact preferences. Those preferring to be contacted online were sent a unique link based on a participant number and completed the consent form and survey using Qualtrics© (25). Participants who received materials by post completed a written consent form and survey and returned this to the research team in a prepaid envelope. Written informed consent was provided by all participants. Participants who had not responded after two weeks were sent a single reminder letter. Participation was not incentivised. All procedures were approved by the NHS Health Research Authority and a Research Ethics Committee (both Wales REC 5, Approval reference numbers: PAT-A: 18/WA/0014; IHOAP: 18/WA/0191).

Patient and public involvement (PPI)

This research is directly matched to published research priorities of the autism community (18). The ASC-UK cohort study involved autistic people at the design stage as well as in the design and delivery of ongoing associated projects. Prior to commencing both studies, PPI focus groups were convened with community stakeholders to discuss and agree the methods and materials, including survey questions. An autistic person and parent of an autistic person are co-investigators on this research. Author CW is autistic and was involved with the analysis of data and preparation of this manuscript. Our dissemination plan includes consideration of the

For peer review only - http://bmjopen.bmj.com/site/about/guidelines.xhtml

most effective ways to share these findings with autistic people, including presentations and newsletter updates.

Measures

Both samples completed the same survey about adjustments to services. Other data were also collected as part of the PAT-A and IHOAP studies and will be reported separately (manuscripts in process). The survey questionnaires included a list of adjustments previously reported to improve access, inclusivity and appropriateness of health services, together with an option for open text responses to record any other adjustments. The list was based on published evidence (13), clinical experience and the Reasonable Adjustments Flag (NHS Digital, https://digital.nhs.uk/services/reasonable-adjustment-flag; (26)). The list of adjustments can be seen in Tables 2 and 3.

Participants were asked to rate the importance of each adjustment on a five-point Likert scale from 1) not at all important; 2) not very important; 3) neither important nor unimportant; 4) somewhat important and 5) very important. Participants also rated how available they thought each adjustment was using a five-point scale 1) never available, 2) rarely available, 3) available about half the time, 4) available most of the time, 5) available all of the time. For availability, a 6th option 'I don't know' was provided to enable those who did not feel that they had enough experience of that particular adjustment to report this, enabling the research team to discriminate between lack of experience and a missing response. Any additional adjustments identified by participants in open text response boxes were recorded.

For peer review only - http://bmjopen.bmj.com/site/about/guidelines.xhtml

The Social Responsiveness Scale – 2nd Edition (SRS-2; (24))

The SRS-2 is a widely used standardised self-report questionnaire used to characterise autism 'severity'. The SRS-2 has demonstrated excellent internal consistency and the predictive validity of the adult form has been reported to have a specificity level of .60 and a sensitivity of .86 (27). The full-scale score was used to make comparisons between those who self-reported a diagnosis of autism and those who suspected they had autism or were awaiting assessment; and between responders and non-responders (further detail in Table 1 and supplementary Table 1).

Analysis

Exploratory factor analysis (EFA) was conducted identify whether the adjustments could be empirically grouped in a meaningful way, using data from both datasets (FACTOR software version 10.8.04 (28). The polychoric correlation matrix was subject to unweighted least squares extraction with Promin rotation. Bias correlated bootstrap resampling was undertaken using 5000 bootstrap samples and model fit was formally assessed. Participants with one or more missing responses on any of the importance of adjustment ratings were deleted prior to EFA, resulting in the removal of 51 participants from the mental health dataset (final N = 486), and 78 participants from the physical health dataset (N = 329). Participant-to-item ratios of 27:1 and 18:1 were deemed more than adequate; the Kaiser-Meyer-Olkin (KMO) indicated that the sampling adequacy was 'very good' in both datasets. See supplemental materials for full details.

Quantitative data were analysed descriptively. Open text responses about adjustments were analysed using framework analysis (29). One researcher (JR) coded open text responses against the adjustment categories indicated in EFA. Discrepancies were discussed and agreed with

For peer review only - http://bmjopen.bmj.com/site/about/guidelines.xhtml

another researcher (SB). Additional adjustments that did not fit within these categories were recorded under the category ‘other’.

Service-level differences in adjustments that were deemed most important to autistic people and least available were explored using McNemar’s test to compare nominal data in the paired sample of 221 participants who completed the PAT-A and IHOAP surveys. Variables were first dichotomized to separate the proportion who selected that an adjustment was ‘very important’ compared with all other levels of importance and ‘never available’ compared with all other levels of availability. McNemar’s comparisons were computed for each of the 18 core adjustments based on the dichotomised importance and availability ratings. Simes’ modified Bonferroni correction (30) was used to minimise risk of type I error.

Results

For both the mental health and physical health samples, there was no significant difference between the SRS score of those who reported an autism spectrum diagnosis, and those who suspected they were autistic or who were awaiting autism assessment (Independent t-tests: mental health: 112.5 vs 112.4 respectively, $t(479)= -0.002$, $p=0.998$; physical health: 115.7 vs 112.3 respectively, $t(358)= -0.893$, $p=0.372$).

Comparisons between the study participants and those who did not respond can be seen in Supplementary Table 1. In both mental and physical health study samples, there were no significant differences between responders and non-responders on gender or SRS score. In the physical health, but not the mental health study, responders were significantly older than non-responders ($t(668)= 3.369$, $p=0.001$).

For peer review only - http://bmjopen.bmj.com/site/about/guidelines.xhtml

Considering mental and physical health study participants, key demographic characteristics are shown in Table 1. Both mental and physical health samples contained a broadly equal split of genders, with slightly more females in both. There was a large overlap in health conditions between the samples (74% of the mental health sample reporting a physical health condition and 86% of the physical health sample reporting a mental health condition). Comparisons were made between key demographic characteristics of the two samples (participants who completed both studies were not included to avoid double coding). There was no significant difference in gender ($\chi^2(3)=2.493$, $p=0.477$) or SRS-2 score ($t(443)= -1.814$, $p=0.70$) between samples. The physical health sample were significantly older than the mental health sample ($t(500)= -4.526$, $p<0.001$).

[INSERT TABLE 1]

Service use

73.4% of the participants who completed the anxiety survey had been prescribed medication for anxiety and 76.5% had been referred for psychological therapies for anxiety. Of those who were referred for psychological therapies, 80% completed treatment, 16% stopped attending before treatment was considered complete and 4% did not attend. Of those who attended at least one session of psychological therapy but did not complete this, 64% cited a lack of adjustments to meet their needs as a contributing factor. Within mental health services, 76% reported that they had not been offered adjustments. Of these people, 69% reported that adjustments would have been required or helpful.

92% of participants who completed the physical health survey had seen their GP within the previous 12 months and 59% had seen a specialist or attended a specialist clinic in the previous

For peer review only - http://bmjopen.bmj.com/site/about/guidelines.xhtml

two years. 59% of respondents reported a medical condition that required them to attend regular medical appointments. Of these people, 24% reported missing at least one appointment. Many people (45%) reported issues related to the sensory environment, mental health conditions (e.g. anxiety about attending appointments) and dissatisfaction with healthcare settings (e.g. poor accessibility) as reasons for missing appointments. Within physical health services, 80% reported that they had not been offered adjustments. Of these people, 56% reported that adjustments would have been required or helpful.

Categorising key adjustments to services

Exploratory factor analysis (EFA) supported the retention of between two and four factors. Using an a-priori loading criterion of .4 a clear three-factor solution was retained in the mental health data (factor loadings of >.4 for 17/18 items; intercorrelations: .69 to .79). The factors related to adjustments for 1) 'sensory environment', 2) 'clinical and service context' and 3) 'clinician knowledge and communication'. Item 16 '*short waiting times to be seen when you attend appointments*' did not load clearly onto any factor. This solution demonstrated very good/excellent model fit across all tested indices. A four-factor solution was most interpretable for the physical health data (factor loadings of > .4 for 15/18 items and good model fit). Closer inspection indicated strong similarities across both solutions. Thirteen out of 18 adjustments loaded onto the same, or similar factors. Data for the item relating to shorter waiting times are presented alone (see Supplementary tables 2, 3 and 4 for factor loadings and model fit indices). The importance (Table 2) and availability (Table 3) of adjustments are presented in the categories identified by factor analysis.

[INSERT TABLE 2]

For peer review only - http://bmjopen.bmj.com/site/about/guidelines.xhtml

Most respondents reported that all adjustments were 'somewhat important' or 'very important' (hereafter referred to as important) in both mental and physical health services. In mental health services, those rating each adjustment as important ranged from 46% (item 3) to 98% (item 1). In physical health services, these ranged from 47% (item 4) to 97% (item 1). High proportions of participants also rated each adjustment as 'very important' ranging from 23% (item 3) to 88% (item 1) in mental health services and 18% (item 4) to 81% (item 1) in physical health services.

[INSERT TABLE 3]

For over half of respondents all but three adjustments were endorsed as 'rarely or never available' across both health service contexts. Adjustments were frequently endorsed as being 'never available' across both settings, ranging from 10% to 63% in mental health and 13% to 67% in physical health (both item 18 and item 7 respectively).

Paired comparison of the importance and availability of adjustments between physical and mental health services

221 participants completed both surveys. After Bonferroni correction, only one adjustment was rated as being very important significantly more often in mental health (65%) compared with physical health settings (47%): *'a clinician who uses an approach which is informed by what you have said that you prefer'* ($p = .001$). No significant differences in the least available adjustments based on service type were observed. Full reporting of these analyses can be found in Supplementary Tables 4 and 5.

Other adjustments endorsed by autistic people

For peer review only - http://bmjopen.bmj.com/site/about/guidelines.xhtml

16% of participants in the mental health survey and 14% in the physical health survey gave information in open text about an 'other' adjustment; some of these were not coded (e.g. general comments). The majority (90% mental health, 91% physical health) of open text comments were classified within the 3-factor solution (see Table 4). An additional adjustment *"Being able to bring a supporter such as a friend, family member (or support animal) to appointments"* was suggested by several participants in both health service contexts.

[INSERT TABLE 4]

Discussion

This large study successfully identified for the first time autistic adults' views about the importance and availability of key adjustments that UK and international guidelines and research consider could improve accessibility and acceptability of healthcare services. Autistic adults strongly endorsed the importance of these adjustments; the relative importance of each adjustment was broadly equivalent across physical and mental health service contexts. However, autistic people experienced limited availability to key adjustments across both healthcare contexts – showing clear unmet clinical need. Given that autistic people are at increased risk of combinations of mental and physical health conditions, health care providers need to take action now to implement provision of adjustments to improve service accessibility.

Three distinct but inter-correlated factors of adjustments were identified: 1) the sensory environment, 2) the clinical and service context and 3) clinician knowledge and understanding. These factors are consistent with recent syntheses of the evidence regarding the barriers to healthcare access for autistic people (12, 13) and recent NHS reasonable adjustment guidance (26), which include adjustments under the categories of environment,

For peer review only - http://bmjopen.bmj.com/site/about/guidelines.xhtml

service delivery, and communication. The majority of ‘other’ adjustments proposed by participants classified within the three-factor model, providing further validation. Participants also identified across both service contexts the benefit of having the opportunity to attend appointments with support (a friend, family member). In common with all the adjustments included in our survey, this adjustment is not on the face of it difficult to implement, and it is likely most services would be supportive if asked. The NHS reasonable adjustments policy does already include having support at appointments amongst the potential adjustments offered (26) and recommends that such a request should be discussed and noted.

The adjustment rated as being most important in both settings was ‘access to a clinician who understands autism’. Indeed, the four most important adjustments endorsed across both healthcare settings related to the ‘clinician knowledge and communication’ factor. This finding is in keeping with previous findings that healthcare professionals may lack skills and/or confidence in delivering interventions to autistic people (17). When the priority adjustments were compared across the paired sample, only one adjustment ‘*A clinician who uses an approach which is informed by what you have said that you prefer (e.g. formal or informal)’* was significantly different between healthcare contexts. Perhaps there is a perception that mental healthcare clinicians need to take into account individual preference; however, coming to a shared understanding and approach to healthcare is critical in any clinical setting. The discrepancy between the rated importance of the identified adjustments and their availability in both physical and mental health services makes stark reading. Our findings highlight the wide ‘gap’ in the availability of adjustments reported as important. Encouragingly two adjustments [*appointments at a familiar location* and with a *familiar clinician*] were rated as being available more than rarely by over half of each sample. Offering consistency in clinical care was appreciated in both contexts. In keeping with

For peer review only - http://bmjopen.bmj.com/site/about/guidelines.xhtml

recently published findings identifying widespread barriers to healthcare access for autistic people (12, 13), respondents consistently rated their experiences of not being able to access adjustments in healthcare. If autistic adults are not experiencing these adjustments to promote accessibility, they are likely to continue to experience poor treatment outcomes (20), significant inequalities and premature mortality (11).

Strengths and Limitations

One limitation of our study is that some participants reported that they did not have sufficient knowledge to comment on the availability of adjustments and were not able to provide a rating. However, the use of paired comparisons provides more statistical power than between group analyses. Further, that a notable proportion of the sample were not informed of the availability of adjustments they might want, is an important finding in itself. Our data are based on the reported experiences of the respondents. We did not aim to collect information directly from the services, and this should be done in future studies. However, the samples are large and recruited from diverse sources across the UK including the NHS, private and charity providers, so it is unlikely systematic bias was introduced. The participants in the mental health sample were all individuals who had self-reported either a diagnosed or suspected anxiety condition (relating to the aims of the original study). This may have the potential to limit the generalisability of the findings for other mental health settings – although many mental health services have a similar service design, and clinicians tend to deliver care for a variety of mental health conditions.

Our study also has many strengths. The research aimed to match published autism community research priorities and focus directly on the experiences of autistic people (18). The study also includes experiences about adjustments in both mental and physical healthcare settings. Our

For peer review only - http://bmjopen.bmj.com/site/about/guidelines.xhtml

large samples were both approximately equal in terms of male and female participants, which is a strength considering the low numbers of female participants often observed in autism research. Our data driven approach enabled us to determine key overarching factors or categories of adjustments that could be used to inform both policy and practice in relation to service development.

Implications and future research

The findings of this study highlight the lack of key adjustments available to autistic adults currently accessing physical and mental healthcare. These adjustments do not require expensive technology or equipment but rather ongoing staff training, attention to aspects of the clinical settings and flexibility in ways of delivering clinical care. We consider that 'how' and 'what' adjustments can be made should be more explicit in pre-appointment information, and during appointments. Focussing on the sensory environment, clinical and service context, clinician knowledge and communication and involvement of supporters, may help service users, providers and commissioners of services to identify and address any barriers to implementation. Proactively considering these adjustments with autistic people is in our opinion a prudent and logical first step towards minimising the barriers to healthcare. This in turn is a crucial component of an individualised /personalised approach to assessment and management of identified healthcare needs in order to maximise treatment effectiveness and reduce health inequalities. Our current research is developing and evaluating personalised mental and physical healthcare provision for autistic people across the lifespan. Examples include the co-design (with autistic people, relatives and clinicians) and evaluation of a personalised, flexible psychological intervention for anxiety in autistic adults (PAT-A, 31). We have also co-designed an intervention to investigate how autistic people age 50 and over can be encouraged to access the healthcare they need (Trial registration: ISRCTN75726745). To

For peer review only - http://bmjopen.bmj.com/site/about/guidelines.xhtml

address the primary care needs of autistic adults, we have co-designed and are evaluating the use of primary care healthchecks for autistic people (https://research.ncl.ac.uk/autismhealthchecks/). All these approaches systematically gather information about the adjustments required to maximise the chances of success (details of the tools used to do this available on request). Co-designed approaches to healthcare that meets the needs of autistic people and clinicians should increase the opportunities for autistic people to access the personalised and precision health care that is increasingly available to all people in mental and physical health services. In turn, that will hopefully reduce the increased and premature morbidity and average earlier mortality for autistic people.

Author Details

Samuel Brice[1], Jacqui Rodgers[1], Barry Ingham[1,2], David Mason[1], Colin Wilson[1], Mark Freeston[3], Ann Le Couteur[1] & Jeremy R. Parr[1,2]

1. Population Health Sciences Institute, Newcastle University, UK
2. Cumbria, Northumberland, Tyne and Wear NHS Foundation Trust, UK
3. School of Psychology, Newcastle University, UK

Corresponding Author

Professor Jeremy R Parr

Email: Jeremy.Parr@ncl.ac.uk

Address: Level III, Sir James Spence Institute, Royal Victoria Infirmary, Queen Victoria Road, Newcastle upon Tyne, NE1 4LP

For peer review only - http://bmjopen.bmj.com/site/about/guidelines.xhtml

Author contributions

JP, BI and JR conceptualised the adjustments study. JP and JR received funding for PAT-A. BI and JP received funding for IHOAP. JP and BI liaised with NHSE about adjustments. JR and MF supervised statistical analyses. SB and DM are researchers for PAT-A and IHOAP respectively and led data collection and undertook analysis. CW, MF and ALC provided comment on PAT-A and adjustment study design and analyses. All authors contributed to adjustments study design, and writing of the manuscript, and reviewed the final version.

Declarations of Interest

MF reports personal fees from Honoraria from training organisations in the area of psychological therapies, personal fees from Royalties from books in the area of psychological therapies, outside of the submitted work. All other authors declare no conflicts of interest.

Acknowledgements

We are grateful to: all the participants who gave their time to complete the PAT-A and IHOAP surveys; the IHOAP investigators; the ASC-UK team including Dr Alex Petrou (who supported data collection), and the ASC-UK investigators, including Professor Helen McConachie. The Sponsor for the studies was Cumbria, Northumberland, Tyne and Wear NHS Foundation Trust.

Funding

Data from autistic adults were collected as part of two studies:

1. Personalised Anxiety Treatment for Autistic Adults study funded by the UK autism research charity Autistica. Funding was awarded to JP and JR as part of the autism spectrum adulthood and ageing research programme at Newcastle University.

For peer review only - http://bmjopen.bmj.com/site/about/guidelines.xhtml

2. Improving the Health of Older Autistic People study, funded by the Inge Wakehurst Trust (awarded to BI and JP)

Data Availability

The data that support the findings of this study are available on reasonable request from the corresponding author, [JP]. The data are not publicly available due to containing information that could compromise the privacy of research participants.

License Statement

I, the Submitting Author has the right to grant and does grant on behalf of all authors of the Work (as defined in the below author licence), an exclusive licence and/or a non-exclusive licence for contributions from authors who are: i) UK Crown employees; ii) where BMJ has agreed a CC-BY licence shall apply, and/or iii) in accordance with the terms applicable for US Federal Government officers or employees acting as part of their official duties; on a worldwide, perpetual, irrevocable, royalty-free basis to BMJ Publishing Group Ltd ("BMJ") its licensees and where the relevant Journal is co-owned by BMJ to the co-owners of the Journal, to publish the Work in BMJ Open and any other BMJ products and to exploit all rights, as set out in our licence.

The Submitting Author accepts and understands that any supply made under these terms is made by BMJ to the Submitting Author unless you are acting as an employee on behalf of your employer or a postgraduate student of an affiliated institution which is paying any applicable article publishing charge ("APC") for Open Access articles. Where the Submitting Author wishes to make the Work available on an Open Access basis (and intends to pay the relevant

For peer review only - http://bmjopen.bmj.com/site/about/guidelines.xhtml

APC), the terms of reuse of such Open Access shall be governed by a Creative Commons licence – details of these licences and which Creative Commons licence will apply to this Work are set out in our licence referred to above.

References

1. American Psychiatric Association. Diagnostic and Statistical Manual of mental disorders (5th ed.). . Washington: APA; 2013.
2. Brugha TS, McManus S, Bankart J, Scott F, Purdon S, Smith J, et al. Epidemiology of autism spectrum disorders in adults in the community in England. Arch Gen Psychiatry. 2011;68(5):459-65.
3. Rydzewska E, Hughes-McCormack LA, Gillberg C, Henderson A, MacIntyre C, Rintoul J, et al. Prevalence of long-term health conditions in adults with autism: observational study of a whole country population. BMJ Open. 2018;8(8):e023945.
4. Hand BN, Angell AM, Harris L, Carpenter LA. Prevalence of physical and mental health conditions in Medicare-enrolled, autistic older adults. Autism. 2019;24(3):755-64.
5. Lawson LP, Richdale AL, Haschek A, Flower RL, Vartuli J, Arnold SR, et al. Cross-sectional and longitudinal predictors of quality of life in autistic individuals from adolescence to adulthood: The role of mental health and sleep quality. Autism. 2020;24(4):954-67.
6. Mason D, McConachie H, Garland D, Petrou A, Rodgers J, Parr JR. Predictors of quality of life for autistic adults. Autism Res. 2018;11(8):1138-47.
7. Croen LA, Zerbo O, Qian Y, Massolo ML, Rich S, Sidney S, et al. The health status of adults on the autism spectrum. Autism. 2015;19(7):814-23.
8. Hollocks MJ, Lerh JW, Magiati I, Meiser-Stedman R, Brugha TS. Anxiety and depression in adults with autism spectrum disorder: a systematic review and meta-analysis. Psychol Med. 2019;49(4):559-72.
9. Hudson CC, Hall L, Harkness KL. Prevalence of Depressive Disorders in Individuals with Autism Spectrum Disorder: a Meta-Analysis. J Abnorm Child Psychol. 2019;47(1):165-75.
10. Trembath D, Germano C, Johanson G, Dissanayake C. The Experience of Anxiety in Young Adults With Autism Spectrum Disorders. Focus on Autism and Other Developmental Disabilities. 2012;27(4):213-24.
11. Hirvikoski T, Mittendorfer-Rutz E, Boman M, Larsson H, Lichtenstein P, Bölte S. Premature mortality in autism spectrum disorder. British Journal of Psychiatry. 2016;208(3):232-8.
12. Bradshaw P, Pellicano E, van Driel M, Urbanowicz A. How Can We Support the Healthcare Needs of Autistic Adults Without Intellectual Disability? Current Developmental Disorders Reports. 2019;6(2):45-56.
13. Mason D, Ingham B, Urbanowicz A, Michael C, Birtles H, Woodbury-Smith M, et al. A Systematic Review of What Barriers and Facilitators Prevent and Enable Physical Healthcare Services Access for Autistic Adults. Journal of Autism and Developmental Disorders. 2019;49(8):3387-400.
14. Doherty M, Sullivan JD, Neilson SD. Barriers to healthcare for autistic adults: Consequences & policy implications. A cross-sectional study. medRxiv. 2020:2020.04.01.20050336.

For peer review only

For peer review only - http://bmjopen.bmj.com/site/about/guidelines.xhtml

15. Maisel ME, Stephenson KG, South M, Rodgers J, Freeston MH, Gaigg SB. Modeling the cognitive mechanisms linking autism symptoms and anxiety in adults. J Abnorm Psychol. 2016;125(5):692-703.
16. Nicolaidis C, Raymaker DM, Ashkenazy E, McDonald KE, Dern S, Baggs AE, et al. "Respect the way I need to communicate with you": Healthcare experiences of adults on the autism spectrum. Autism : the international journal of research and practice. 2015;19(7):824-31.
17. McCormack G, Dillon AC, Healy O, Walsh C, Lydon S. Primary Care Physicians' Knowledge of Autism and Evidence-Based Interventions for Autism: A Systematic Review. Review Journal of Autism and Developmental Disorders. 2019.
18. James Lind Alliance. Autism Top Ten 2016 [Available from: http://www.jla.nihr.ac.uk/priority-setting-partnerships/autism/top-10-priorities/.
19. Crane L, Adams F, Harper G, Welch J, Pellicano E. 'Something needs to change': Mental health experiences of young autistic adults in England. Autism. 2018:1362361318757048.
20. Nicolaidis C, Raymaker D, McDonald K, Dern S, Boisclair WC, Ashkenazy E, et al. Comparison of healthcare experiences in autistic and non-autistic adults: a cross-sectional online survey facilitated by an academic-community partnership. J Gen Intern Med. 2013;28(6):761-9.
21. National Development Team for Inclusion. Green Light Toolkit: A Guide to Auditing and Improving your Mental Health Service so that it is Effective in Supporting People with Autism and People with Learning Disabilities. 2017 [Available from: https://www.ndti.org.uk/uploads/files/Green_Light_Toolkit_2017.pdf.
22. National Institute for Health and Care Excellence. Autism spectrum disorder in adults: diagnosis and management. Clinical guideline [CG142]. 2012.
23. NHS England. The NHS Long Term Plan 2019 [Available from: https://www.longtermplan.nhs.uk/wp-content/uploads/2019/08/nhs-long-term-plan-version-1.2.pdf.
24. Gruber JNCCP. Social Responsiveness Scale–Second Edition (SRS-2). Torrance, CA: Western Psychological Services.; 2012.
25. Qualtrics. Qualtrics. Provo, Utah, USA2005.
26. NHS Digital. Reasonable Adjustment Flag [Available from: https://digital.nhs.uk/services/reasonable-adjustment-flag.
27. Mandell DS, Lawer LJ, Branch K, Brodkin ES, Healey K, Witalec R, et al. Prevalence and correlates of autism in a state psychiatric hospital. Autism. 2012;16(6):557-67.
28. Lorenzo-Seva U, Ferrando PJ. FACTOR 9.2: A Comprehensive Program for Fitting Exploratory and Semiconfirmatory Factor Analysis and IRT Models. Applied Psychological Measurement. 2013;37(6):497-8.
29. Srivastava A, Thomson SB. Framework analysis: a qualitative methodology for applied policy research. Journal of Administration and Governance. 2009;72.
30. Simes RJ. An Improved Bonferroni Procedure for Multiple Tests of Significance. Biometrika. 1986;73(3):751-4.
31. Parr JR, Brice S, Welsh P, Ingham B, Le Couteur A, Evans G, et al. Treating anxiety in autistic adults: study protocol for the Personalised Anxiety Treatment–Autism (PAT-A©) pilot randomised controlled feasibility trial. Trials. 2020;21(1):265.

For peer review only - http://bmjopen.bmj.com/site/about/guidelines.xhtml

Table 1: Comparison of the demographic characteristics of participants included in the mental health and physical health samples

Variable	Mental Health Sample (PAT-A study)	Physical Health Sample (IHOAP study)
**Total N**	537	407
**Gender N (%)**		
Male	234 (43.6)	167 (41.0)
Female	281 (52.3)	227 (55.8)
Other/rather not say	22 (4.1)	13 (3.2)
**Age: Mean (SD) [Range]**	41.3 (13.8) [18.0-77.0]	44.4 (13.4) [18.0-79.0]
**Ethnicity N (%)**		
White British	501 (93.3)	374 (91.9)
Asian	3 (0.6)	1 (0.2)
Black	2 (0.4)	3 (0.7)
Mixed	10 (1.9)	8 (2.0)
Other/rather not say	8 (1.5)	7 (1.7)
Not reported	13 (2.4)	14 (3.4)
**Highest education N (%)**		
Postgraduate degree	94 (17.5)	72 (17.7)
Bachelor's degree	135 (25.1)	97 (23.8)
Diploma of higher education	34 (6.3)	28 (6.9)
Certificate of higher education	18 (3.4)	17 (4.2)
A-level	88 (16.4)	76 (18.7)
GCSE	108 (20.1)	71 (17.4)
Basic skills	20 (3.7)	10 (2.5)
No formal qualifications	32 (6.0)	30 (7.4)
Other	8 (1.5)	6 (1.5)
**Employment status N (%)**		
Employed without support[a]	213 (39.7)	154 (37.8)

For peer review only

Employed with support	9 (1.7)	5 (1.2)
Volunteer	49 (9.1)	38 (9.3)
Unemployed	170 (31.7)	137 (33.7)
Retired	30 (5.6)	25 (6.1)
Other	58 (10.8)	38 (9.3)
No response	8 (1.5)	10 (2.5)
**ASD diagnosis N (%)**		
Formal diagnosis	443 (82.5)	341 (83.8)
Suspected/unsure/awaiting assessment	94 (17.5)	66 (16.2)
**Age at diagnosis: Mean (SD) [Range]**	35.8 (15.8) [3.0-73.0]	38.7 (14.8) [2.0-68.0]
**SRS score[b] by severity category N (%)**		
Normal	20 (4.2)	9 (2.5)
Mild	45 (9.4)	35 (9.7)
Moderate	172 (35.8)	115 (31.9)
Severe	244 (50.7)	201 (55.8)
Mean (SD)	110.5 (25.6)	115.1 (25.8)
**Support received N (%)**		
Home	152 (15.5)	115 (15.2)
Employment	50 (5.1)	36 (4.8)
Health	110 (11.2)	95 (12.6)
Finance	129 (13.2)	99 (13.1)
Social	75 (7.7)	57 (7.5)
Lifelong learning	67 (6.9)	47 (6.2)
Community	78 (8.0)	68 (9.0)
Organisation	73 (7.5)	55 (7.3)
Do not receive support	244 (24.9)	183 (24.2)
**Mental health condition N (%)**		
Diagnosis	-	351 (86.2)
No diagnosis or suspected	-	56 (13.8)
**Physical health condition N (%)**		

For peer review only

Diagnosis	397 (73.9)	-
No diagnosis or suspected	140 (26.1)	-

a- includes self-employed; b- not available for all participants; c- may add to more than total sample size as multiple responses were allowed

For peer review only

Table 2. The importance of key adjustments to mental and physical health services to meet the needs of autistic adults

Key adjustment	Mental Health Services		Physical Health Services	
	Important [a] N (%)	Very important N (%)	Important [a] N (%)	Very important N (%)
**F1: Sensory Environment**				
9. Change the sensory environment in the building that the appointment will take place in	387 (73.0)	209 (39.4)	289 (74.0)	166 (42.5)
10. Locations (e.g. waiting rooms) with small numbers of people	430 (81.3)	268 (50.7)	324 (82.2)	208 (52.8)
11. Locations with low noise levels	471 (89.1)	322 (60.9)	348 (88.3)	244 (61.9)
12. Locations with low light levels	338 (64.3)	185 (35.2)	259 (67.0)	138 (35.7)
**F2: Clinical and Service Context**				
2. Changing the length of appointments to suit you	375 (70.0)	194 (36.2)	311 (78.7)	177 (44.8)
3. Offering appointments online or via apps	240 (45.8)	120 (22.9)	194 (50.0)	121 (31.2)
4. Changing how often you are asked to attend appointments	293 (55.2)	130 (24.5)	178 (46.7)	69 (18.1)
5. Give information to the clinician pre-appointment so that they can prepare	469 (88.3)	322 (60.6)	320 (80.8)	190 (48.0)
17. Provide support in relation to attending appointments (e.g. managing fears or uncertainties which might make attending difficult)	428 (81.2)	282 (53.5)	307 (78.9)	198 (50.9)
18. Appropriate distractions provided whilst waiting to be seen at appointment (e.g. tablet with headphones)	253 (48.3)	132 (25.2)	199 (52.0)	106 (27.7)
**F3: Clinician Knowledge and Communication**				
1. Clinicians who understand autism	524 (98.3)	467 (87.6)	386 (96.5)	322 (80.5)
6. Opportunity after the appointment to ask questions about conclusions	487 (91.3)	321 (60.2)	353 (89.8)	253 (64.4)
7. Appointments at an easily identified and accessible location	478 (90.0)	374 (70.4)	351 (88.8)	281 (71.7)
8. Appointments with an easily identified and familiar clinician	477 (90.0)	365 (68.9)	357 (90.8)	279 (71.0)
13. Having a health summary document which can be shared with clinicians (e.g. hospital passport)	420 (79.9)	268 (51.0)	288 (74.8)	188 (48.8)
14. A clinician who uses an approach which is informed by what you have said that you prefer (e.g. formal or informal)	485 (91.8)	318 (60.2)	311 (80.6)	193 (50.0)
15. Identifying reasons that make it difficult to see a clinician or attend an appointment	434 (82.6)	260 (49.5)	316 (82.7)	202 (52.9)
**Standalone item**				
16. Short waiting times to be seen when you attend appointments	461 (86.7)	293 (55.1)	338 (86.0)	227 (57.8)

a 'Important shows the number and percentage of participants who rated each adjustment as either 'somewhat important' or 'very important'

Table 3: The lack of availability of key adjustments to mental and physical healthcare services to meet the needs of autistic adults

Key adjustment	Mental Health Services		Physical Health Services	
	Rarely or never available N (%)	Never available N (%)	Rarely or never available N (%)	Never available N (%)
**Sensory Environment**				
9. Change the sensory environment in the building that the appointment will take place in	171 (72.2)	108 (45.6)	199 (83.6)	144 (60.5)
10. Locations (e.g. waiting rooms) with small numbers of people	146 (51.6)	83 (29.3)	203 (76.1)	131 (49.1)
11. Locations with low noise levels	144 (52.0)	72 (26.0)	189 (69.4)	116 (42.6)
12. Locations with low light levels	161 (65.2)	101 (40.9)	205 (83.3)	129 (52.4)
**Clinical and Service Context**				
2. Changing the length of appointments to suit you	154 (68.4)	102 (45.3)	164 (70.7)	96 (41.4)
3. Offering appointments online or via apps	126 (64.6)	94 (48.2)	117 (50.2)	76 (32.6)
4. Changing how often you are asked to attend appointments	96 (53.0)	50 (27.6)	99 (67.3)	65 (44.2)
5. Give information to the clinician pre-appointment so that they can prepare	123 (56.1)	73 (33.3)	148 (66.4)	92 (41.3)
17. Provide support in relation to attending appointments (e.g. managing fears or uncertainties which might make attending difficult)	146 (62.1)	95 (40.4)	167 (76.6)	100 (45.9)
18. Appropriate distractions provided whilst waiting to be seen at appointment (e.g. tablet with headphones)	181 (79.0)	145 (63.3)	176 (84.6)	138 (66.3)
**Clinician Knowledge and Communication**				
1. Clinicians who understand autism	186 (71.8)	81 (31.3)	170 (73.0)	86 (36.9)
6. Opportunity after the appointment to ask questions about conclusions	113 (42.8)	50 (18.9)	121 (47.0)	69 (26.8)
7. Appointments at an easily identified and accessible location	78 (25.4)	31 (10.1)	93 (25.0)	38 (13.0)
8. Appointments with an easily identified and familiar clinician	89 (31.2)	37 (13.0)	120 (41.4)	47 (16.1)
13. Having a health summary document which can be shared with clinicians (e.g. hospital passport)	134 (69.8)	100 (52.1)	150 (81.5)	117 (63.6)
14. A clinician who uses an approach which is informed by what you have said that you prefer (e.g. formal or informal)	129 (51.4)	59 (23.5)	136 (62.9)	91 (42.1)
15. Identifying reasons that make it difficult to see a clinician or attend an appointment	127 (59.9)	72 (34.0)	144 (72.8)	92 (46.5)
**Standalone item**				
16. Short waiting times to be seen when you attend appointments	150 (52.3)	78 (27.2)	199 (69.6)	102 (35.7)

For peer review only

Table 4. Framework analysis of open text comments

	Mental Health Services	Physical Health Services
**Sensory Environment**	8	5
**Clinical and Service Context**	33	24
**Clinician Knowledge & Communication**	16	10
**Other**	6	4

For peer review only

Supplementary Information

Supplementary Table 1: Demographic information for the mental health and physical health samples and comparison with non-responders

	Mental Health (PAT-A)		Physical Health (IHOAP)	
**Variable**	**Responders**	**Non-responders**	**Responders**	**Non-responders**
Total N	537	545	407	484
Gender N (%)				
Male	234 (43.6)	273 (50.1)	167 (41.0)	238
Female	281 (52.3)	254 (46.6)	227 (55.8)	236
Other/rather not say	22 (4.1)	18 (3.3)	13 (3.2)	10
Age: Mean (SD) [Range]	41.3 (13.8) [17.0-77.0]	37.6 (12.9) [17.0-84.0]	44.4 (13.4) [18.0-79.0]	41.0 (13.3) [18.0-90.0]
Ethnicity				
White	501 (93.3)	496 (91.0)	374 (91.9)	437 (90.3)
Asian	3 (0.6)	8 (1.5)	1 (0.2)	6 (1.2)
Black	2 (0.4)	3 (0.6)	3 (0.7)	2 (0.4)
Mixed	10 (1.9)	10 (1.8)	8 (2.0)	11 (2.3)
Other/rather not say	8 (1.5)	4 (0.7)	7 (1.7)	6 (1.2)
Not reported	13 (2.4)	24 (4.4)	14 (3.4)	26 (5.4)
Highest education				
Postgraduate degree	94 (17.5)	67 (12.3)	72 (17.7)	69 (14.3)
Bachelor's degree	135 (25.1)	122 (22.4)	97 (23.8)	97 (20.0)
Diploma of higher education	34 (6.3)	43 (7.9)	28 (6.9)	52 (10.7)
Certificate of higher education	18 (3.4)	27 (5.0)	17 (4.2)	19 (3.9)
A-level	88 (16.4)	115 (21.1)	76 (18.7)	93 (19.2)
GCSE	108 (20.1)	86 (15.8)	71 (17.4)	85 (17.6)
Basic skills	20 (3.7)	27 (5.0)	10 (2.5)	24 (5.0)
No formal qualifications	32 (6.0)	34 (6.2)	30 (7.4)	36 (7.4)
Other	8 (1.5)	24 (4.4)	6 (1.5)	9 (1.9)
Employment status N (%)				
Employed without support[a]	213 (39.7)	204 (37.4)	154 (37.8)	163 (33.7)
Employed with support	9 (1.7)	8 (1.5)	5 (1.2)	10 (2.1)
Volunteer	49 (9.1)	55 (10.1)	38 (9.3)	10 (2.1)
Unemployed	170 (31.7)	188 (34.5)	137 (33.7)	202 (41.7)
Retired	30 (5.6)	20 (3.7)	25 (6.1)	24 (5.0)
Other	58 (10.8)	57 (10.5)	38 (9.3)	62 (12.8)
No response	8 (1.5)	13 (2.4)	10 (2.5)	13 (2.7)
ASD diagnosis N (%)				
Formal diagnosis	443 (82.5)[d]	467 (85.7)	341 (83.8)[e]	415 (85.7)
Suspected/unsure/awaiting diagnosis	94 (17.5)[d]	78 (14.3)	66 (16.2)[e]	69 (14.3)
Age at diagnosis: Mean (SD) [Range]	35.8 (15.8) [3.0-73.0]	30.1 (15.5) [2.0-69.0]	38.7 (14.8) [2.0-68.0]	34.4 (15.8) [2.0-86.0]
SRS severity N (%)				
Normal	20 (4.2)	27 (7.2)	9 (2.5)	19 (5.7)
Mild	45 (9.4)	35 (9.3)	35 (9.7)	31 (9.2)
Moderate	172 (35.8)	112 (29.8)	115 (31.9)	97 (28.9)
Severe	244 (50.7)	202 (53.7)	201 (55.8)	189 (56.3)
SRS Mean (SD)	110.5 (25.6)	112.9 (29.2)	115.1 (25.8)	115.6 (28.2)
Support received[c] N (%)				
Home	152 (15.5)	162 (13.6)	115 (15.2)	142 (17.4)
Employment	50 (5.1)	78 (6.5)	36 (4.8)	53 (6.5)
Health	110 (11.2)	153 (12.8)	95 (12.6)	130 (16.0)
Finance	129 (13.2)	154 (12.9)	99 (13.1)	137 (16.8)
Social	75 (7.7)	115 (9.6)	57 (7.5)	88 (10.8)
Lifelong learning	67 (6.9)	75 (6.3)	47 (6.2)	60 (7.4)

For peer review only

Community	78 (8.0)	110 (9.2)	68 (9.0)	79 (9.7)
Organisation	73 (7.5)	110 (9.2)	55 (7.3)	93 (11.4)
Do not receive support	244 (24.9)	236 (19.8)	183 (24.2)	32 (3.9)
Mental health condition N (%)				
Diagnosis	-	-	351 (86.2)	414 (85.5)
No diagnosis	-	-	56 (13.8)	70 (14.5)
Physical health condition N (%)				
Diagnosis	397 (73.9)	402 (73.8)	-	-
No diagnosis	140 (26.1)	143 (26.2)	-	-
Response type				
Electronic	415 (77.3%	446 (81.8)	305 (74.9)	396 (81.8)
Paper	122 (22.7%)	99 (18.2)	102 (25.1)	88 (18.2)

Tests of difference between responders and non-responders on key demographic variables

Significant differences are denoted with *

Age:
Mental health sample: $t(859)= 1.655$, $p=0.949$
Physical health sample: $t(668)= 3.369$, $p=0.001$*

Gender:
Mental health sample: M,F,O,R genders Mental health sample vs Physical health sample $\chi^2(3)=2.049$, $p=0.562$; males and females only: $\chi^2(1)=0.349$, $p=0.555$
Physical health sample: M,F,O,R genders Mental health sample vs Physical health sample $\chi^2(3)=4.481$, $p=0.214$; males and females only: $\chi^2(1)=1.007$, $p=0.316$

Autism Characteristics (SRS total):
Mental health sample: $t(641.5)= -1.135$, $p=0.257$
Physical health sample: $t(496)= -0.198$, $p=0.843$

For peer review only

Exploratory Factor Analysis on participants' importance of adjustment ratings

Exploratory factor analysis (EFA) was conducted in order to assess whether the adjustments clustered together in a meaningful way. FACTOR software version 10.8.04 (1) was used to conduct EFA on data on the importance of each adjustment from both datasets from study 1 and study 2. Polychoric correlations were used as this is deemed most appropriate for use with ordinal and/or skewed data such as these (2). Unweighted least squares were used to extract the factors and the rotation method was Promin. The use of an oblique method of rotation such as this allows for the correlation between factors to be calculated, which is in line with best scientific practice (3). Bias correlated bootstrap resampling was undertaken using 5000 bootstrap samples. This decision was taken in order to reduce the influence of any outliers and maximise the generalisability of the sample. Model fit was assessed using a number of indices including Root Mean Square Error of Approximation (RMSEA), Non-Normed Fit Index (NNFI), Comparative Fit Index (CFI), Goodness of Fit Index (GFI) and Root Mean Square of Residuals (RMSR).

Participants with at least one missing response on any of the importance of adjustment ratings were deleted listwise prior to conducting EFA as any efforts to impute missing items may have biased the results at this stage. This resulted in the removal of 51 participants from the mental health dataset, leaving a sample of N = 486 and 78 participants from the physical health dataset leaving a sample of N = 329. Sample size suitability for EFA has been a matter of debate, however best practice guidelines suggests that a participant-to-item ratio of between 5:1 and 10:1 is considered to be suitable (4). Therefore, the 27:1 and 18.3/1 participant-to-item ratios in the mental health and physical health datasets respectively were deemed more than adequate for EFA. Furthermore, the Kaiser-Meyer-Olkin (KMO) test revealed that the sampling

For peer review only

adequacy was 'very good' in both datasets (mental health: KMO = .92; physical health: KMO = .91).

Exploratory factor analysis (EFA) was conducted using data from participants' ratings of the importance of each key adjustment identified in the survey EFA was conducted separately for each survey (i.e. mental health and physical health). Decisions regarding the number of factors to retain was based on best practice guidelines (4), which included consulting existing theoretical knowledge, examining the scree plots, retaining factors with an eigenvalue greater than one and conducting parallel analysis. These indicators supported the retention of between two and four factors in each of the datasets. We applied an a-priori loading criterion of ≥ .4 to the rotated loading matrices, in order to ensure that emergent factors were meaningful, whilst not being so stringent as to limit the reproducibility of the findings (5)

References

1. Lorenzo-Seva U, Ferrando PJ. FACTOR 9.2: A Comprehensive Program for Fitting Exploratory and Semiconfirmatory Factor Analysis and IRT Models. Applied Psychological Measurement. 2013;37(6):497-8.
2. Baglin J. Improving your exploratory factor analysis for ordinal data: a demonstration using FACTOR. Practical Assessment, Research and Evaluation. 2014;19.
3. Osborne JW. What is Rotating in Exploratory Factor Analysis. Practical Assessment, Research and Evaluation. 2015;20.
4. Osborne JW. Best Practices in Exploratory Factor Analysis: Jason W. Osbourne; 2014.
5. Hoyle R, Duvall J. Determining the number of factors in exploratory and confirmatory factor analysis. 2004.

For peer review only

Supplementary Table 2: Factor loadings for the three-factor solution for importance of adjustments to mental health services and four-factor solution for the importance of adjustments to physical health services

Adjustment		Mental Health			Physical Health			
	Factor Factor Code	F1 SE	F2 SC	F3 CKC	F1 SE	F2 SC	F3 CKC	F4 F
9. Change the sensory environment in the building that the appointment will take place in		**0.799**	0.064	0.006	**0.925**	-0.178	0.120	0.015
10. Locations (e.g. waiting rooms) with small numbers of people		**0.787**	-0.001	0.081	**0.621**	0.258	-0.212	0.245
11. Locations with low noise levels		**1.065**	-0.186	-0.054	**0.905**	-0.094	-0.051	0.064
12. Locations with low light levels		**0.718**	0.099	-0.055	**0.874**	0.109	0.076	-0.230
2. Changing the length of appointments to suit you		-0.100	**0.551**	0.212	-0.098	0.222	0.376	0.284
3. Offering appointments online or via apps		-0.164	**0.765**	-0.222	-0.002	**0.502**	0.009	-0.123
4. Changing how often you are asked to attend appointments		-0.062	**0.685**	-0.038	-0.145	**0.829**	0.063	-0.094
5. Give information to the clinician pre-appointment so that they can prepare		-0.159	**0.417**	0.378	-0.070	0.119	**0.671**	0.041
17. Provide support in relation to attending appointments (e.g. managing fears or uncertainties which might make attending difficult)		0.117	**0.511**	0.184	0.115	0.294	0.087	0.378
18. Appropriate distractions provided whilst waiting to be seen at appointment (e.g. tablet with headphones)		0.215	**0.680**	-0.162	0.292	**0.422**	0.001	0.039
1. Therapists who understand autism		0.047	-0.458	**0.970**	0.274	-0.044	0.282	0.202
6. Opportunity after the appointment to ask questions about conclusions		-0.244	-0.162	**0.838**	-0.137	0.010	**0.836**	-0.042
7. Appointments at an easily identified and accessible location		0.063	-0.057	**0.625**	0.026	-0.041	0.043	**0.668**
8. Appointments with an easily identified and familiar clinician		0.124	-0.110	**0.685**	-0.036	-0.253	0.026	**1.044**
13. Having a health summary document which can be shared with clinicians (e.g. hospital passport)		-0.019	0.168	**0.556**	0.143	-0.163	**0.861**	-0.188
14. A clinician who uses an approach which is informed by what you have said that you prefer (e.g. formal or informal)		0.035	0.016	**0.624**	0.066	-0.135	**0.736**	0.103
15. Identifying reasons that make it difficult to see a clinician or attend an appointment		0.096	0.193	**0.474**	0.053	0.230	**0.495**	0.041
16. Short waiting times to be seen when you attend appointments		0.268	0.169	0.295	0.002	**0.410**	-0.028	**0.421**

Key: SS = Sensory Environment, SC = Clinical and Service Context, CKC = Clinician Knowledge and Communication, F = Familiarity
Factor loadings that exceed the ≥ 0.4 loading criteria are denoted in bold

Supplementary Table 3: Inter-factors correlation matrices

	Mental Health			Physical Health			
	**F1**	**F2**	**F3**	**F1**	**F2**	**F3**	**F4**
**F1**	1.000			1.000			
**F2**	0.688	1.000		0.722	1.000		
**F3**	0.732	0.785	1.000	0.595	0.727	1.000	
**F4**				0.694	0.705	0.736	1.000

Supplementary Table 4: Model fit indices

Model fit statistic	Mental Health	Physical Health
**Root Mean Square Error of Approximation (RMSEA)**	0.032	0.032
**Non-Normed Fit Index (NNFI)**	0.994	0.995
**Comparative Fit Index (CFI)**	0.996	0.997
**Goodness of Fit Index (GFI)**	0.993	0.995
**Root Mean Square of Residuals (RMSR)**	0.0384	0.0361

For peer review only

Supplementary Table 5: Response frequencies for the importance of adjustments in both samples

Adjustment	Mental Health Services Importance Response Frequencies (N)						Physical Health Services Importance Response Frequencies (N)					
	1 Low	2	3	4	5 High	M	1 Low	2	3	4	5 High	M
Therapists who understand autism	1	0	8	57	468	4	2	4	8	65	342	6
Changing the length of appointments to suit you	19	40	102	181	194	1	12	20	52	135	177	11
Offering appointments online or via apps	89	66	128	120	120	14	65	45	84	73	121	19
Changing how often you are asked to attend appointments	32	33	173	163	130	6	51	42	110	109	69	26
Give information to the clinician pre-appointment so that they can prepare	11	17	34	147	322	6	20	13	43	130	190	11
Opportunity after the appointment to ask questions about conclusions	5	10	31	166	321	4	8	5	27	100	253	14
Appointments at an easily identified and accessible location	5	5	43	104	374	6	6	8	30	70	281	12
Appointments with an easily identified and familiar clinician	8	11	34	112	365	7	9	9	18	78	279	14
Change the sensory environment in the building that the appointment will take place in	32	28	83	178	209	7	23	23	56	123	166	16
Locations (e.g. waiting rooms) with small numbers of people	19	25	55	162	268	8	17	14	39	116	208	13
Locations with low noise levels	10	16	32	149	322	8	11	14	21	104	244	13
Locations with low light levels	43	42	103	153	185	11	20	27	81	121	138	20
Having a health summary document which can be shared with clinicians (e.g. hospital passport)	16	17	73	152	268	11	13	15	69	100	188	22
A clinician who uses an approach which is informed by what you have said that you prefer (e.g. formal or informal)	6	6	31	167	318	9	7	15	53	117	193	22
Identifying reasons that make it difficult to see a clinician or attend an appointment	13	8	70	174	260	12	10	8	48	114	202	25
Short waiting times to be seen when you attend appointments	7	20	44	168	293	5	7	14	34	111	227	14
Provide support in relation to attending appointments (e.g. managing fears or uncertainties which might make attending difficult)	20	21	58	146	282	10	18	14	50	109	198	18
Appropriate distractions provided whilst waiting to be seen at appointment (e.g. tablet with headphones)	62	66	142	121	132	14	51	31	101	93	106	25

Response option key: 1 = not at all important, 2 = not very important, 3 = neither important or unimportant, 4 = somewhat important, 5 = very important, M = missing response

For peer review only

Supplementary Table 6: Response frequencies for the availability of adjustments in both samples

Adjustment	Mental Health Services Availability Response Frequencies (N)							Physical Health Services Availability Response Frequencies (N)						
	1 Low	2	3	4	5 High	DK	M	1 Low	2	3	4	5 High	DK	M
Therapists who understand autism	81	105	32	27	14	261	17	86	84	30	23	10	146	28
Changing the length of appointments to suit you	102	52	29	32	10	292	20	96	68	25	27	16	134	41
Offering appointments online or via apps	94	32	25	26	18	321	21	76	41	35	36	45	125	49
Changing how often you are asked to attend appointments	50	46	32	37	16	335	21	65	34	21	19	8	192	68
Give information to the clinician pre-appointment so that they can prepare	73	50	31	42	23	301	17	92	56	25	29	21	139	45
Opportunity after the appointment to ask questions about conclusions	50	63	32	73	46	255	18	69	52	43	65	28	113	37
Appointments at an easily identified and accessible location	31	47	62	103	64	209	21	38	35	52	94	73	79	36
Appointments with an easily identified and familiar clinician	37	52	50	90	56	232	20	47	73	81	66	25	79	36
Change the sensory environment in the building that the appointment will take place in	108	63	26	27	13	279	21	114	55	16	14	9	124	45
Locations (e.g. waiting rooms) with small numbers of people	83	63	57	54	26	235	19	131	72	31	26	7	100	40
Locations with low noise levels	72	72	51	47	35	237	23	116	73	32	36	15	98	37
Locations with low light levels	101	60	31	36	19	261	29	129	76	12	23	6	111	50
Having a health summary document which can be shared with clinicians (e.g. hospital passport)	100	34	18	18	22	320	25	117	33	7	13	14	174	49
A clinician who uses an approach which is informed by what you have said that you prefer (e.g. formal or informal)	59	70	44	52	26	265	21	91	45	35	28	16	147	45
Identifying reasons that make it difficult to see a clinician or attend an appointment	72	55	29	37	19	300	25	92	52	23	20	11	152	57
Short waiting times to be seen when you attend appointments	78	72	43	69	25	228	22	102	97	50	29	8	79	42
Provide support in relation to attending appointments (e.g. managing fears or	95	51	32	35	22	280	22	100	67	24	12	15	136	43

For peer review only

uncertainties which might make attending difficult)														
Appropriate distractions provided whilst waiting to be seen at appointment (e.g. tablet with headphones)	145	36	14	21	13	281	27	138	38	10	9	13	137	62

Response option key: 1 = never available, 2 = rarely available, 3 = available about half the time, 4 = available most of the time, 5 = available all the time, DK = I don't know, M = missing response

For peer review only

Supplementary Table 7: McNemar tests showing a comparison of the adjustments that were most important to autistic people in mental and physical health services in a paired sample

Adjustment	N	Mental Health % very important	Physical Health % very important	P
Clinician who understands autism	217	87.6	80.2	.048
Changing the length of appointments to suit you	217	39.6	46.1	.211
Offering appointments online or via apps	206	23.8	28.6	.332
Changing how often you are asked to attend appointments	204	23.0	20.1	.526
Give information to the clinician pre-appointment so that they can prepare	214	64.5	49.5	.004
Opportunity after the appointment to ask questions about conclusions	214	62.6	64.0	.845
Appointments at an easily identified and accessible location	213	74.6	71.4	.520
Appointments with an easily identified and familiar clinician	211	69.7	72.0	.668
Change the sensory environment in the building that the appointment will take place in	210	40.0	44.8	.378
Locations (e.g. waiting rooms) with small numbers of people	213	50.2	51.2	.925
Locations with low noise levels	212	57.5	58.5	.924
Locations with low light levels	208	31.3	36.1	.373
Having a health summary document which can be shared with clinicians (e.g. hospital passport)	207	51.7	47.8	.497
A clinician who uses an approach which is informed by what you have said that you prefer (e.g. formal or informal)	206	64.6	46.6	**.001**
Identifying reasons that make it difficult to see a clinician or attend an appointment	202	47.0	51.5	.452
Short waiting times to be seen when you attend appointments	213	55.4	56.8	.848
Provide support in relation to attending appointments (e.g. managing fears or uncertainties which might make attending difficult)	208	55.8	50.0	.299
Appropriate distractions provided whilst waiting to be seen at appointment (e.g. tablet with headphones)	203	24.1	27.1	.572

* Significant P values following Simes adapted Bonferroni correction are highlighted in bold

For peer review only

Supplementary Table 8: McNemar tests showing a comparison of the adjustments that were least available to autistic people in mental and physical health services in a paired sample

Adjustment	N	Mental Health % never available	Physical Health % never available	P
Clinician who understands autism	65	29.2	40.0	.230
Changing the length of appointments to suit you	54	53.7	40.7	.296
Offering appointments online or via apps	43	46.5	34.9	.424
Changing how often you are asked to attend appointments	21	23.8	38.1	.549
Give information to the clinician pre-appointment so that they can prepare	54	35.2	38.9	.864
Opportunity after the appointment to ask questions about conclusions	72	20.8	25.0	.690
Appointments at an easily identified and accessible location	92	15.2	10.9	.503
Appointments with an easily identified and familiar clinician	82	14.6	17.1	.832
Change the sensory environment in the building that the appointment will take place in	51	60.8	51.0	.383
Locations (e.g. waiting rooms) with small numbers of people	75	32.0	49.3	.060
Locations with low noise levels	71	29.6	33.8	.711
Locations with low light levels	55	50.9	45.5	.701
Having a health summary document which can be shared with clinicians (e.g. hospital passport)	31	64.5	48.4	.302
A clinician who uses an approach which is informed by what you have said that you prefer (e.g. formal or informal)	58	29.5	31.0	.690
Identifying reasons that make it difficult to see a clinician or attend an appointment	43	48.8	46.5	1.000
Short waiting times to be seen when you attend appointments	91	29.7	36.3	.451
Provide support in relation to attending appointments (e.g. managing fears or uncertainties which might make attending difficult)	55	45.5	36.4	.442
Appropriate distractions provided whilst waiting to be seen at appointment (e.g. tablet with headphones)	51	74.5	72.5	1.000

For peer review only

The importance and availability of adjustments to improve access for autistic adults who need mental and physical healthcare: findings from a UK survey

Journal:	*BMJ Open*
Manuscript ID	bmjopen-2020-043336.R1
Article Type:	Original research
Date Submitted by the Author:	27-Oct-2020
Complete List of Authors:	Brice, Samuel; Newcastle University Faculty of Medical Sciences, Population Health Sciences Institute<br>Rodgers, Jacqui; Newcastle University Faculty of Medical Sciences, Population Health Sciences Institute<br>Ingham, Barry; Newcastle University Faculty of Medical Sciences, Population Health Sciences Institute; Cumbria Northumberland Tyne and Wear NHS Foundation Trust<br>Mason, David; Newcastle University Faculty of Medical Sciences, Population Health Sciences Institute<br>Wilson, Colin ; Newcastle University Faculty of Medical Sciences, Population Health Sciences Institute<br>Freeston, Mark; Newcastle University Faculty of Medical Sciences, School of Psychology<br>Le Couteur , Ann; Newcastle University Faculty of Medical Sciences, Population Health Sciences Institute<br>Parr, Jeremy; Newcastle University Faculty of Medical Sciences, Population Health Sciences Institute; Cumbria Northumberland Tyne and Wear NHS Foundation Trust
**Primary Subject Heading**:	Health services research
Secondary Subject Heading:	Mental health
Keywords:	MENTAL HEALTH, Quality in health care < HEALTH SERVICES ADMINISTRATION & MANAGEMENT, Organisational development < HEALTH SERVICES ADMINISTRATION & MANAGEMENT

I, the Submitting Author has the right to grant and does grant on behalf of all authors of the Work (as defined in the below author licence), an exclusive licence and/or a non-exclusive licence for contributions from authors who are: i) UK Crown employees; ii) where BMJ has agreed a CC-BY licence shall apply, and/or iii) in accordance with the terms applicable for US Federal Government officers or employees acting as part of their official duties; on a worldwide, perpetual, irrevocable, royalty-free basis to BMJ Publishing Group Ltd ("BMJ") its licensees and where the relevant Journal is co-owned by BMJ to the co-owners of the Journal, to publish the Work in this journal and any other BMJ products and to exploit all rights, as set out in our licence.

The Submitting Author accepts and understands that any supply made under these terms is made by BMJ to the Submitting Author unless you are acting as an employee on behalf of your employer or a postgraduate student of an affiliated institution which is paying any applicable article publishing charge ("APC") for Open Access articles. Where the Submitting Author wishes to make the Work available on an Open Access basis (and intends to pay the relevant APC), the terms of reuse of such Open Access shall be governed by a Creative Commons licence – details of these licences and which Creative Commons licence will apply to this Work are set out in our licence referred to above.

Other than as permitted in any relevant BMJ Author's Self Archiving Policies, I confirm this Work has not been accepted for publication elsewhere, is not being considered for publication elsewhere and does not duplicate material already published. I confirm all authors consent to publication of this Work and authorise the granting of this licence.

For peer review only

The importance and availability of adjustments to improve access for autistic adults who need mental and physical healthcare: findings from a UK survey

Authors

Samuel Brice samuel.brice@ncl.ac.uk

Jacqui Rodgers jacqui.rodgers@ncl.ac.uk

Barry Ingham barry.ingham@cntw.nhs.uk

David Mason david.mason@ncl.ac.uk

Colin Wilson colin.wilson@ncl.ac.uk

Mark Freeston mark.freeston@ncl.ac.uk

Ann Le Couteur a.s.le-couteur@ncl.ac.uk

Jeremy R. Parr jeremy.parr@ncl.ac.uk (corresponding author)

For peer review only

For peer review only - http://bmjopen.bmj.com/site/about/guidelines.xhtml

Abstract

Objectives: To investigate autistic people's views on the importance and availability of adjustments to mental and physical healthcare provision. To explore whether specific categories of adjustments can be identified and to identify any differences in their importance and availability between mental and physical healthcare.

Design: Data from two studies, both employing a cross-sectional survey design.

Setting: UK based autistic adults registered with the Adult Autism Spectrum Cohort-UK were contacted by post or online. In both studies, recruitment was staged over a 12-month period. Non-responders were sent a single reminder letter two weeks after initial contact.

Participants: 537 autistic adults completed a survey about mental health services (51% response rate), 407 completed the physical health survey (49% response rate). Within these samples, 221 participants completed both surveys.

Primary outcome measures: Each study developed a bespoke survey to explore participants' views on mental and physical health services respectively. Both included an identical list of adjustments that participants rated based on importance and availability.

Results: Three categories of important adjustments were identified: sensory environment, clinical and service context, and clinician knowledge and communication. Adjustments across healthcare settings were widely rated as being important yet rarely available. One significant difference between the importance of adjustments available through mental and physical health services was identified. Participants reported that having access to a clinician who is willing to adapt their approach to suit the person's preferences was significantly more important for participants attending mental health settings ($p = .001$).

Conclusions: Autistic people reported the limited availability of important adjustments in current healthcare provision. To address unmet need and tackle the health inequalities faced by autistic people attending physical and mental healthcare settings, healthcare providers should

For peer review only - http://bmjopen.bmj.com/site/about/guidelines.xhtml

offer adjustments relating to the three identified factors. Future research should focus on identifying and addressing service-provider barriers to implementation.

Strengths and limitations of this study

- This research matched published research priorities of the autism community and focused directly on the experiences of autistic people
- This study reports on key adjustments to both physical and mental health services
- Our data driven approach enabled us to determine overarching categories of adjustments that could be used to inform both policy and practice in relation to service development
- A notable proportion of participants reported that they did not have sufficient knowledge to comment on the availability of adjustments
- This study did not seek the opinions of health service providers, which should be a focus for future implementation research

For peer review only - http://bmjopen.bmj.com/site/about/guidelines.xhtml

Introduction

Autism Spectrum Disorder[1] is characterised by lifelong persistent difficulties with social communication and social interaction and the presence of patterns of restrictive and repetitive behaviours or interests that may include hyper or hyporeactivity to sensory aspects of the environment (1). At least 1.1% of the United Kingdom (UK) population is autistic (2).

Autistic people are more likely to experience mental and physical health conditions than the general (neurotypical) population (3); this persists across the lifespan (4). Poor health has been shown to predict poorer quality of life (5, 6). Autistic adults are more likely than neurotypical adults to experience physical health conditions, such as epilepsy, cardiovascular disease and diabetes (7). Anxiety and depression are particularly common, with an estimated lifetime prevalence of around 40% (8, 9). Autistic people are also more likely to experience multiple mental and physical health conditions concurrently (10). Epidemiological studies suggest autistic people are also significantly more likely to face premature mortality: the average life expectancy for an autistic adult without intellectual disability is 12 years lower than for neurotypical people (11).

For peer review only

Barriers to healthcare access

Autistic adults face a number of significant barriers to effective health care access and treatment (12, 13). To date, the most frequently reported barriers have been considered within three factors: individual-level (e.g. barriers to effective communication with clinicians, sensory sensitivities), provider-level (e.g. lack of 'autism awareness' amongst clinicians) and system-

[1] We will use the term 'autism' or 'autistic person' throughout this paper, reflecting a growing consensus that this terminology is preferred by the autism community. This terminology includes previously recognised subcategories of autism, including but not limited to Asperger's Disorder, Autistic Disorder and Pervasive Developmental Disorder.

For peer review only - http://bmjopen.bmj.com/site/about/guidelines.xhtml

level (e.g. convoluted referral pathways and lack of availability of specialist services). Although such attempts to categorise adjustments in this way are useful to provide a simplified message to healthcare providers, this categorisation system has yet to receive empirical support. Recent research indicates that these barriers prevent autistic people from arranging and attending healthcare appointments (14). In addition, other individual factors such as difficulties coping with the potential uncertainty of healthcare situations (15), and pre-existing anxiety (8) may make the experience of attending healthcare appointments especially problematic for autistic people. Further, although the UK National Health Service (NHS) is publicly funded and free at the point of access, in other countries the affordability of healthcare has been shown to be a barrier that is disproportionately more likely to affect autistic people (16). There is also evidence of a lack of training, skills and/or confidence among healthcare professionals in working with and delivering interventions to autistic people (17), which may impact on their continued engagement with healthcare services (12).

UK autism research priorities focus on aspects of physical and mental healthcare, including 'How should service delivery for autistic people be improved and adapted in order to meet their needs?' (18). Many autistic people report dissatisfaction with the current accessibility of healthcare across both physical and mental health settings (19, 20). Several recent UK clinical guidelines have recommended that to reduce barriers to healthcare, providers should adjust their procedures and approach to best meet the individual needs of autistic people, with additional specific guidance for mental health services, to meet the needs of people with intellectual disabilities (21-23). A recent study concluded that "adjustments for communication needs are as necessary for autistic people as ramps are for wheelchair users" (14). Evidence suggests that adjustments to services such as reducing exposure to potentially aversive sensory stimuli (e.g. noisy waiting rooms) and the clinician using the autistic adult's preferred

For peer review only - http://bmjopen.bmj.com/site/about/guidelines.xhtml

communication methods, do facilitate effective healthcare access for autistic people (13). However, the importance and availability of specific adjustments across healthcare settings is unknown.

Aims

1. To investigate autistic people's views on the importance and availability of adjustments that are designed to promote the accessibility and acceptability of mental and physical healthcare provision.

2. To explore whether specific categories of adjustments can be identified using exploratory factor analysis and to identify any differences in their importance and availability between mental and physical healthcare.

Method

Design

This study reports on data from two UK studies – one focused on anxiety (as an exemplar of a mental health condition) and the other focused on physical health conditions. Both employed a cross-sectional survey design and were undertaken in 2018-2019.

Participants

Participants for both studies were recruited via the Adult Autism Spectrum Cohort – UK (ASC-UK; https://research.ncl.ac.uk/adultautismspectrum/), a longitudinal cohort study of autistic adults. ASC-UK recruits from a wide variety of UK sources including NHS diagnostic/health services, charitable/community organisations and also receives self-referrals. In line with previous research (e.g. 6), these studies included some participants who were awaiting autism

For peer review only - http://bmjopen.bmj.com/site/about/guidelines.xhtml

assessment or suspected that they were autistic (18% and 16% of mental health and physical health samples respectively).

Participants completed paper or web-based materials, depending on their preference. Following consent, participants completed a registration questionnaire that included questions on demographics, mental and physical health, education, employment, and aspects of daily living. Participants also completed the Social Responsiveness Scale -2nd Edition (SRS-2, (24)).

The Personalised Anxiety Treatment – Autism (PAT-A) study investigated autistic people's experiences of anxiety and access to mental healthcare, with a particular focus on accessing psychological therapies, the primary treatment choice for anxiety conditions in UK clinical guidance (22). Inclusion criteria were autistic people living in the UK aged 18 years and older. 1113 autistic people aged 18 and over who had previously reported a diagnosed or suspected anxiety condition were invited to participate. 568 (51%) returned the consent form and completed the survey. Thirty-one participants consented and did not respond to any of the questions about adjustments and were not included in analyses, resulting in a final sample of 537.

The Improving the Health of Autistic People (IHOAP) study investigated the physical health of autistic people, and experiences of accessing healthcare for physical health conditions, including impact on daily life, screening services offered and taken up, and factors that made accessing health care difficult. Inclusion criteria were autistic people living in the UK aged 18 years and older. 945 autistic adults were invited to participate; 461 (49%) people consented and completed the survey. 54 participants did not respond to any survey questions about adjustments and were therefore not included in analyses, resulting in a final sample of 407.

For peer review only - http://bmjopen.bmj.com/site/about/guidelines.xhtml

There were 723 unique participants in the two studies, including 221 participants who completed both studies. Demographic information (from ASC-UK) for participants from the PAT-A and IHOAP studies is provided in Table 1.

Procedure

Eligible participants were sent an information sheet (including an 'easyread' version), consent form and survey, either by post or email depending on their contact preferences. Those preferring to be contacted online were sent a unique link based on a participant number and completed the consent form and survey using Qualtrics© (25). Participants who received materials by post completed a written consent form and survey and returned this to the research team in a prepaid envelope. Written informed consent was provided by all participants. Participants who had not responded after two weeks were sent a single reminder letter. Recruitment was staged over a 12-month period. Participation was not incentivised. All procedures were approved by the NHS Health Research Authority and a Research Ethics Committee (both Wales REC 5, Approval reference numbers: PAT-A: 18/WA/0014; IHOAP: 18/WA/0191).

Patient and public involvement (PPI)

This research is directly matched to published research priorities of the autism community (18). The ASC-UK cohort study involved autistic people at the design stage as well as in the design and delivery of ongoing associated projects. Prior to commencing both studies, PPI focus groups were convened with community stakeholders to discuss and agree to the methods and materials, including survey questions. An autistic person and parent of an autistic person are co-investigators on this research. Author CW is autistic and was involved with the analysis of

For peer review only - http://bmjopen.bmj.com/site/about/guidelines.xhtml

data and preparation of this manuscript. Our dissemination plan includes consideration of the most effective ways to share these findings with autistic people, including presentations and newsletter updates.

Measures

Both samples completed the same survey about adjustments to services. Other data were also collected as part of the PAT-A and IHOAP studies and will be reported separately (manuscripts in process). The survey questionnaires included a list of adjustments previously reported to improve access, inclusivity and appropriateness of health services, together with an option for open text responses to record any other adjustments. The list was based on published evidence (13), clinical experience and the Reasonable Adjustments Flag (NHS Digital, https://digital.nhs.uk/services/reasonable-adjustment-flag; (26)). The list of adjustments can be seen in Tables 2 and 3.

Participants were asked to rate the importance of each adjustment on a five-point Likert scale from 1) not at all important; 2) not very important; 3) neither important nor unimportant; 4) somewhat important and 5) very important. Participants also rated how available they thought each adjustment was using a five-point scale 1) never available, 2) rarely available, 3) available about half the time, 4) available most of the time, 5) available all of the time. For availability, a 6th option 'I don't know' was provided to enable those who did not feel that they had enough experience of that particular adjustment to report this, enabling the research team to discriminate between lack of experience and a missing response. Any additional adjustments identified by participants in open text response boxes were recorded.

For peer review only - http://bmjopen.bmj.com/site/about/guidelines.xhtml

The Social Responsiveness Scale – 2nd Edition (SRS-2; (24))

The SRS-2 is a widely used standardised self-report questionnaire used to characterise autism 'severity'. The SRS-2 has demonstrated excellent internal consistency and the predictive validity of the adult form has been reported to have a specificity level of .60 and a sensitivity of .86 (27). The full-scale score was used to make comparisons between those who self-reported a diagnosis of autism and those who suspected they had autism or were awaiting assessment; and between responders and non-responders (further detail in Table 1 and supplementary Table 1).

Analysis

Key demographic characteristics (age, gender and level of autism traits) were compared using independent t-tests or ANOVA for participants who self-reported an autism diagnosis, and those who suspected that they were autistic or were awaiting an autism diagnosis; responders vs non-responders (in both studies) and between participants from the mental health and physical health samples. These comparisons were made to assess the representativeness and comparability of the samples and to aid with interpretation of the findings.

Descriptive analyses of survey responses regarding prior service use in both samples were undertaken to provide further context. This information included the proportion of participants who had previously accessed mental and physical healthcare, their engagement with treatment and preliminary indications regarding their experience of adjustments to healthcare.

Exploratory factor analysis (EFA) was conducted to identify whether the adjustments could be empirically grouped in a meaningful way, using data from both datasets (FACTOR software version 10.8.04 (28). The polychoric correlation matrix was subject to unweighted least

For peer review only

For peer review only - http://bmjopen.bmj.com/site/about/guidelines.xhtml

squares extraction with Promin rotation. Bias correlated bootstrap resampling was undertaken using 5000 bootstrap samples and model fit was formally assessed. Participants with one or more missing responses on any of the importance of adjustment ratings were deleted prior to EFA, resulting in the removal of 51 participants from the mental health dataset (final N = 486), and 78 participants from the physical health dataset (N = 329). Participant-to-item ratios of 27:1 and 18:1 were deemed more than adequate; the Kaiser-Meyer-Olkin (KMO) indicated that the sampling adequacy was 'very good' in both datasets. See supplemental materials for full details.

Quantitative data were analysed descriptively. Open text responses about adjustments were analysed using framework analysis (29). One researcher (JR) coded open text responses against the adjustment categories indicated in EFA. Discrepancies were discussed and agreed with another researcher (SB). Additional adjustments that did not fit within these categories were recorded under the category 'other'.

Service-level differences (i.e. comparisons between mental healthcare and physical healthcare) in adjustments that were reported by autistic people as most important and least available were explored using McNemar's test to compare nominal data in the paired sample of 221 participants who completed the PAT-A and IHOAP surveys. Variables were first dichotomized to separate the proportion who selected that an adjustment was 'very important' compared with all other levels of importance and 'never available' compared with all other levels of availability. McNemar's comparisons were computed for each of the 18 core adjustments based on the dichotomised importance and availability ratings. Simes' modified Bonferroni correction (30) was used to minimise risk of type I error.

For peer review only - http://bmjopen.bmj.com/site/about/guidelines.xhtml

Results

For both the mental health and physical health samples, there was no significant difference between the SRS score of those who reported an autism spectrum diagnosis, and those who suspected they were autistic or who were awaiting autism assessment (Independent t-tests: mental health: 112.5 vs 112.4 respectively, $t(479)= -0.002$, $p=0.998$; physical health: 115.7 vs 112.3 respectively, $t(358)= -0.893$, $p=0.372$).

Comparisons between the study participants and those who did not respond can be seen in Supplementary Table 1. In both mental and physical health study samples, there were no significant differences between responders and non-responders on gender or SRS score. In the physical health, but not the mental health study, responders were significantly older (mean age = 44.4) than non-responders (41.0) ($t(668)= 3.369$, $p=0.001$).

Considering mental and physical health study participants, key demographic characteristics are shown in Table 1. Both mental and physical health samples contained a broadly equal split of genders, with slightly more females in both. There was a large overlap in health conditions between the samples with 74% (n= 397) of the mental health sample reporting a physical health condition and 86% (n= 351) of the physical health sample reporting a mental health condition. Comparisons were made between key demographic characteristics of the two samples (participants who completed both studies were not included to avoid double counting and so breaking the assumption of independence). There was no significant difference in gender ($\chi^2(3)=2.493$, $p=0.477$) or SRS-2 score ($t(443)= -1.814$, $p=0.70$) between samples. The physical health sample were significantly older than the mental health sample ($t(500)= -4.526$, $p<0.001$).

For peer review only - http://bmjopen.bmj.com/site/about/guidelines.xhtml

Table 1: Comparison of the demographic characteristics of participants included in the mental health and physical health samples

Variable	Mental Health Sample (PAT-A study)	Physical Health Sample (IHOAP study)
**Total N**	537	407
**Gender N (%)**		
Male	234 (43.6)	167 (41.0)
Female	281 (52.3)	227 (55.8)
Other/rather not say	22 (4.1)	13 (3.2)
**Age: Mean (SD) [Range]**	41.3 (13.8) [18.0-77.0]	44.4 (13.4) [18.0-79.0]
**Ethnicity N (%)**		
White British	501 (93.3)	374 (91.9)
Asian	3 (0.6)	1 (0.2)
Black	2 (0.4)	3 (0.7)
Mixed	10 (1.9)	8 (2.0)
Other/rather not say	8 (1.5)	7 (1.7)
Not reported	13 (2.4)	14 (3.4)
**Highest education N (%)**		
Postgraduate degree	94 (17.5)	72 (17.7)
Bachelor's degree	135 (25.1)	97 (23.8)
Diploma of higher education	34 (6.3)	28 (6.9)
Certificate of higher education	18 (3.4)	17 (4.2)
A-level	88 (16.4)	76 (18.7)
GCSE	108 (20.1)	71 (17.4)
Basic skills	20 (3.7)	10 (2.5)
No formal qualifications	32 (6.0)	30 (7.4)
Other	8 (1.5)	6 (1.5)
**Employment status N (%)**		
Employed without support[a]	213 (39.7)	154 (37.8)
Employed with support	9 (1.7)	5 (1.2)

For peer review only

For peer review only - http://bmjopen.bmj.com/site/about/guidelines.xhtml

Volunteer	49 (9.1)	38 (9.3)
Unemployed	170 (31.7)	137 (33.7)
Retired	30 (5.6)	25 (6.1)
Other	58 (10.8)	38 (9.3)
No response	8 (1.5)	10 (2.5)
**ASD diagnosis N (%)**		
Formal diagnosis	443 (82.5)	341 (83.8)
Suspected/unsure/awaiting assessment	94 (17.5)	66 (16.2)
**Age at diagnosis: Mean (SD) [Range]**	35.8 (15.8) [3.0-73.0]	38.7 (14.8) [2.0-68.0]
**SRS score[b] by severity category N (%)**		
Normal	20 (4.2)	9 (2.5)
Mild	45 (9.4)	35 (9.7)
Moderate	172 (35.8)	115 (31.9)
Severe	244 (50.7)	201 (55.8)
Mean (SD)	110.5 (25.6)	115.1 (25.8)
**Support received N (%)**		
Home	152 (15.5)	115 (15.2)
Employment	50 (5.1)	36 (4.8)
Health	110 (11.2)	95 (12.6)
Finance	129 (13.2)	99 (13.1)
Social	75 (7.7)	57 (7.5)
Lifelong learning	67 (6.9)	47 (6.2)
Community	78 (8.0)	68 (9.0)
Organisation	73 (7.5)	55 (7.3)
Do not receive support	244 (24.9)	183 (24.2)
**Mental health condition N (%)**		
Diagnosis	-	351 (86.2)
No diagnosis or suspected	-	56 (13.8)
**Physical health condition N (%)**		
Diagnosis	397 (73.9)	-

For peer review only - http://bmjopen.bmj.com/site/about/guidelines.xhtml

For peer review only

No diagnosis or suspected	140 (26.1)	-

a- includes self-employed; b- not available for all participants; c- may add to more than total sample size as multiple responses were allowed

Abbreviations: SD = Standard Deviation, ASD = Autism Spectrum Disorder, SRS = Social Responsiveness Scale – 2nd Edition

286

287 **Service use**

288 73.4% (n= 394) of the participants who completed the anxiety survey had been prescribed
289 medication for anxiety and 76.5% (n= 400) had been referred for psychological therapies for
290 anxiety. Of those who were referred for psychological therapies, 80% (n= 320) completed
291 treatment, 16% (n= 63) stopped attending before treatment was considered complete and 4%
292 (n= 17) did not attend. Of those who attended at least one session of psychological therapy but
293 did not complete this, 64% (n= 40) cited a lack of adjustments to meet their needs as a
294 contributing factor. Within mental health services, 76% (n= 280) reported that they had not
295 been offered adjustments. Of these people, 69% (n= 193) reported that adjustments would have
296 been required or helpful.

297

298 92% (n= 375) of participants who completed the physical health survey had seen their GP
299 within the previous 12 months and 59% (n= 240) had seen a specialist or attended a specialist
300 clinic in the previous two years. 59% (n= 240) of respondents reported a medical condition that
301 required them to attend regular medical appointments. Of these people, 24% (n= 58) reported
302 missing at least one appointment. 45% (n= 26) reported issues related to the sensory
303 environment, mental health conditions (e.g. anxiety about attending appointments) and
304 dissatisfaction with healthcare settings (e.g. poor accessibility) as reasons for missing
305 appointments. Within physical health services, 80% (n= 304) reported that they had not been

For peer review only - http://bmjopen.bmj.com/site/about/guidelines.xhtml

offered adjustments. Of these people, 56% (n= 171) reported that adjustments would have been required or helpful.

Categorising key adjustments to services

Exploratory factor analysis (EFA) supported the retention of between two and four factors. Using an a-priori loading criterion of .4 a clear three-factor solution was retained in the mental health data (factor loadings of >.4 for 17/18 items; intercorrelations: .69 to .79). The factors related to adjustments for 1) 'sensory environment', 2) 'clinical and service context' and 3) 'clinician knowledge and communication'. The adjustment '*short waiting times to be seen when you attend appointments*' did not load clearly onto any factor. This solution demonstrated very good/excellent model fit across all tested indices. A four-factor solution was most interpretable for the physical health data (factor loadings of > .4 for 15/18 items and good model fit). Closer inspection indicated strong similarities across both solutions. Thirteen out of 18 adjustments loaded onto the same, or similar factors. Data for the item relating to shorter waiting times are presented alone (see Supplementary tables 2, 3 and 4 for factor loadings and model fit indices). The importance (Table 2) and availability (Table 3) of adjustments are presented in the categories identified by factor analysis. Full response frequencies for the importance of adjustments can be seen in Supplementary Table 5 and availability of adjustments in Supplementary Table 6.

For peer review only - http://bmjopen.bmj.com/site/about/guidelines.xhtml

Table 2. The importance of key adjustments to mental and physical health services to meet the needs of autistic adults

Key adjustment	Mental Health Services		Physical Health Services	
	Important [a] % (N)	Very important % (N)	Important [a] % (N)	Very important % (N)
**F1: Sensory Environment**				
Change the sensory environment in the building that the appointment will take place in	73.0 (387)	39.4 (209)	74.0 (289)	42.5 (166)
Locations (e.g. waiting rooms) with small numbers of people	81.3 (430)	50.7 (268)	82.2 (324)	52.8 (208)
Locations with low noise levels	89.1 (471)	60.9 (322)	88.3 (348)	61.9 (244)
Locations with low light levels	64.3 (338)	35.2 (185)	67.0 (259)	35.7 (138)
**F2: Clinical and Service Context**				
Changing the length of appointments to suit you	70.0 (375)	36.2 (194)	78.7 (311)	44.8 (177)
Offering appointments online or via apps	45.8 (240)	22.9 (120)	50.0 (194)	31.2 (121)
Changing how often you are asked to attend appointments	55.2 (293)	24.5 (130)	46.7 (178)	18.1 (69)
Give information to the clinician pre-appointment so that they can prepare	88.3 (469)	60.6 (322)	80.8 (320)	48.0 (190)
Provide support in relation to attending appointments (e.g. managing fears or uncertainties which might make attending difficult)	81.2 (428)	53.5 (282)	78.9 (307)	50.9 (198)
Appropriate distractions provided whilst waiting to be seen at appointment (e.g. tablet with headphones)	48.3 (253)	25.2 (132)	52.0 (199)	27.7 (106)
**F3: Clinician Knowledge and Communication**				
Clinicians who understand autism	98.3 (524)	87.6 (467)	96.5 (386)	80.5 (322)
Opportunity after the appointment to ask questions about conclusions	91.3 (487)	60.2 (321)	89.8 (353)	64.4 (253)
Appointments at an easily identified and accessible location	90.0 (478)	70.4 (374)	88.8 (351)	71.7 (281)
Appointments with an easily identified and familiar clinician	90.0 (477)	68.9 (365)	90.8 (357)	71.0 (279)
Having a health summary document which can be shared with clinicians (e.g. hospital passport)	79.9 (420)	51.0 (268)	74.8 (288)	48.8 (188)
A clinician who uses an approach which is informed by what you have said that you prefer (e.g. formal or informal)	91.8 (485)	60.2 (318)	80.6 (311)	50.0 (193)
Identifying reasons that make it difficult to see a clinician or attend an appointment	82.6 (434)	49.5 (260)	82.7 (316)	52.9 (202)
**Standalone item**				
Short waiting times to be seen when you attend appointments	86.7 (461)	55.1 (293)	86.0 (338)	57.8 (227)

For peer review only

a 'Important shows the number and percentage of participants who rated each adjustment as either 'somewhat important' or 'very important'

Most respondents reported that all adjustments were 'somewhat important' or 'very important' (hereafter referred to as important) in both mental and physical health services. In mental health services, those rating each adjustment as important ranged from 46% (n=240) (access to online appointments) to 98% (n= 525) (clinician who understands autism). In physical health services, these ranged from 47% (n= 178) (changing frequency of appointments) to 97% (n= 386) (clinician who understands autism). High proportions of participants also rated each adjustment as 'very important' ranging from 23% (n= 120) (access to online appointments) to 88% (n= 468) (clinician who understands autism) in mental health services and 18% (n= 69) (changing frequency of appointments) to 81% (n= 322) (clinician who understands autism) in physical health services. Autistic adults rated *'having access to a clinician who understands autism'* as the most important adjustment in both mental health (98.3%, n= 524) and physical health (96.5%, n= 386) settings.

For peer review only - http://bmjopen.bmj.com/site/about/guidelines.xhtml

373 **Table 3: The lack of availability of key adjustments to mental and physical healthcare**
374 **services to meet the needs of autistic adults**
375

**Key adjustment**	**Mental Health Services**		**Physical Health Services**	
	Rarely or never available % (N)	Never available % (N)	Rarely or never available % (N)	Never available % (N)
**Sensory Environment**				
Change the sensory environment in the building that the appointment will take place in	72.2 (171)	45.6 (108)	83.6 (199)	60.5 (144)
Locations (e.g. waiting rooms) with small numbers of people	51.6 (146)	29.3 (83)	76.1 (203)	49.1 (131)
Locations with low noise levels	52.0 (144)	26.0 (72)	69.4 (189)	42.6 (116)
Locations with low light levels	65.2 (161)	40.9 (101)	83.3 (205)	52.4 (129)
**Clinical and Service Context**				
Changing the length of appointments to suit you	68.4 (154)	45.3 (102)	70.7 (164)	41.4 (96)
Offering appointments online or via apps	64.6 (126)	48.2 (94)	50.2 (117)	32.6 (76)
Changing how often you are asked to attend appointments	53.0 (96)	27.6 (50)	67.3 (99)	44.2 (65)
Give information to the clinician pre-appointment so that they can prepare	56.1 (123)	33.3 (73)	66.4 (148)	41.3 (92)
Provide support in relation to attending appointments (e.g. managing fears or uncertainties which might make attending difficult)	62.1 (146)	40.4 (95)	76.6 (167)	45.9 (100)
Appropriate distractions provided whilst waiting to be seen at appointment (e.g. tablet with headphones)	79.0 (181)	63.3 (145)	84.6 (176)	66.3 (138)
**Clinician Knowledge and Communication**				
Clinicians who understand autism	71.8 (186)	31.3 (81)	73.0 (170)	36.9 (86)
Opportunity after the appointment to ask questions about conclusions	42.8 (113)	18.9 (50)	47.0 (121)	26.8 (69)
Appointments at an easily identified and accessible location	25.4 (78)	10.1 (31)	25.0 (93)	13.0 (38)
Appointments with an easily identified and familiar clinician	31.2 (89)	13.0 (37)	41.4 (120)	16.1 (47)
Having a health summary document which can be shared with clinicians (e.g. hospital passport)	69.8 (134)	52.1 (100)	81.5 (150)	63.6 (117)
A clinician who uses an approach which is informed by what you have said that you prefer (e.g. formal or informal)	51.4 (129)	23.5 (59)	62.9 (136)	42.1 (91)
Identifying reasons that make it difficult to see a clinician or attend an appointment	59.9 (127)	34.0 (72)	72.8 (144)	46.5 (92)
**Standalone item**				
Short waiting times to be seen when you attend appointments	52.3 (150)	27.2 (78)	69.6 (199)	35.7 (102)

For peer review only - http://bmjopen.bmj.com/site/about/guidelines.xhtml

For peer review only

For over half of respondents all but three adjustments were endorsed as ‘rarely or never available’ across both health service contexts. Adjustments were frequently endorsed as being ‘never available’ across both settings, ranging from 10% (n= 31) to 63% (n= 145) in mental health and 13% (n= 38) to 67% (n= 138) in physical health. In both cases, the adjustment most frequently rated as ‘never available’ was ‘being provided with appropriate distractions whilst waiting for appointments’ and the item least frequently rated as ‘never available’ was ‘appointments at an easily identified and familiar location’. A significant proportion of respondents to both surveys did not feel that they were able to comment on the availability of adjustments (range = mental health: 42.5% – 62.4%; physical health: 19.4% - 47.2%).

Other adjustments endorsed by autistic people

16% (n= 85) of participants in the mental health survey and 14% (n= 56) in the physical health survey gave information in open text about an ‘other’ adjustment; some of these were not coded (e.g. general comments). The majority (90% mental health, 91% physical health) of open text comments were classified within the 3-factor solution (see Table 4). An additional adjustment *“Being able to bring a supporter such as a friend, family member (or support animal) to appointments”* was suggested by several participants in both health service contexts.

Table 4. Reporting the number of open text comments that could be categorised to the identified factors following framework analysis

	Mental Health Services	Physical Health Services
**Sensory Environment**	8	5
**Clinical and Service Context**	33	24
**Clinician Knowledge & Communication**	16	10
**Other**	6	4

For peer review only - http://bmjopen.bmj.com/site/about/guidelines.xhtml

Paired comparison of the importance and availability of adjustments between physical and mental health services

221 participants completed both surveys. After Bonferroni correction, only one adjustment was rated as being very important significantly more often in mental health (65%) compared with physical health settings (47%): *'a clinician who uses an approach which is informed by what you have said that you prefer'* (p = .001). No significant differences in the least available adjustments based on service type were observed. Full reporting of these analyses can be found in Supplementary Tables 7 and 8.

Discussion

This large study successfully identified for the first time autistic adults' views about the importance and availability of key adjustments that UK and international guidelines and research consider could improve accessibility and acceptability of healthcare services. Autistic adults strongly endorsed the importance of these adjustments; the relative importance of each adjustment was broadly equivalent across physical and mental health service contexts. However, autistic people experienced limited availability to key adjustments across both healthcare contexts – showing clear unmet clinical need. Given that autistic people are at increased risk of combinations of mental and physical health conditions, healthcare providers need to take action now to implement provision of adjustments to improve service accessibility. Furthermore, this UK sample was large, recruited nationwide, and was demographically diverse; as the majority of respondents reported they experienced both mental health conditions and physical health disorders, it is likely that these findings can be generalised to other autistic adults in the UK. This suggestion is further supported by the finding that there were no significant differences between the key demographic variables of responders and non-responders to the survey, with the exception of age in the physical health study (see Supplementary Table 1). The availability and lack of availability of adjustments will however

For peer review only - http://bmjopen.bmj.com/site/about/guidelines.xhtml

vary, not only within the UK, but also in other countries and especially those without publicly funded healthcare.

Three distinct but inter-correlated factors of adjustments were identified: 1) the sensory environment, 2) the clinical and service context and 3) clinician knowledge and understanding. These factors are consistent with recent syntheses of the evidence regarding the barriers to healthcare access for autistic people (12, 13) and recent NHS reasonable adjustment guidance (26), which include adjustments under the categories of environment, service delivery, and communication. Our data-driven approach to categorisation is novel. The findings support and extend previous theoretical attempts at categorisation of adjustments to healthcare. The majority of 'other' adjustments proposed by participants could be classified within the three-factor model, providing further validation. Participants also identified across both service contexts the benefit of having the opportunity to attend appointments with support (a friend, family member). In common with all the adjustments included in our survey, this adjustment is not on the face of it difficult to implement, and it is likely most services would be supportive if asked. The NHS reasonable adjustments policy does already include having support at appointments amongst the potential adjustments offered (26) and recommends that such a request should be discussed and noted.

The adjustment rated as being most important in both settings was 'access to a clinician who understands autism'. Indeed, the four most important adjustments endorsed across both healthcare settings related to the 'clinician knowledge and communication' factor. This finding is in keeping with previous findings that healthcare professionals may lack skills and/or confidence in delivering interventions to autistic people (17). A recent review found that some autistic people feel reluctant to report their autism diagnosis to healthcare

For peer review only - http://bmjopen.bmj.com/site/about/guidelines.xhtml

professionals for fear that it may negatively affect their care (31). This suggests that care must be taken to establish an environment whereby the patient feels confident enough to discuss their autism and what adjustments would help them fully access healthcare services openly. When the priority adjustments were compared across the paired sample, only one adjustment '*A clinician who uses an approach which is informed by what you have said that you prefer (e.g. formal or informal)'* was significantly different between healthcare contexts. Perhaps there is a perception that mental healthcare clinicians need to take into account individual preference; however, coming to a shared understanding and approach to healthcare is critical in any clinical setting. The discrepancy between the rated importance of the identified adjustments and their reported availability in both physical and mental health services makes stark reading. Our findings highlight the wide 'gap' in the perceived availability of adjustments reported as important. Closer examination of our findings on the availability of adjustments allows us to highlight potential shortfalls in UK health services. The high percentages of 'rarely or never available' responses seen across many adjustments suggests a systematic shortfall across the NHS rather than variability between services. However, encouragingly two adjustments [*appointments at a familiar location* and with a *familiar clinician*] were rated as being available more than rarely by over half of each sample. Offering consistency in clinical care was appreciated in both contexts. In keeping with recently published findings identifying widespread barriers to healthcare access for autistic people (12, 13), respondents consistently rated their experiences of not being able to access adjustments in healthcare. If autistic adults are not experiencing these adjustments to promote accessibility, they are likely to continue to experience delays in mental and physical health diagnosis, poor treatment outcomes (20), significant inequalities and premature mortality (11).

For peer review only

Strengths and Limitations

Our study has many strengths. The research aimed to match published autism community research priorities and focus directly on the experiences of autistic people (18). The study also includes experiences about adjustments in both mental and physical healthcare settings. Our large samples were both approximately equal in terms of male and female participants, which is a strength considering the low numbers of female participants often observed in autism research. Our data driven approach enabled us to determine key overarching factors or categories of adjustments that could be used to inform both policy and practice in relation to service development.

One limitation of our study is that some participants reported that they did not have sufficient knowledge to comment on the availability of adjustments and were not able to provide a rating. However, the use of paired comparisons provides more statistical power than between group analyses by virtue of eliminating the potential for sources of between-person variance (32). Further, that a notable proportion of the sample reported they were not informed of the availability of adjustments they might want, is an important finding in itself. Our data are based on the reported experiences of the respondents. We did not aim to collect information directly from the services, and this should be done in future studies. However, the samples are large and recruited from diverse sources across the UK including the NHS, private and charity providers, so it is unlikely systematic bias was introduced. The participants in the mental health sample were all individuals who had self-reported either a diagnosed or suspected anxiety condition (relating to the aims of the original study). This may have the potential to limit the generalisability of the findings for other mental health settings – although many mental health services have a similar service design, and clinicians tend to deliver care for a variety of mental health conditions.

For peer review only - http://bmjopen.bmj.com/site/about/guidelines.xhtml

Implications and future research

The findings of this study highlight the lack of key adjustments available to autistic adults currently accessing physical and mental healthcare. These adjustments do not require expensive technology or equipment but rather ongoing staff training, attention to aspects of the clinical settings and flexibility in ways of delivering clinical care. We consider that 'how' and 'what' adjustments can be made should be more explicit in pre-appointment information, and during appointments. Focussing on the sensory environment, clinical and service context, clinician knowledge and communication and involvement of supporters, may help service users, providers and commissioners of services to identify and address any barriers to implementation. Proactively considering these adjustments with autistic people is in our opinion a prudent and logical first step towards minimising the barriers to healthcare. This in turn is a crucial component of an individualised /personalised approach to assessment and management of identified healthcare needs in order to maximise treatment effectiveness and reduce health inequalities. Our current research is developing and evaluating personalised mental and physical healthcare provision for autistic people across the lifespan. Examples include the co-design (with autistic people, relatives and clinicians) and evaluation of a personalised, flexible psychological intervention for anxiety in autistic adults (PAT-A, 33). We have also co-designed an intervention to investigate how autistic people age 50 and over can be encouraged to access the healthcare they need (Trial registration: ISRCTN75726745). To address the primary care needs of autistic adults, we have co-designed and are evaluating the use of primary care healthchecks for autistic people (https://research.ncl.ac.uk/autismhealthchecks/). All these approaches systematically gather information about the adjustments required to maximise the chances of success (details of the tools used to do this available on request). Co-designed approaches to healthcare that meets the

For peer review only - http://bmjopen.bmj.com/site/about/guidelines.xhtml

needs of autistic people and clinicians should increase the opportunities for autistic people to access the personalised and precision health care that is increasingly available to all people in mental and physical health services. In turn, that will hopefully reduce the increased and premature morbidity and average earlier mortality for autistic people.

Author Details

Samuel Brice[1], Jacqui Rodgers[1], Barry Ingham[1,2], David Mason[1], Colin Wilson[1], Mark Freeston[3], Ann Le Couteur[1] & Jeremy R. Parr[1,2]

1. Population Health Sciences Institute, Newcastle University, UK
2. Cumbria, Northumberland, Tyne and Wear NHS Foundation Trust, UK
3. School of Psychology, Newcastle University, UK

Corresponding Author

Professor Jeremy R Parr

Email: Jeremy.Parr@ncl.ac.uk

Address: Level 3, Sir James Spence Institute, Royal Victoria Infirmary, Queen Victoria Road, Newcastle upon Tyne, NE1 4LP

Author contributions

JP, BI and JR conceptualised the adjustments study. JP and JR received funding for PAT-A. BI and JP received funding for IHOAP. JP and BI liaised with NHSE about adjustments. JR and MF supervised statistical analyses. SB and DM are researchers for PAT-A and IHOAP respectively and led data collection and undertook analysis. CW, MF and ALC provided comment on PAT-A and adjustment study design and analyses. All authors contributed to adjustments study design, and writing of the manuscript, and reviewed the final version.

For peer review only - http://bmjopen.bmj.com/site/about/guidelines.xhtml

Declarations of Interest

MF reports personal fees from Honoraria from training organisations in the area of psychological therapies, personal fees from Royalties from books in the area of psychological therapies, outside of the submitted work. All other authors declare no conflicts of interest.

Acknowledgements

We are grateful to: all the participants who gave their time to complete the PAT-A and IHOAP surveys; the IHOAP investigators; the ASC-UK team including Dr Alex Petrou (who supported data collection), and the ASC-UK investigators, including Professor Helen McConachie. The Sponsor for the studies was Cumbria, Northumberland, Tyne and Wear NHS Foundation Trust.

Funding

Data from autistic adults were collected as part of two studies:

1. Personalised Anxiety Treatment for Autistic Adults study funded by the UK autism research charity Autistica. Funding was awarded to JP and JR as part of the autism spectrum adulthood and ageing research programme at Newcastle University (Grant ID: 7425).

2. Improving the Health of Older Autistic People study, funded by the Inge Wakehurst Trust, awarded to BI and JP (no grant number given)

Data Availability

Some data that support the findings of this study are available on reasonable request from the corresponding author [JP]. The data are not publicly available due to containing information that could compromise the privacy of research participants.

For peer review only

For peer review only - http://bmjopen.bmj.com/site/about/guidelines.xhtml

License Statement

I, the Submitting Author has the right to grant and does grant on behalf of all authors of the Work (as defined in the below author licence), an exclusive licence and/or a non-exclusive licence for contributions from authors who are: i) UK Crown employees; ii) where BMJ has agreed a CC-BY licence shall apply, and/or iii) in accordance with the terms applicable for US Federal Government officers or employees acting as part of their official duties; on a worldwide, perpetual, irrevocable, royalty-free basis to BMJ Publishing Group Ltd ("BMJ") its licensees and where the relevant Journal is co-owned by BMJ to the co-owners of the Journal, to publish the Work in BMJ Open and any other BMJ products and to exploit all rights, as set out in our licence.

The Submitting Author accepts and understands that any supply made under these terms is made by BMJ to the Submitting Author unless you are acting as an employee on behalf of your employer or a postgraduate student of an affiliated institution which is paying any applicable article publishing charge ("APC") for Open Access articles. Where the Submitting Author wishes to make the Work available on an Open Access basis (and intends to pay the relevant APC), the terms of reuse of such Open Access shall be governed by a Creative Commons licence – details of these licences and which Creative Commons licence will apply to this Work are set out in our licence referred to above.

References

1. American Psychiatric Association. Diagnostic and Statistical Manual of mental disorders (5th ed.). . Washington: APA; 2013.
2. Brugha TS, McManus S, Bankart J, Scott F, Purdon S, Smith J, et al. Epidemiology of autism spectrum disorders in adults in the community in England. Arch Gen Psychiatry. 2011;68(5):459-65.

For peer review only - http://bmjopen.bmj.com/site/about/guidelines.xhtml

3. Rydzewska E, Hughes-McCormack LA, Gillberg C, Henderson A, MacIntyre C, Rintoul J, et al. Prevalence of long-term health conditions in adults with autism: observational study of a whole country population. BMJ Open. 2018;8(8):e023945.
4. Hand BN, Angell AM, Harris L, Carpenter LA. Prevalence of physical and mental health conditions in Medicare-enrolled, autistic older adults. Autism. 2019;24(3):755-64.
5. Lawson LP, Richdale AL, Haschek A, Flower RL, Vartuli J, Arnold SR, et al. Cross-sectional and longitudinal predictors of quality of life in autistic individuals from adolescence to adulthood: The role of mental health and sleep quality. Autism. 2020;24(4):954-67.
6. Mason D, McConachie H, Garland D, Petrou A, Rodgers J, Parr JR. Predictors of quality of life for autistic adults. Autism Res. 2018;11(8):1138-47.
7. Croen LA, Zerbo O, Qian Y, Massolo ML, Rich S, Sidney S, et al. The health status of adults on the autism spectrum. Autism. 2015;19(7):814-23.
8. Hollocks MJ, Lerh JW, Magiati I, Meiser-Stedman R, Brugha TS. Anxiety and depression in adults with autism spectrum disorder: a systematic review and meta-analysis. Psychol Med. 2019;49(4):559-72.
9. Hudson CC, Hall L, Harkness KL. Prevalence of Depressive Disorders in Individuals with Autism Spectrum Disorder: a Meta-Analysis. J Abnorm Child Psychol. 2019;47(1):165-75.
10. Trembath D, Germano C, Johanson G, Dissanayake C. The Experience of Anxiety in Young Adults With Autism Spectrum Disorders. Focus on Autism and Other Developmental Disabilities. 2012;27(4):213-24.
11. Hirvikoski T, Mittendorfer-Rutz E, Boman M, Larsson H, Lichtenstein P, Bölte S. Premature mortality in autism spectrum disorder. British Journal of Psychiatry. 2016;208(3):232-8.
12. Bradshaw P, Pellicano E, van Driel M, Urbanowicz A. How Can We Support the Healthcare Needs of Autistic Adults Without Intellectual Disability? Current Developmental Disorders Reports. 2019;6(2):45-56.
13. Mason D, Ingham B, Urbanowicz A, Michael C, Birtles H, Woodbury-Smith M, et al. A Systematic Review of What Barriers and Facilitators Prevent and Enable Physical Healthcare Services Access for Autistic Adults. Journal of Autism and Developmental Disorders. 2019;49(8):3387-400.
14. Doherty M, Sullivan JD, Neilson SD. Barriers to healthcare for autistic adults: Consequences & policy implications. A cross-sectional study. medRxiv. 2020:2020.04.01.20050336.
15. Maisel ME, Stephenson KG, South M, Rodgers J, Freeston MH, Gaigg SB. Modeling the cognitive mechanisms linking autism symptoms and anxiety in adults. J Abnorm Psychol. 2016;125(5):692-703.
16. Nicolaidis C, Raymaker DM, Ashkenazy E, McDonald KE, Dern S, Baggs AE, et al. "Respect the way I need to communicate with you": Healthcare experiences of adults on the autism spectrum. Autism : the international journal of research and practice. 2015;19(7):824-31.
17. McCormack G, Dillon AC, Healy O, Walsh C, Lydon S. Primary Care Physicians' Knowledge of Autism and Evidence-Based Interventions for Autism: A Systematic Review. Review Journal of Autism and Developmental Disorders. 2019.
18. James Lind Alliance. Autism Top Ten 2016 [Available from: http://www.jla.nihr.ac.uk/priority-setting-partnerships/autism/top-10-priorities/.
19. Crane L, Adams F, Harper G, Welch J, Pellicano E. 'Something needs to change': Mental health experiences of young autistic adults in England. Autism. 2018:1362361318757048.

For peer review only

For peer review only - http://bmjopen.bmj.com/site/about/guidelines.xhtml

20. Nicolaidis C, Raymaker D, McDonald K, Dern S, Boisclair WC, Ashkenazy E, et al. Comparison of healthcare experiences in autistic and non-autistic adults: a cross-sectional online survey facilitated by an academic-community partnership. J Gen Intern Med. 2013;28(6):761-9.
21. National Development Team for Inclusion. Green Light Toolkit: A Guide to Auditing and Improving your Mental Health Service so that it is Effective in Supporting People with Autism and People with Learning Disabilities. 2017 [Available from: https://www.ndti.org.uk/uploads/files/Green_Light_Toolkit_2017.pdf.
22. National Institute for Health and Care Excellence. Autism spectrum disorder in adults: diagnosis and management. Clinical guideline [CG142]. 2012.
23. NHS England. The NHS Long Term Plan 2019 [Available from: https://www.longtermplan.nhs.uk/wp-content/uploads/2019/08/nhs-long-term-plan-version-1.2.pdf.
24. Gruber JNCCP. Social Responsiveness Scale–Second Edition (SRS-2). Torrance, CA: Western Psychological Services.; 2012.
25. Qualtrics. Qualtrics. Provo, Utah, USA2005.
26. NHS Digital. Reasonable Adjustment Flag [Available from: https://digital.nhs.uk/services/reasonable-adjustment-flag.
27. Mandell DS, Lawer LJ, Branch K, Brodkin ES, Healey K, Witalec R, et al. Prevalence and correlates of autism in a state psychiatric hospital. Autism. 2012;16(6):557-67.
28. Lorenzo-Seva U, Ferrando PJ. FACTOR 9.2: A Comprehensive Program for Fitting Exploratory and Semiconfirmatory Factor Analysis and IRT Models. Applied Psychological Measurement. 2013;37(6):497-8.
29. Srivastava A, Thomson SB. Framework analysis: a qualitative methodology for applied policy research. Journal of Administration and Governance. 2009;72.
30. Simes RJ. An Improved Bonferroni Procedure for Multiple Tests of Significance. Biometrika. 1986;73(3):751-4.
31. Walsh C, Lydon S, O'Dowd E, O'Connor P. Barriers to Healthcare for Persons with Autism: A Systematic Review of the Literature and Development of A Taxonomy. Developmental Neurorehabilitation. 2020:1-18.
32. Wacholder S, Weinberg CR. Paired versus Two-Sample Design for a Clinical Trial of Treatments with Dichotomous Outcome: Power Considerations. Biometrics. 1982;38(3):801-12.
33. Parr JR, Brice S, Welsh P, Ingham B, Le Couteur A, Evans G, et al. Treating anxiety in autistic adults: study protocol for the Personalised Anxiety Treatment–Autism (PAT-A©) pilot randomised controlled feasibility trial. Trials. 2020;21(1):265.

For peer review only

For peer review only - http://bmjopen.bmj.com/site/about/guidelines.xhtml

Supplementary Information

Supplementary Table 1: Demographic information for the mental health and physical health samples and comparison with non-responders

	Mental Health (PAT-A)		Physical Health (IHOAP)	
Variable	Responders	Non-responders	Responders	Non-responders
Total N	537	545	407	484
Gender N (%)				
Male	234 (43.6)	273 (50.1)	167 (41.0)	238 (49.1)
Female	281 (52.3)	254 (46.6)	227 (55.8)	236 (48.8)
Other/rather not say	22 (4.1)	18 (3.3)	13 (3.2)	10 (2.1)
Age: Mean (SD) [Range]	41.3 (13.8) [17.0-77.0]	37.6 (12.9) [17.0-84.0]	44.4 (13.4) [18.0-79.0]	41.0 (13.3) [18.0-90.0]
Ethnicity				
White	501 (93.3)	496 (91.0)	374 (91.9)	437 (90.3)
Asian	3 (0.6)	8 (1.5)	1 (0.2)	6 (1.2)
Black	2 (0.4)	3 (0.6)	3 (0.7)	2 (0.4)
Mixed	10 (1.9)	10 (1.8)	8 (2.0)	11 (2.3)
Other/rather not say	8 (1.5)	4 (0.7)	7 (1.7)	6 (1.2)
Not reported	13 (2.4)	24 (4.4)	14 (3.4)	26 (5.4)
Highest education				
Postgraduate degree	94 (17.5)	67 (12.3)	72 (17.7)	69 (14.3)
Bachelor's degree	135 (25.1)	122 (22.4)	97 (23.8)	97 (20.0)
Diploma of higher education	34 (6.3)	43 (7.9)	28 (6.9)	52 (10.7)
Certificate of higher education	18 (3.4)	27 (5.0)	17 (4.2)	19 (3.9)
A-level	88 (16.4)	115 (21.1)	76 (18.7)	93 (19.2)
GCSE	108 (20.1)	86 (15.8)	71 (17.4)	85 (17.6)
Basic skills	20 (3.7)	27 (5.0)	10 (2.5)	24 (5.0)
No formal qualifications	32 (6.0)	34 (6.2)	30 (7.4)	36 (7.4)
Other	8 (1.5)	24 (4.4)	6 (1.5)	9 (1.9)
Employment status N (%)				
Employed without support[a]	213 (39.7)	204 (37.4)	154 (37.8)	163 (33.7)
Employed with support	9 (1.7)	8 (1.5)	5 (1.2)	10 (2.1)
Volunteer	49 (9.1)	55 (10.1)	38 (9.3)	10 (2.1)
Unemployed	170 (31.7)	188 (34.5)	137 (33.7)	202 (41.7)
Retired	30 (5.6)	20 (3.7)	25 (6.1)	24 (5.0)
Other	58 (10.8)	57 (10.5)	38 (9.3)	62 (12.8)
No response	8 (1.5)	13 (2.4)	10 (2.5)	13 (2.7)
ASD diagnosis N (%)				
Formal diagnosis	443 (82.5)[d]	467 (85.7)	341 (83.8)[e]	415 (85.7)
Suspected/unsure/awaiting diagnosis	94 (17.5)[d]	78 (14.3)	66 (16.2)[e]	69 (14.3)
Age at diagnosis: Mean (SD) [Range]	35.8 (15.8) [3.0-73.0]	30.1 (15.5) [2.0-69.0]	38.7 (14.8) [2.0-68.0]	34.4 (15.8) [2.0-86.0]
SRS severity N (%)				
Normal	20 (4.2)	27 (7.2)	9 (2.5)	19 (5.7)
Mild	45 (9.4)	35 (9.3)	35 (9.7)	31 (9.2)
Moderate	172 (35.8)	112 (29.8)	115 (31.9)	97 (28.9)
Severe	244 (50.7)	202 (53.7)	201 (55.8)	189 (56.3)
SRS Mean (SD)	110.5 (25.6)	112.9 (29.2)	115.1 (25.8)	115.6 (28.2)
Support received[c] N (%)				
Home	152 (15.5)	162 (13.6)	115 (15.2)	142 (17.4)
Employment	50 (5.1)	78 (6.5)	36 (4.8)	53 (6.5)
Health	110 (11.2)	153 (12.8)	95 (12.6)	130 (16.0)
Finance	129 (13.2)	154 (12.9)	99 (13.1)	137 (16.8)
Social	75 (7.7)	115 (9.6)	57 (7.5)	88 (10.8)
Lifelong learning	67 (6.9)	75 (6.3)	47 (6.2)	60 (7.4)

For peer review only

Community	78 (8.0)	110 (9.2)	68 (9.0)	79 (9.7)
Organisation	73 (7.5)	110 (9.2)	55 (7.3)	93 (11.4)
Do not receive support	244 (24.9)	236 (19.8)	183 (24.2)	32 (3.9)
Mental health condition N (%)				
Diagnosis	-	-	351 (86.2)	414 (85.5)
No diagnosis	-	-	56 (13.8)	70 (14.5)
Physical health condition N (%)				
Diagnosis	397 (73.9)	402 (73.8)	-	-
No diagnosis	140 (26.1)	143 (26.2)	-	-
Response type				
Electronic	415 (77.3%	446 (81.8)	305 (74.9)	396 (81.8)
Paper	122 (22.7%)	99 (18.2)	102 (25.1)	88 (18.2)

Tests of difference between responders and non-responders on key demographic variables

Significant differences are denoted with *

Age:
Mental health sample: $t(859)$= 1.655, p=0.949
Physical health sample: $t(668)$= 3.369, p=0.001*

Gender:
Mental health sample: M,F,O,R genders Mental health sample vs Physical health sample $\chi^2(3)$=2.049, p=0.562; males and females only: $\chi^2(1)$=0.349, p=0.555
Physical health sample: M,F,O,R genders Mental health sample vs Physical health sample $\chi^2(3)$=4.481, p=0.214; males and females only: $\chi^2(1)$=1.007, p=0.316

Autism Characteristics (SRS total):
Mental health sample: $t(641.5)$= -1.135, p=0.257
Physical health sample: $t(496)$= -0.198, p=0.843

For peer review only

Exploratory Factor Analysis on participants' importance of adjustment ratings

Exploratory factor analysis (EFA) was conducted in order to assess whether the adjustments clustered together in a meaningful way. FACTOR software version 10.8.04 (1) was used to conduct EFA on data on the importance of each adjustment from both datasets from study 1 and study 2. Polychoric correlations were used as this is deemed most appropriate for use with ordinal and/or skewed data such as these (2). Unweighted least squares were used to extract the factors and the rotation method was Promin. The use of an oblique method of rotation such as this allows for the correlation between factors to be calculated, which is in line with best scientific practice (3). Bias correlated bootstrap resampling was undertaken using 5000 bootstrap samples. This decision was taken in order to reduce the influence of any outliers and maximise the generalisability of the sample. Model fit was assessed using a number of indices including Root Mean Square Error of Approximation (RMSEA), Non-Normed Fit Index (NNFI), Comparative Fit Index (CFI), Goodness of Fit Index (GFI) and Root Mean Square of Residuals (RMSR). Cut-off values used to demonstrate appropriate model fit can be seen after Suplementary Table 4.

Participants with at least one missing response on any of the importance of adjustment ratings were deleted listwise prior to conducting EFA as any efforts to impute missing items may have biased the results at this stage. This resulted in the removal of 51 participants from the mental health dataset, leaving a sample of N = 486 and 78 participants from the physical health dataset leaving a sample of N = 329. Sample size suitability for EFA has been a matter of debate, however best practice guidelines suggests that a participant-to-item ratio of between 5:1 and 10:1 is considered to be suitable (4). Therefore, the 27:1 and 18.3/1 participant-to-item ratios in the mental health and physical health datasets respectively were deemed more than adequate for EFA. Furthermore, the Kaiser-Meyer-Olkin (KMO) test revealed that the sampling

For peer review only

adequacy was 'very good' in both datasets (mental health: KMO = .92; physical health: KMO = .91).

Exploratory factor analysis (EFA) was conducted using data from participants' ratings of the importance of each key adjustment identified in the survey EFA was conducted separately for each survey (i.e. mental health and physical health). Decisions regarding the number of factors to retain was based on best practice guidelines (4), which included consulting existing theoretical knowledge, examining the scree plots, retaining factors with an eigenvalue greater than one and conducting parallel analysis. These indicators supported the retention of between two and four factors in each of the datasets. We applied an a-priori loading criterion of ≥ .4 to the rotated loading matrices, in order to ensure that emergent factors were meaningful, whilst not being so stringent as to limit the reproducibility of the findings (5)

References

1. Lorenzo-Seva U, Ferrando PJ. FACTOR 9.2: A Comprehensive Program for Fitting Exploratory and Semiconfirmatory Factor Analysis and IRT Models. Applied Psychological Measurement. 2013;37(6):497-8.
2. Baglin J. Improving your exploratory factor analysis for ordinal data: a demonstration using FACTOR. Practical Assessment, Research and Evaluation. 2014;19.
3. Osborne JW. What is Rotating in Exploratory Factor Analysis. Practical Assessment, Research and Evaluation. 2015;20.
4. Osborne JW. Best Practices in Exploratory Factor Analysis: Jason W. Osbourne; 2014.
5. Hoyle R, Duvall J. Determining the number of factors in exploratory and confirmatory factor analysis. 2004.

For peer review only

Supplementary Table 2: Factor loadings for the three-factor solution for importance of adjustments to mental health services and four-factor solution for the importance of adjustments to physical health services

Adjustment		Mental Health			Physical Health			
	Factor	F1	F2	F3	F1	F2	F3	F4
	Factor Code	SE	SC	CKC	SE	SC	CKC	F
9. Change the sensory environment in the building that the appointment will take place in		**0.799**	0.064	0.006	**0.925**	-0.178	0.120	0.015
10. Locations (e.g. waiting rooms) with small numbers of people		**0.787**	-0.001	0.081	**0.621**	0.258	-0.212	0.245
11. Locations with low noise levels		**1.065**	-0.186	-0.054	**0.905**	-0.094	-0.051	0.064
12. Locations with low light levels		**0.718**	0.099	-0.055	**0.874**	0.109	0.076	-0.230
2. Changing the length of appointments to suit you		-0.100	**0.551**	0.212	-0.098	0.222	0.376	0.284
3. Offering appointments online or via apps		-0.164	**0.765**	-0.222	-0.002	**0.502**	0.009	-0.123
4. Changing how often you are asked to attend appointments		-0.062	**0.685**	-0.038	-0.145	**0.829**	0.063	-0.094
5. Give information to the clinician pre-appointment so that they can prepare		-0.159	**0.417**	0.378	-0.070	0.119	**0.671**	0.041
17. Provide support in relation to attending appointments (e.g. managing fears or uncertainties which might make attending difficult)		0.117	**0.511**	0.184	0.115	0.294	0.087	0.378
18. Appropriate distractions provided whilst waiting to be seen at appointment (e.g. tablet with headphones)		0.215	**0.680**	-0.162	0.292	**0.422**	0.001	0.039
1. Therapists who understand autism		0.047	-0.458	**0.970**	0.274	-0.044	0.282	0.202
6. Opportunity after the appointment to ask questions about conclusions		-0.244	-0.162	**0.838**	-0.137	0.010	**0.836**	-0.042
7. Appointments at an easily identified and accessible location		0.063	-0.057	**0.625**	0.026	-0.041	0.043	**0.668**
8. Appointments with an easily identified and familiar clinician		0.124	-0.110	**0.685**	-0.036	-0.253	0.026	**1.044**
13. Having a health summary document which can be shared with clinicians (e.g. hospital passport)		-0.019	0.168	**0.556**	0.143	-0.163	**0.861**	-0.188
14. A clinician who uses an approach which is informed by what you have said that you prefer (e.g. formal or informal)		0.035	0.016	**0.624**	0.066	-0.135	**0.736**	0.103
15. Identifying reasons that make it difficult to see a clinician or attend an appointment		0.096	0.193	**0.474**	0.053	0.230	**0.495**	0.041
16. Short waiting times to be seen when you attend appointments		0.268	0.169	0.295	0.002	**0.410**	-0.028	**0.421**

For peer review only

Key: SS = Sensory Environment, SC = Clinical and Service Context, CKC = Clinician Knowledge and Communication, F = Familiarity
Factor loadings that exceed the ≥ 0.4 loading criteria are denoted in bold

Supplementary Table 3: Inter-factors correlation matrices

	Mental Health			Physical Health			
	**F1**	**F2**	**F3**	**F1**	**F2**	**F3**	**F4**
**F1**	1.000			1.000			
**F2**	0.688	1.000		0.722	1.000		
**F3**	0.732	0.785	1.000	0.595	0.727	1.000	
**F4**				0.694	0.705	0.736	1.000

Supplementary Table 4: Model fit indices

Model fit statistic	Mental Health	Physical Health
**Root Mean Square Error of Approximation (RMSEA)**	0.032	0.032
**Non-Normed Fit Index (NNFI)**	0.994	0.995
**Comparative Fit Index (CFI)**	0.996	0.997
**Goodness of Fit Index (GFI)**	0.993	0.995
**Root Mean Square of Residuals (RMSR)**	0.0384	0.0361

The following cut-off values were used to determine acceptable goodness of fit:

RMSEA < 0.08
NNFI ≥ 0.95
CFI ≥ 0.90
GFI ≥ 0.95
RMSR < 0.08.

For peer review only

Supplementary Table 5: Response frequencies for the importance of adjustments in both samples

Adjustment	Mental Health Services Importance Response Frequencies (N)						Physical Health Services Importance Response Frequencies (N)					
	1 Low	2	3	4	5 High	M	1 Low	2	3	4	5 High	M
Clinicians who understand autism	1	0	8	57	468	3	2	4	8	64	322	7
Changing the length of appointments to suit you	19	40	102	181	194	1	12	20	52	135	177	11
Offering appointments online or via apps	89	66	128	120	120	14	65	45	84	73	121	19
Changing how often you are asked to attend appointments	32	33	173	163	130	6	51	42	110	109	69	26
Give information to the clinician pre-appointment so that they can prepare	11	17	34	147	322	6	20	13	43	130	190	11
Opportunity after the appointment to ask questions about conclusions	5	10	31	166	321	4	8	5	27	100	253	14
Appointments at an easily identified and accessible location	5	5	43	104	374	6	6	8	30	70	281	12
Appointments with an easily identified and familiar clinician	8	11	34	112	365	7	9	9	18	78	279	14
Change the sensory environment in the building that the appointment will take place in	32	28	83	178	209	7	23	23	56	123	166	16
Locations (e.g. waiting rooms) with small numbers of people	19	25	55	162	268	8	17	14	39	116	208	13
Locations with low noise levels	10	16	32	149	322	8	11	14	21	104	244	13
Locations with low light levels	43	42	103	153	185	11	20	27	81	121	138	20
Having a health summary document which can be shared with clinicians (e.g. hospital passport)	16	17	73	152	268	11	13	15	69	100	188	22
A clinician who uses an approach which is informed by what you have said that you prefer (e.g. formal or informal)	6	6	31	167	318	9	7	15	53	117	193	22
Identifying reasons that make it difficult to see a clinician or attend an appointment	13	8	70	174	260	12	10	8	48	114	202	25
Short waiting times to be seen when you attend appointments	7	20	44	168	293	5	7	14	34	111	227	14
Provide support in relation to attending appointments (e.g. managing fears or uncertainties which might make attending difficult)	20	21	58	146	282	10	18	14	50	109	198	18
Appropriate distractions provided whilst waiting to be seen at appointment (e.g. tablet with headphones)	62	66	142	121	132	14	51	31	101	93	106	25

Response option key: 1 = not at all important, 2 = not very important, 3 = neither important or unimportant, 4 = somewhat important, 5 = very important, M = missing response

For peer review only

Supplementary Table 6: Response frequencies for the availability of adjustments in both samples

Adjustment	Mental Health Services Availability Response Frequencies (N)							Physical Health Services Availability Response Frequencies (N)						
	1 Low	2	3	4	5 High	DK	M	1 Low	2	3	4	5 High	DK	M
Therapists who understand autism	81	105	32	27	14	261	17	86	84	30	23	10	146	28
Changing the length of appointments to suit you	102	52	29	32	10	292	20	96	68	25	27	16	134	41
Offering appointments online or via apps	94	32	25	26	18	321	21	76	41	35	36	45	125	49
Changing how often you are asked to attend appointments	50	46	32	37	16	335	21	65	34	21	19	8	192	68
Give information to the clinician pre-appointment so that they can prepare	73	50	31	42	23	301	17	92	56	25	29	21	139	45
Opportunity after the appointment to ask questions about conclusions	50	63	32	73	46	255	18	69	52	43	65	28	113	37
Appointments at an easily identified and accessible location	31	47	62	103	64	209	21	38	35	52	94	73	79	36
Appointments with an easily identified and familiar clinician	37	52	50	90	56	232	20	47	73	81	66	25	79	36
Change the sensory environment in the building that the appointment will take place in	108	63	26	27	13	279	21	114	55	16	14	9	124	45
Locations (e.g. waiting rooms) with small numbers of people	83	63	57	54	26	235	19	131	72	31	26	7	100	40
Locations with low noise levels	72	72	51	47	35	237	23	116	73	32	36	15	98	37
Locations with low light levels	101	60	31	36	19	261	29	129	76	12	23	6	111	50
Having a health summary document which can be shared with clinicians (e.g. hospital passport)	100	34	18	18	22	320	25	117	33	7	13	14	174	49
A clinician who uses an approach which is informed by what you have said that you prefer (e.g. formal or informal)	59	70	44	52	26	265	21	91	45	35	28	16	147	45
Identifying reasons that make it difficult to see a clinician or attend an appointment	72	55	29	37	19	300	25	92	52	23	20	11	152	57
Short waiting times to be seen when you attend appointments	78	72	43	69	25	228	22	102	97	50	29	8	79	42
Provide support in relation to attending appointments (e.g. managing fears or	95	51	32	35	22	280	22	100	67	24	12	15	136	43

For peer review only

uncertainties which might make attending difficult)														
Appropriate distractions provided whilst waiting to be seen at appointment (e.g. tablet with headphones)	145	36	14	21	13	281	27	138	38	10	9	13	137	62

Response option key: 1 = never available, 2 = rarely available, 3 = available about half the time, 4 = available most of the time, 5 = available all the time, DK = I don't know, M = missing response

For peer review only

Supplementary Table 7: McNemar tests showing a comparison of the adjustments that were most important to autistic people in mental and physical health services in a paired sample

Adjustment	N	Mental Health % very important	Physical Health % very important	P
Clinician who understands autism	217	87.6	80.2	.048
Changing the length of appointments to suit you	217	39.6	46.1	.211
Offering appointments online or via apps	206	23.8	28.6	.332
Changing how often you are asked to attend appointments	204	23.0	20.1	.526
Give information to the clinician pre-appointment so that they can prepare	214	64.5	49.5	.004
Opportunity after the appointment to ask questions about conclusions	214	62.6	64.0	.845
Appointments at an easily identified and accessible location	213	74.6	71.4	.520
Appointments with an easily identified and familiar clinician	211	69.7	72.0	.668
Change the sensory environment in the building that the appointment will take place in	210	40.0	44.8	.378
Locations (e.g. waiting rooms) with small numbers of people	213	50.2	51.2	.925
Locations with low noise levels	212	57.5	58.5	.924
Locations with low light levels	208	31.3	36.1	.373
Having a health summary document which can be shared with clinicians (e.g. hospital passport)	207	51.7	47.8	.497
A clinician who uses an approach which is informed by what you have said that you prefer (e.g. formal or informal)	206	64.6	46.6	**.001**
Identifying reasons that make it difficult to see a clinician or attend an appointment	202	47.0	51.5	.452
Short waiting times to be seen when you attend appointments	213	55.4	56.8	.848
Provide support in relation to attending appointments (e.g. managing fears or uncertainties which might make attending difficult)	208	55.8	50.0	.299
Appropriate distractions provided whilst waiting to be seen at appointment (e.g. tablet with headphones)	203	24.1	27.1	.572

* Significant P values following Simes adapted Bonferroni correction are highlighted in bold

For peer review only

Supplementary Table 8: McNemar tests showing a comparison of the adjustments that were least available to autistic people in mental and physical health services in a paired sample

Adjustment	N	Mental Health % never available	Physical Health % never available	P
Clinician who understands autism	65	29.2	40.0	.230
Changing the length of appointments to suit you	54	53.7	40.7	.296
Offering appointments online or via apps	43	46.5	34.9	.424
Changing how often you are asked to attend appointments	21	23.8	38.1	.549
Give information to the clinician pre-appointment so that they can prepare	54	35.2	38.9	.864
Opportunity after the appointment to ask questions about conclusions	72	20.8	25.0	.690
Appointments at an easily identified and accessible location	92	15.2	10.9	.503
Appointments with an easily identified and familiar clinician	82	14.6	17.1	.832
Change the sensory environment in the building that the appointment will take place in	51	60.8	51.0	.383
Locations (e.g. waiting rooms) with small numbers of people	75	32.0	49.3	.060
Locations with low noise levels	71	29.6	33.8	.711
Locations with low light levels	55	50.9	45.5	.701
Having a health summary document which can be shared with clinicians (e.g. hospital passport)	31	64.5	48.4	.302
A clinician who uses an approach which is informed by what you have said that you prefer (e.g. formal or informal)	58	29.5	31.0	.690
Identifying reasons that make it difficult to see a clinician or attend an appointment	43	48.8	46.5	1.000
Short waiting times to be seen when you attend appointments	91	29.7	36.3	.451
Provide support in relation to attending appointments (e.g. managing fears or uncertainties which might make attending difficult)	55	45.5	36.4	.442
Appropriate distractions provided whilst waiting to be seen at appointment (e.g. tablet with headphones)	51	74.5	72.5	1.000

For peer review only

STROBE Statement—Checklist of items that should be included in reports of ***cross-sectional studies***

	Item No	Recommendation	Page(s)	Line Number(s)
**Title and abstract**	1	(*a*) Indicate the study's design with a commonly used term in the title or the abstract	2	24
		(*b*) Provide in the abstract an informative and balanced summary of what was done and what was found	2-3	20-45
**Introduction**				
Background/rationale	2	Explain the scientific background and rationale for the investigation being reported	4-6	62-111
Objectives	3	State specific objectives, including any prespecified hypotheses	6	113-120
**Methods**				
Study design	4	Present key elements of study design early in the paper	6	124-126
Setting	5	Describe the setting, locations, and relevant dates, including periods of recruitment, exposure, follow-up, and data collection	6-8	128-175
Participants	6	(*a*) Give the eligibility criteria, and the sources and methods of selection of participants	6-7	129-162
Variables	7	Clearly define all outcomes, exposures, predictors, potential confounders, and effect modifiers. Give diagnostic criteria, if applicable	9-11	188-257
Data sources/ measurement	8*	For each variable of interest, give sources of data and details of methods of assessment (measurement). Describe comparability of assessment methods if there is more than one group	9-11	188-257
Bias	9	Describe any efforts to address potential sources of bias	10, 24	253, 490, 496
Study size	10	Explain how the study size was arrived at	8, 11	165-178, 238-239
Quantitative variables	11	Explain how quantitative variables were handled in the analyses. If applicable, describe which groupings were chosen and why	10, 11	213, 234-235, 243, 252-255
Statistical methods	12	(*a*) Describe all statistical methods, including those used to control for confounding	10-11	219-257
		(*b*) Describe any methods used to examine subgroups and interactions	10, 11	219-224, 249-257
		(*c*) Explain how missing data were addressed	11	235-238
		(*d*) If applicable, describe analytical methods taking account of sampling strategy	11	238-241
		(*e*) Describe any sensitivity analyses	N/A	N/A
**Results**				
Participants	13*	(a) Report numbers of individuals at each stage of study—eg numbers potentially eligible, examined for eligibility, confirmed eligible, included in the	7	146-150, 156-158, Table 2, Table 3

For peer review only

For peer review only - http://bmjopen.bmj.com/site/about/guidelines.xhtml

		study, completing follow-up, and analysed		
		(b) Give reasons for non-participation at each stage	7	146-150, 156-158
		(c) Consider use of a flow diagram	N/A	N/A
Descriptive data	14*	(a) Give characteristics of study participants (eg demographic, clinical, social) and information on exposures and potential confounders	13	Table 1
		(b) Indicate number of participants with missing data for each variable of interest	17, 19	Table 2, Table 3, Supplementary Tables 5&6
Outcome data	15*	Report numbers of outcome events or summary measures	N/A	N/A
Main results	16	(*a*) Give unadjusted estimates and, if applicable, confounder-adjusted estimates and their precision (eg, 95% confidence interval). Make clear which confounders were adjusted for and why they were included	21, Supplementary Info	403-455, Bonferroni Adjustments for McNemar (CIs N/A)
		(*b*) Report category boundaries when continuous variables were categorized	N/A	N/A
		(*c*) If relevant, consider translating estimates of relative risk into absolute risk for a meaningful time period	N/A	N/A
Other analyses	17	Report other analyses done—eg analyses of subgroups and interactions, and sensitivity analyses	12, 21	260-282, 400-408
**Discussion**				
Key results	18	Summarise key results with reference to study objectives	21-23	411-474
Limitations	19	Discuss limitations of the study, taking into account sources of potential bias or imprecision. Discuss both direction and magnitude of any potential bias	24	486-498
Interpretation	20	Give a cautious overall interpretation of results considering objectives, limitations, multiplicity of analyses, results from similar studies, and other relevant evidence	21-23	411-474
Generalisability	21	Discuss the generalisability (external validity) of the study results	21-22	419-427
**Other information**				
Funding	22	Give the source of funding and the role of the funders for the present study and, if applicable, for the original study on which the present article is based	27	565-571

*Give information separately for exposed and unexposed groups.

Note: An Explanation and Elaboration article discusses each checklist item and gives methodological background and published examples of transparent reporting. The STROBE checklist is best used in conjunction with this article (freely

For peer review only

For peer review only - http://bmjopen.bmj.com/site/about/guidelines.xhtml

available on the Web sites of PLoS Medicine at http://www.plosmedicine.org/, Annals of Internal Medicine at http://www.annals.org/, and Epidemiology at http://www.epidem.com/). Information on the STROBE Initiative is available at www.strobe-statement.org.

For peer review only

For peer review only - http://bmjopen.bmj.com/site/about/guidelines.xhtml

BMJ Open

The importance and availability of adjustments to improve access for autistic adults who need mental and physical healthcare: findings from a UK survey

Journal:	*BMJ Open*
Manuscript ID	bmjopen-2020-043336.R2
Article Type:	Original research
Date Submitted by the Author:	22-Dec-2020
Complete List of Authors:	Brice, Samuel; Newcastle University Faculty of Medical Sciences, Population Health Sciences Institute<br>Rodgers, Jacqui; Newcastle University Faculty of Medical Sciences, Population Health Sciences Institute<br>Ingham, Barry; Newcastle University Faculty of Medical Sciences, Population Health Sciences Institute; Cumbria Northumberland Tyne and Wear NHS Foundation Trust<br>Mason, David; Newcastle University Faculty of Medical Sciences, Population Health Sciences Institute<br>Wilson, Colin ; Newcastle University Faculty of Medical Sciences, Population Health Sciences Institute<br>Freeston, Mark; Newcastle University Faculty of Medical Sciences, School of Psychology<br>Le Couteur , Ann; Newcastle University Faculty of Medical Sciences, Population Health Sciences Institute<br>Parr, Jeremy; Newcastle University Faculty of Medical Sciences, Population Health Sciences Institute; Cumbria Northumberland Tyne and Wear NHS Foundation Trust
**Primary Subject Heading**:	Health services research
Secondary Subject Heading:	Mental health
Keywords:	MENTAL HEALTH, Quality in health care < HEALTH SERVICES ADMINISTRATION & MANAGEMENT, Organisational development < HEALTH SERVICES ADMINISTRATION & MANAGEMENT

SCHOLARONE™
Manuscripts

I, the Submitting Author has the right to grant and does grant on behalf of all authors of the Work (as defined in the below author licence), an exclusive licence and/or a non-exclusive licence for contributions from authors who are: i) UK Crown employees; ii) where BMJ has agreed a CC-BY licence shall apply, and/or iii) in accordance with the terms applicable for US Federal Government officers or employees acting as part of their official duties; on a worldwide, perpetual, irrevocable, royalty-free basis to BMJ Publishing Group Ltd ("BMJ") its licensees and where the relevant Journal is co-owned by BMJ to the co-owners of the Journal, to publish the Work in this journal and any other BMJ products and to exploit all rights, as set out in our licence.

The Submitting Author accepts and understands that any supply made under these terms is made by BMJ to the Submitting Author unless you are acting as an employee on behalf of your employer or a postgraduate student of an affiliated institution which is paying any applicable article publishing charge ("APC") for Open Access articles. Where the Submitting Author wishes to make the Work available on an Open Access basis (and intends to pay the relevant APC), the terms of reuse of such Open Access shall be governed by a Creative Commons licence – details of these licences and which Creative Commons licence will apply to this Work are set out in our licence referred to above.

Other than as permitted in any relevant BMJ Author's Self Archiving Policies, I confirm this Work has not been accepted for publication elsewhere, is not being considered for publication elsewhere and does not duplicate material already published. I confirm all authors consent to publication of this Work and authorise the granting of this licence.

For peer review only

The importance and availability of adjustments to improve access for autistic adults who need mental and physical healthcare: findings from a UK survey

Authors

Samuel Brice samuel.brice@ncl.ac.uk

Jacqui Rodgers jacqui.rodgers@ncl.ac.uk

Barry Ingham barry.ingham@cntw.nhs.uk

David Mason david.mason@ncl.ac.uk

Colin Wilson colin.wilson@ncl.ac.uk

Mark Freeston mark.freeston@ncl.ac.uk

Ann Le Couteur a.s.le-couteur@ncl.ac.uk

Jeremy R. Parr jeremy.parr@ncl.ac.uk (corresponding author)

For peer review only - http://bmjopen.bmj.com/site/about/guidelines.xhtml

Abstract

Objectives: To investigate autistic people's views on the importance and availability of adjustments to mental and physical healthcare provision. To explore whether specific categories of adjustments can be identified and to identify any differences in their importance and availability between mental and physical healthcare.

Design: Data from two studies, both employing a cross-sectional survey design.

Setting: UK based autistic adults registered with the Adult Autism Spectrum Cohort-UK were contacted by post or online. In both studies, recruitment was staged over a 12-month period. Non-responders were sent a single reminder letter two weeks after initial contact.

Participants: 537 autistic adults completed a survey about mental health services (51% response rate), 407 completed the physical health survey (49% response rate). Within these samples, 221 participants completed both surveys.

Primary outcome measures: Each study developed a bespoke survey to explore participants' views on mental and physical health services respectively. Both included an identical list of adjustments that participants rated based on importance and availability.

Results: Three factors of important adjustments were identified: sensory environment, clinical and service context, and clinician knowledge and communication. Adjustments across healthcare settings were widely rated as being important yet rarely available. One significant difference between the importance of adjustments available through mental and physical health services was identified. Participants reported that having access to a clinician who is willing to adapt their approach to suit the person's preferences was significantly more important for participants attending mental health settings ($p = .001$).

Conclusions: Autistic people reported the limited availability of important adjustments in current healthcare provision. To address unmet need and tackle the health inequalities faced by autistic people attending physical and mental healthcare settings, healthcare providers should

For peer review only - http://bmjopen.bmj.com/site/about/guidelines.xhtml

offer adjustments relating to the three identified factors. Future research should focus on identifying and addressing service-provider barriers to implementation.

Strengths and limitations of this study

- This research matched published research priorities of the autism community and focused directly on the experiences of autistic people
- This study reports on key adjustments to both physical and mental health services
- Our data driven approach enabled us to determine overarching categories of adjustments that could be used to inform both policy and practice in relation to service development
- A notable proportion of participants reported that they did not have sufficient knowledge to comment on the availability of adjustments
- This study did not seek the opinions of health service providers, which should be a focus for future implementation research

For peer review only

Introduction

Autism Spectrum Disorder[1] is characterised by lifelong persistent difficulties with social communication and social interaction and the presence of patterns of restrictive and repetitive behaviours or interests that may include hyper or hyporeactivity to sensory aspects of the environment (1). At least 1.1% of the United Kingdom (UK) population is autistic (2).

Autistic people are more likely to experience mental and physical health conditions than the general (neurotypical) population (3); this persists across the lifespan (4). Poor health has been shown to predict poorer quality of life (5, 6). Autistic adults are more likely than neurotypical adults to experience physical health conditions, such as epilepsy, cardiovascular disease and diabetes (7). Anxiety and depression are particularly common, with an estimated lifetime prevalence of around 40% (8, 9). Autistic people are also more likely to experience multiple mental and physical health conditions concurrently (10). Epidemiological studies suggest autistic people are also significantly more likely to face premature mortality: the average life expectancy for an autistic adult without intellectual disability is 12 years lower than for neurotypical people (11).

Barriers to healthcare access

Autistic adults face a number of significant barriers to effective health care access and treatment (12, 13). To date, the most frequently reported barriers have been considered within three factors: individual-level (e.g. barriers to effective communication with clinicians, sensory sensitivities), provider-level (e.g. lack of 'autism awareness' amongst clinicians) and system-

[1] We will use the term 'autism' or 'autistic person' throughout this paper, reflecting a growing consensus that this terminology is preferred by the autism community. This terminology includes previously recognised subcategories of autism, including but not limited to Asperger's Disorder, Autistic Disorder and Pervasive Developmental Disorder.

For peer review only - http://bmjopen.bmj.com/site/about/guidelines.xhtml

level (e.g. convoluted referral pathways and lack of availability of specialist services). Although such attempts to categorise adjustments in this way are useful to provide a simplified message to healthcare providers, this categorisation system has yet to receive empirical support. Recent research indicates that these barriers prevent autistic people from arranging and attending healthcare appointments (14). In addition, other individual factors such as difficulties coping with the potential uncertainty of healthcare situations (15), and pre-existing anxiety (8) may make the experience of attending healthcare appointments especially problematic for autistic people. Further, although the UK National Health Service (NHS) is publicly funded and free at the point of access, in other countries the affordability of healthcare has been shown to be a barrier that is disproportionately more likely to affect autistic people (16). There is also evidence of a lack of training, skills and/or confidence among healthcare professionals in working with and delivering interventions to autistic people (17), which may impact on their continued engagement with healthcare services (12).

UK autism research priorities focus on aspects of physical and mental healthcare, including 'How should service delivery for autistic people be improved and adapted in order to meet their needs?' (18). Many autistic people report dissatisfaction with the current accessibility of healthcare across both physical and mental health settings (19, 20). Several recent UK clinical guidelines have recommended that to reduce barriers to healthcare, providers should adjust their procedures and approach to best meet the individual needs of autistic people, with additional specific guidance for mental health services, to meet the needs of people with intellectual disabilities (21-23). A recent study concluded that "adjustments for communication needs are as necessary for autistic people as ramps are for wheelchair users" (14). Evidence suggests that adjustments to services such as reducing exposure to potentially aversive sensory stimuli (e.g. noisy waiting rooms) and the clinician using the autistic adult's preferred

For peer review only - http://bmjopen.bmj.com/site/about/guidelines.xhtml

communication methods, do facilitate effective healthcare access for autistic people (13). However, the importance and availability of specific adjustments across healthcare settings is unknown.

Aims

1. To investigate autistic people's views on the importance and availability of adjustments that are designed to promote the accessibility and acceptability of mental and physical healthcare provision.

2. To explore whether specific categories of adjustments can be identified using exploratory factor analysis and to identify any differences in their importance and availability between mental and physical healthcare.

Method

Design

This study reports on data from two UK studies – one focused on anxiety (as an exemplar of a mental health condition) and the other focused on physical health conditions. Both employed a cross-sectional survey design and were undertaken in 2018-2019.

Participants

Participants for both studies were recruited via the Adult Autism Spectrum Cohort – UK (ASC-UK; https://research.ncl.ac.uk/adultautismspectrum/), a longitudinal cohort study of autistic adults. ASC-UK recruits from a wide variety of UK sources including NHS diagnostic/health services, charitable/community organisations and also receives self-referrals. In line with previous research (e.g. 6), these studies included some participants who were awaiting autism

For peer review only - http://bmjopen.bmj.com/site/about/guidelines.xhtml

assessment or suspected that they were autistic (18% and 16% of mental health and physical health samples respectively).

Participants completed paper or web-based materials, depending on their preference. Following consent, participants completed a registration questionnaire that included questions on demographics, mental and physical health, education, employment, and aspects of daily living. Participants also completed the Social Responsiveness Scale -2nd Edition (SRS-2, (24)).

The Personalised Anxiety Treatment – Autism (PAT-A) study investigated autistic people's experiences of anxiety and access to mental healthcare, with a particular focus on accessing psychological therapies, the primary treatment choice for anxiety conditions in UK clinical guidance (22). Inclusion criteria were autistic people living in the UK aged 18 years and older. 1113 autistic people aged 18 and over who had previously reported a diagnosed or suspected anxiety condition were invited to participate. 568 (51%) returned the consent form and completed the survey. Thirty-one participants consented and did not respond to any of the questions about adjustments and were not included in analyses, resulting in a final sample of 537.

The Improving the Health of Autistic People (IHOAP) study investigated the physical health of autistic people, and experiences of accessing healthcare for physical health conditions, including impact on daily life, screening services offered and taken up, and factors that made accessing health care difficult. Inclusion criteria were autistic people living in the UK aged 18 years and older. 945 autistic adults were invited to participate; 461 (49%) people consented and completed the survey. 54 participants did not respond to any survey questions about adjustments and were therefore not included in analyses, resulting in a final sample of 407.

For peer review only - http://bmjopen.bmj.com/site/about/guidelines.xhtml

There were 723 unique participants in the two studies, including 221 participants who completed both studies. Demographic information (from ASC-UK) for participants from the PAT-A and IHOAP studies is provided in Table 1.

Procedure

Eligible participants were sent an information sheet (including an 'easyread' version), consent form and survey, either by post or email depending on their contact preferences. Those preferring to be contacted online were sent a unique link based on a participant number and completed the consent form and survey using Qualtrics© (25). Participants who received materials by post completed a written consent form and survey and returned this to the research team in a prepaid envelope. Written informed consent was provided by all participants. Participants who had not responded after two weeks were sent a single reminder letter. Recruitment was staged over a 12-month period. Participation was not incentivised. All procedures were approved by the NHS Health Research Authority and a Research Ethics Committee (both Wales REC 5, Approval reference numbers: PAT-A: 18/WA/0014; IHOAP: 18/WA/0191).

Patient and public involvement (PPI)

This research is directly matched to published research priorities of the autism community (18). The ASC-UK cohort study involved autistic people at the design stage as well as in the design and delivery of ongoing associated projects. Prior to commencing both studies, PPI focus groups were convened with community stakeholders to discuss and agree to the methods and materials, including survey questions. An autistic person and parent of an autistic person are co-investigators on this research. Author CW is autistic and was involved with the analysis of

For peer review only

data and preparation of this manuscript. Our dissemination plan includes consideration of the most effective ways to share these findings with autistic people, including presentations and newsletter updates.

Measures

Both samples completed the same survey about adjustments to services. Both samples also provided data on prior service use and experiences of being offered adjustments. Other data were also collected as part of the PAT-A and IHOAP studies and will be reported separately (manuscripts in process). The survey questionnaires included a list of adjustments previously reported to improve access, inclusivity and appropriateness of health services, together with an option for open text responses to record any other adjustments. The list was based on published evidence (13), clinical experience and the Reasonable Adjustments Flag (NHS Digital, https://digital.nhs.uk/services/reasonable-adjustment-flag; (26)). The list of adjustments can be seen in Tables 2 and 3.

Participants were asked to rate the importance of each adjustment on a five-point Likert scale from 1) not at all important; 2) not very important; 3) neither important nor unimportant; 4) somewhat important and 5) very important. Participants also rated how available they thought each adjustment was using a five-point scale 1) never available, 2) rarely available, 3) available about half the time, 4) available most of the time, 5) available all of the time. For availability, a 6th option ‘I don’t know’ was provided to enable those who did not feel that they had enough experience of that particular adjustment to report this, enabling the research team to discriminate between lack of experience and a missing response. Any additional adjustments identified by participants in open text response boxes were recorded.

For peer review only - http://bmjopen.bmj.com/site/about/guidelines.xhtml

The Social Responsiveness Scale – 2nd Edition (SRS-2; (24) is a widely used standardised self-report questionnaire used to characterise autism 'severity'. The SRS-2 has demonstrated excellent internal consistency and the predictive validity of the adult form has been reported to have a specificity level of .60 and a sensitivity of .86 (27). The full-scale score was used to make comparisons between those who self-reported a diagnosis of autism and those who suspected they had autism or were awaiting assessment; and between responders and non-responders (further detail in Table 1 and Supplementary Table 1).

Analysis

Key demographic characteristics (age, gender and level of autism traits) were compared between groups using independent t-tests to compare means or chi-square tests to compare the distribution of categorical variables. Comparisons were made between participants who self-reported an autism diagnosis, and those who suspected that they were autistic or were awaiting an autism diagnosis; responders vs non-responders (in both the mental health and physical health studies) and between participants from the mental health and physical health samples. These comparisons were made to assess the representativeness and comparability of the samples and to aid with interpretation of the findings. Participants who completed both studies were not included in any statistical comparisons made between the mental health and physical health samples to avoid double counting and so breaking the assumption of independence.

Exploratory factor analysis (EFA) was conducted in order to assess whether the adjustments clustered together in a meaningful way. FACTOR software version 10.8.04 (28) was used to conduct EFA on data on the importance of each adjustment in mental health and physical health services separately. Polychoric correlations were used as this is deemed most appropriate for

For peer review only

For peer review only - http://bmjopen.bmj.com/site/about/guidelines.xhtml

use with ordinal and/or skewed data such as these (29). Unweighted least squares were used to extract the factors and the rotation method was Promin. The use of an oblique method of rotation such as this allows for the correlation between factors to be calculated, which is in line with best scientific practice (30). Bias correlated bootstrap resampling was undertaken using 5000 bootstrap samples. This decision was taken in order to reduce the influence of any outliers and maximise the generalisability of the sample. Model fit was assessed using the following indices: Root Mean Square Error of Approximation (RMSEA), Non-Normed Fit Index (NNFI), Comparative Fit Index (CFI), Goodness of Fit Index (GFI) and Root Mean Square of Residuals (RMSR).

Participants with at least one missing response on any of the importance of adjustment ratings were deleted listwise prior to conducting EFA as any efforts to impute missing items may have biased the results at this stage. This resulted in the removal of 51 participants from the mental health dataset (final N = 486 and 78 participants from the physical health dataset (N = 329). Sample size suitability for EFA has been a matter of debate, however best practice guidelines suggest that a participant-to-item ratio of between 5:1 and 10:1 is considered to be suitable (31). Therefore, the 27:1 and 18.3/1 participant-to-item ratios in the mental health and physical health datasets respectively were deemed more than adequate for EFA. Furthermore, the Kaiser-Meyer-Olkin (KMO) test revealed that the sampling adequacy was ‘very good’ in both datasets (mental health: KMO = .92; physical health: KMO = .91).

Decisions regarding the number of factors to retain was based on best practice guidelines (31), which included consulting existing theoretical knowledge, examining the scree plots, retaining factors with an eigenvalue greater than one and conducting parallel analysis. These indicators supported the retention of between two and four factors in each of the datasets. An a-priori

For peer review only - http://bmjopen.bmj.com/site/about/guidelines.xhtml

loading criterion of ≥ .4 was applied to the rotated loading matrices to ensure that emergent factors were meaningful, whilst not being so stringent as to limit the reproducibility of the findings (32) .

Quantitative data were analysed descriptively. Analysis of open text comments and descriptions of experiences about adjustments used framework analysis (33). Two researchers (JR and SB) coded open text responses against the adjustment categories indicated in EFA and any discrepancies were discussed and agreed upon. Additional adjustments that did not fit within these categories were recorded under the category 'other'.

Service-level differences (i.e. comparisons between mental healthcare and physical healthcare) in adjustments that were reported by autistic people as most important and least available were explored using McNemar's test to compare nominal data in the paired sample of 221 participants who completed the PAT-A and IHOAP surveys. Variables were first dichotomized to separate the proportion who selected that an adjustment was 'very important' compared with all other levels of importance and 'never available' compared with all other levels of availability. McNemar's comparisons were computed for each of the 18 core adjustments based on the dichotomised importance and availability ratings. Simes' modified Bonferroni correction (34) was used to minimise risk of type I error.

Results

Details of statistical comparisons between the study participants and those who did not respond, and within and between subsamples (i.e. mental health and physical health samples) can be seen in Supplementary Table 1. There was no significant difference in autism characteristics (SRS-2 total score) between those who reported an autism spectrum diagnosis and those who

For peer review only - http://bmjopen.bmj.com/site/about/guidelines.xhtml

suspected they were autistic or were awaiting diagnosis in all comparison groups. The only significant differences were: the physical health sample (mean age= 44.4, SD= 13.4) were significantly older than the mental health sample (mean age= 41.3, SD= 13.8); those who took part in the physical health study were significantly older (mean age= 44.4, SD= 13.4) than non-responders (mean age= 41.0, SD= 13.3).

Table 1: Comparison of the demographic characteristics of participants included in the mental health and physical health samples

**Variable**	**Mental Health Sample (PAT-A study)**	**Physical Health Sample (IHOAP study)**
**Total N**	537	407
**Gender N (%)**		
Male	234 (43.6)	167 (41.0)
Female	281 (52.3)	227 (55.8)
Other/rather not say	22 (4.1)	13 (3.2)
**Age: Mean (SD) [Range]**	41.3 (13.8) [18.0-77.0]	44.4 (13.4) [18.0-79.0]
**Ethnicity N (%)**		
White British	501 (93.3)	374 (91.9)
Asian	3 (0.6)	1 (0.2)
Black	2 (0.4)	3 (0.7)
Mixed	10 (1.9)	8 (2.0)
Other/rather not say	8 (1.5)	7 (1.7)
Not reported	13 (2.4)	14 (3.4)
**Highest education N (%)**		
Postgraduate degree	94 (17.5)	72 (17.7)
Bachelor's degree	135 (25.1)	97 (23.8)
Diploma of higher education	34 (6.3)	28 (6.9)
Certificate of higher education	18 (3.4)	17 (4.2)
A-level	88 (16.4)	76 (18.7)

For peer review only

For peer review only - http://bmjopen.bmj.com/site/about/guidelines.xhtml

GCSE	108 (20.1)	71 (17.4)
Basic skills	20 (3.7)	10 (2.5)
No formal qualifications	32 (6.0)	30 (7.4)
Other	8 (1.5)	6 (1.5)
**Employment status N (%)**		
Employed without support[a]	213 (39.7)	154 (37.8)
Employed with support	9 (1.7)	5 (1.2)
Volunteer	49 (9.1)	38 (9.3)
Unemployed	170 (31.7)	137 (33.7)
Retired	30 (5.6)	25 (6.1)
Other	58 (10.8)	38 (9.3)
No response	8 (1.5)	10 (2.5)
**ASD diagnosis N (%)**		
Formal diagnosis	443 (82.5)	341 (83.8)
Suspected/unsure/awaiting assessment	94 (17.5)	66 (16.2)
**Age at diagnosis: Mean (SD) [Range]**	35.8 (15.8) [3.0-73.0]	38.7 (14.8) [2.0-68.0]
**SRS score[b] by severity category N (%)**		
Normal	20 (4.2)	9 (2.5)
Mild	45 (9.4)	35 (9.7)
Moderate	172 (35.8)	115 (31.9)
Severe	244 (50.7)	201 (55.8)
Mean (SD)	110.5 (25.6)	115.1 (25.8)
**Support received N (%)**		
Home	152 (15.5)	115 (15.2)
Employment	50 (5.1)	36 (4.8)
Health	110 (11.2)	95 (12.6)
Finance	129 (13.2)	99 (13.1)
Social	75 (7.7)	57 (7.5)
Lifelong learning	67 (6.9)	47 (6.2)
Community	78 (8.0)	68 (9.0)

For peer review only

For peer review only - http://bmjopen.bmj.com/site/about/guidelines.xhtml

Organisation	73 (7.5)	55 (7.3)
Do not receive support	244 (24.9)	183 (24.2)
**Mental health condition N (%)**		
Diagnosis	-	351 (86.2)
No diagnosis or suspected	-	56 (13.8)
**Physical health condition N (%)**		
Diagnosis	397 (73.9)	-
No diagnosis or suspected	140 (26.1)	-
**Prior service use (anxiety) N (%)**		
Prescribed medication	394 (73.4)	-
Referred for psychological therapy	400 (76.5)	-
Completed treatment	320 (80.0)	-
Did not complete /attend	80 (20.0)	-
Offered adjustments (mental health services)	90 (24.3)	-
**Prior service use (physical health) N (%)**		
Seen GP in the preceding 12 months	-	375 (92.0)
Seen specialist in the preceding 24 months	-	240 (59.0)
Require regular appointments due to a medical condition	-	240 (59.0)
Missed at least one appointment with a specialist	-	58 (24.2)
Offered adjustments (physical health services)	-	75 (19.8)

a- includes self-employed; b- not available for all participants; c- may add to more than total sample size as multiple responses were allowed

Abbreviations: SD = Standard Deviation, ASD = Autism Spectrum Disorder, SRS = Social Responsiveness Scale – 2nd Edition

For peer review only

For peer review only - http://bmjopen.bmj.com/site/about/guidelines.xhtml

Adjustments and service use

69% (n= 193) of autistic people who were not offered adjustments by mental health services and 56% (n= 171) of those not offered adjustments by physical health services suggested that adjustments would have been required or helpful. Of those who attended at least one session of psychological therapy but did not complete their treatment 64% (n= 40) cited a lack of adjustments to meet their needs as a contributing factor. Poor accessibility or lack of adjustments were a factor for 45% (n= 26) of those who reported missing at least one physical health appointment.

Categorising key adjustments to services

Exploratory factor analysis (EFA) supported the retention of between two and four factors. Using an a-priori loading criterion of .4 a clear three-factor solution was retained in the mental health data (factor loadings of >.4 for 17/18 items; intercorrelations: .69 to .79). The factors related to adjustments for 1) 'sensory environment', 2) 'clinical and service context' and 3) 'clinician knowledge and communication'. The adjustment '*short waiting times to be seen when you attend appointments*' did not load clearly onto any factor. This solution demonstrated very good/excellent model fit across all tested indices. A four-factor solution was most interpretable for the physical health data (factor loadings of > .4 for 15/18 items and good model fit). Closer inspection indicated strong similarities across both solutions. Thirteen out of 18 adjustments loaded onto the same, or similar factors. Data for the item relating to shorter waiting times are presented alone (see Supplementary tables 2, 3 and 4 for factor loadings and model fit indices). The importance (Table 2) and availability (Table 3) of adjustments are presented in the categories identified by factor analysis. Full response frequencies for the importance of adjustments can be seen in Supplementary Table 5 and availability of adjustments in Supplementary Table 6.

For peer review only - http://bmjopen.bmj.com/site/about/guidelines.xhtml

Table 2. The importance of key adjustments to mental and physical health services to meet the needs of autistic adults

Key adjustment	Mental Health Services		Physical Health Services	
	Somewhat Important % (N)	Very important % (N)	Somewhat Important % (N)	Very important % (N)
**F1: Sensory Environment**				
Change the sensory environment in the building that the appointment will take place in	33.6 (178)	39.4 (209)	31.5 (123)	42.5 (166)
Locations (e.g. waiting rooms) with small numbers of people	30.6 (162)	50.7 (268)	29.4 (116)	52.8 (208)
Locations with low noise levels	28.2 (149)	60.9 (322)	26.4 (104)	61.9 (244)
Locations with low light levels	29.1 (153)	35.2 (185)	31.3 (121)	35.7 (138)
**F2: Clinical and Service Context**				
Changing the length of appointments to suit you	33.8 (181)	36.2 (194)	33.9 (134)	44.8 (177)
Offering appointments online or via apps	22.9 (120)	22.9 (120)	18.8 (73)	31.2 (121)
Changing how often you are asked to attend appointments	30.7 (163)	24.5 (130)	28.6 (109)	18.1 (69)
Give information to the clinician pre-appointment so that they can prepare	27.7 (147)	60.6 (322)	32.8 (130)	48.0 (190)
Provide support in relation to attending appointments (e.g. managing fears or uncertainties which might make attending difficult)	27.2 (146)	53.5 (282)	28.0 (109)	50.9 (198)
Appropriate distractions provided whilst waiting to be seen at appointment (e.g. tablet with headphones)	23.1 (121)	25.2 (132)	24.3 (93)	27.7 (106)
**F3: Clinician Knowledge and Communication**				
Clinicians who understand autism	10.7 (57)	87.6 (467)	16.0 (64)	80.5 (322)
Opportunity after the appointment to ask questions about conclusions	31.1 (166)	60.2 (321)	25.4 (100)	64.4 (253)
Appointments at an easily identified and accessible location	19.6 (104)	70.4 (374)	17.1 (70)	71.7 (281)
Appointments with an easily identified and familiar clinician	21.1 (112)	68.9 (365)	19.8 (78)	71.0 (279)
Having a health summary document which can be shared with clinicians (e.g. hospital passport)	28.9 (152)	51.0 (268)	26.0 (100)	48.8 (188)
A clinician who uses an approach which is informed by what you have said that you prefer (e.g. formal or informal)	31.6 (167)	60.2 (318)	30.6 (118)	50.0 (193)
Identifying reasons that make it difficult to see a clinician or attend an appointment	33.1 (174)	49.5 (260)	29.8 (114)	52.9 (202)
**Standalone item**				
Short waiting times to be seen when you attend appointments	32.5 (168)	55.1 (293)	28.2 (111)	57.8 (227)

For peer review only - http://bmjopen.bmj.com/site/about/guidelines.xhtml

Most respondents reported that all adjustments were 'somewhat important' or 'very important' (hereafter referred to as important) in both mental and physical health services. In mental health services, those rating each adjustment as important (i.e. 'somewhat important' or 'very important') ranged from 46% (n=240) (access to online appointments) to 98% (n= 524) (clinician who understands autism). In physical health services, these ranged from 47% (n= 178) (changing frequency of appointments) to 97% (n= 386) (clinician who understands autism). High proportions of participants also rated each adjustment as 'very important' ranging from 23% (n= 120) (access to online appointments) to 88% (n= 468) (clinician who understands autism) in mental health services and 18% (n= 69) (changing frequency of appointments) to 81% (n= 322) (clinician who understands autism) in physical health services. Autistic adults rated *'having access to a clinician who understands autism'* as the most important adjustment in both mental health (98.3%, n= 524) and physical health (96.5%, n= 386) settings.

For peer review only - http://bmjopen.bmj.com/site/about/guidelines.xhtml

Table 3: The lack of availability of key adjustments to mental and physical healthcare services to meet the needs of autistic adults

**Key adjustment**	**Mental Health Services**		**Physical Health Services**	
	Rarely available % (N)	Never available % (N)	Rarely available % (N)	Never available % (N)
**Sensory Environment**				
Change the sensory environment in the building that the appointment will take place in	26.6 (63)	45.6 (108)	23.1 (55)	60.5 (144)
Locations (e.g. waiting rooms) with small numbers of people	22.3 (63)	29.3 (83)	27.0 (72)	49.1 (131)
Locations with low noise levels	26.0 (72)	26.0 (72)	26.8 (73)	42.6 (116)
Locations with low light levels	24.3 (60)	40.9 (101)	30.9 (76)	52.4 (129)
**Clinical and Service Context**				
Changing the length of appointments to suit you	23.1 (52)	45.3 (102)	29.3 (68)	41.4 (96)
Offering appointments online or via apps	16.4 (32)	48.2 (94)	17.6 (41)	32.6 (76)
Changing how often you are asked to attend appointments	25.4 (46)	27.6 (50)	23.1 (34)	44.2 (65)
Give information to the clinician pre-appointment so that they can prepare	22.8 (50)	33.3 (73)	25.1(56)	41.3 (92)
Provide support in relation to attending appointments (e.g. managing fears or uncertainties which might make attending difficult)	21.7 (51)	40.4 (95)	30.7 (67)	45.9 (100)
Appropriate distractions provided whilst waiting to be seen at appointment (e.g. tablet with headphones)	15.7 (36)	63.3 (145)	18.3 (38)	66.3 (138)
**Clinician Knowledge and Communication**				
Clinicians who understand autism	40.5 (105)	31.3 (81)	36.1 (84)	36.9 (86)
Opportunity after the appointment to ask questions about conclusions	23.9 (63)	18.9 (50)	20.2 (52)	26.8 (69)
Appointments at an easily identified and accessible location	15.3 (47)	10.1 (31)	12.0 (55)	13.0 (38)
Appointments with an easily identified and familiar clinician	18.2 (52)	13.0 (37)	25.3 (73)	16.1 (47)
Having a health summary document which can be shared with clinicians (e.g. hospital passport)	17.7 (34)	52.1 (100)	17.9 (33)	63.6 (117)
A clinician who uses an approach which is informed by what you have said that you prefer (e.g. formal or informal)	27.9 (70)	23.5 (59)	20.8 (45)	42.1 (91)
Identifying reasons that make it difficult to see a clinician or attend an appointment	25.9 (55)	34.0 (72)	26.3 (52)	46.5 (92)
**Standalone item**				
Short waiting times to be seen when you attend appointments	25.1 (72)	27.2 (78)	33.9 (97)	35.7 (102)

For peer review only

For peer review only - http://bmjopen.bmj.com/site/about/guidelines.xhtml

For over half of respondents all but three adjustments were endorsed as 'unavailable' (i.e. rarely available' or 'never available') across both health service contexts. Adjustments were frequently endorsed as being 'never available' across both settings, ranging from 10% (n= 31) to 63% (n= 145) in mental health and 13% (n= 38) to 67% (n= 138) in physical health. In both cases, the adjustment most frequently rated as 'never available' was 'being provided with appropriate distractions whilst waiting for appointments' and the item least frequently rated as 'never available' was 'appointments at an easily identified and familiar location'. A significant proportion of respondents to both surveys did not feel that they were able to comment on the availability of adjustments (range = mental health: 42.5% – 62.4%; physical health: 19.4% - 47.2%).

Other adjustments endorsed by autistic people

16% (n= 85) of participants in the mental health survey and 14% (n= 56) in the physical health survey gave information in open text about an 'other' adjustment; some of these were not coded (e.g. general comments). The majority (90% mental health, 91% physical health) of open text comments were classified within the 3-factor solution (see Table 4). An additional adjustment *"Being able to bring a supporter such as a friend, family member (or support animal) to appointments"* was suggested by several participants in both health service contexts.

Table 4. Reporting the number of open text comments that could be categorised to the identified factors following framework analysis

	Mental Health Services	Physical Health Services
**Sensory Environment**	8	5
**Clinical and Service Context**	33	24
**Clinician Knowledge & Communication**	16	10
**Other**	6	4

For peer review only - http://bmjopen.bmj.com/site/about/guidelines.xhtml

Paired comparison of the importance and availability of adjustments between physical and mental health services

221 participants completed both surveys. After Bonferroni correction, only one adjustment was rated as being very important significantly more often in mental health (65%) compared with physical health settings (47%): *'a clinician who uses an approach which is informed by what you have said that you prefer'* (p = .001). No significant differences in the least available adjustments based on service type were observed. Full reporting of these analyses can be found in Supplementary Tables 7 and 8.

Discussion

This large study successfully identified for the first time autistic adults' views about the importance and availability of key adjustments that UK and international guidelines and research consider could improve accessibility and acceptability of healthcare services. Autistic adults strongly endorsed the importance of these adjustments; the relative importance of each adjustment was broadly equivalent across physical and mental health service contexts. However, autistic people experienced limited availability to key adjustments across both healthcare contexts – showing clear unmet clinical need. Given that autistic people are at increased risk of combinations of mental and physical health conditions, healthcare providers need to take action now to implement provision of adjustments to improve service accessibility. Furthermore, this UK sample was large, recruited nationwide, and was demographically diverse; as the majority of respondents reported they experienced both mental health conditions and physical health disorders, it is likely that these findings can be generalised to other autistic adults in the UK. This suggestion is further supported by the finding that there were no significant differences between the key demographic variables of responders and non-responders to the survey, with the exception of age in the physical health study (see

For peer review only - http://bmjopen.bmj.com/site/about/guidelines.xhtml

Supplementary Table 1). The availability and lack of availability of adjustments will however vary, not only within the UK, but also in other countries and especially those without publicly funded healthcare.

Three distinct but inter-correlated factors of adjustments were identified: 1) the sensory environment, 2) the clinical and service context and 3) clinician knowledge and understanding. These factors are consistent with recent syntheses of the evidence regarding the barriers to healthcare access for autistic people (12, 13) and recent NHS reasonable adjustment guidance (26), which include adjustments under the categories of environment, service delivery, and communication. Our data-driven approach to categorisation is novel. The findings support and extend previous theoretical attempts at categorisation of adjustments to healthcare. The majority of 'other' adjustments proposed by participants could be classified within the three-factor model, providing further validation. Participants also identified across both service contexts the benefit of having the opportunity to attend appointments with support (a friend, family member). In common with all the adjustments included in our survey, this adjustment is not on the face of it difficult to implement, and it is likely most services would be supportive if asked. The NHS reasonable adjustments policy does already include having support at appointments amongst the potential adjustments offered (26) and recommends that such a request should be discussed and noted.

The adjustment rated as being most important in both settings was 'access to a clinician who understands autism'. Indeed, the four most important adjustments endorsed across both healthcare settings related to the 'clinician knowledge and communication' factor. This finding is in keeping with previous findings that healthcare professionals may lack skills and/or confidence in delivering interventions to autistic people (17). A recent review found

For peer review only - http://bmjopen.bmj.com/site/about/guidelines.xhtml

that some autistic people feel reluctant to report their autism diagnosis to healthcare professionals for fear that it may negatively affect their care (35). This suggests that care must be taken to establish an environment whereby the patient feels confident enough to discuss their autism and what adjustments would help them fully access healthcare services openly. When the priority adjustments were compared across the paired sample, only one adjustment '*A clinician who uses an approach which is informed by what you have said that you prefer (e.g. formal or informal)'* was significantly different between healthcare contexts. Perhaps there is a perception that mental healthcare clinicians need to take into account individual preference; however, coming to a shared understanding and approach to healthcare is critical in any clinical setting. The discrepancy between the rated importance of the identified adjustments and their reported availability in both physical and mental health services makes stark reading. Our findings highlight the wide 'gap' in the perceived availability of adjustments reported as important. Closer examination of our findings on the availability of adjustments allows us to highlight potential shortfalls in UK health services. The high percentages of 'rarely or never available' responses seen across many adjustments suggests a systematic shortfall across the NHS rather than variability between services. However, encouragingly two adjustments [*appointments at a familiar location* and with a *familiar clinician*] were rated as being available more than rarely by over half of each sample. Offering consistency in clinical care was appreciated in both contexts. In keeping with recently published findings identifying widespread barriers to healthcare access for autistic people (12, 13), respondents consistently rated their experiences of not being able to access adjustments in healthcare. If autistic adults are not experiencing these adjustments to promote accessibility, they are likely to continue to experience delays in mental and physical health diagnosis, poor treatment outcomes (20), significant inequalities and premature mortality (11).

For peer review only - http://bmjopen.bmj.com/site/about/guidelines.xhtml

Strengths and Limitations

Our study has many strengths. The research aimed to match published autism community research priorities and focus directly on the experiences of autistic people (18). The study also includes experiences about adjustments in both mental and physical healthcare settings. Our large samples were both approximately equal in terms of male and female participants, which is a strength considering the low numbers of female participants often observed in autism research. Our data driven approach enabled us to determine key overarching factors or categories of adjustments that could be used to inform both policy and practice in relation to service development.

One limitation of our study is that some participants reported that they did not have sufficient knowledge to comment on the availability of adjustments and were not able to provide a rating. However, the use of paired comparisons gives confidence that any identified differences are a true reflection of differences between mental and physical healthcare by virtue of eliminating the potential for sources of between-person variance. Further, that a notable proportion of the sample reported they were not informed of the availability of adjustments they might want, is an important finding in itself. Our data are based on the reported experiences of the respondents. We did not aim to collect information directly from the services, and this should be done in future studies. However, the samples are large and recruited from diverse sources across the UK including the NHS, private and charity providers, so it is unlikely systematic bias was introduced. The participants in the mental health sample were all individuals who had self-reported either a diagnosed or suspected anxiety condition (relating to the aims of the original study). This may have the potential to limit the generalisability of the findings for other

For peer review only - http://bmjopen.bmj.com/site/about/guidelines.xhtml

mental health settings – although many mental health services have a similar service design, and clinicians tend to deliver care for a variety of mental health conditions.

Implications and future research

The findings of this study highlight the lack of key adjustments available to autistic adults currently accessing physical and mental healthcare. These adjustments do not require expensive technology or equipment but rather ongoing staff training, attention to aspects of the clinical settings and flexibility in ways of delivering clinical care. We consider that 'how' and 'what' adjustments can be made should be more explicit in pre-appointment information, and during appointments. Focussing on the sensory environment, clinical and service context, clinician knowledge and communication and involvement of supporters, may help service users, providers and commissioners of services to identify and address any barriers to implementation. Proactively considering these adjustments with autistic people is in our opinion a prudent and logical first step towards minimising the barriers to healthcare. This in turn is a crucial component of an individualised /personalised approach to assessment and management of identified healthcare needs in order to maximise treatment effectiveness and reduce health inequalities. Our current research is developing and evaluating personalised mental and physical healthcare provision for autistic people across the lifespan. Examples include the co-design (with autistic people, relatives and clinicians) and evaluation of a personalised, flexible psychological intervention for anxiety in autistic adults (PAT-A, 36). We have also co-designed an intervention to investigate how autistic people age 50 and over can be encouraged to access the healthcare they need (Trial registration: ISRCTN75726745). To address the primary care needs of autistic adults, we have co-designed and are evaluating the use of primary care healthchecks for autistic people (https://research.ncl.ac.uk/autismhealthchecks/). All these approaches systematically gather

For peer review only - http://bmjopen.bmj.com/site/about/guidelines.xhtml

information about the adjustments required to maximise the chances of success (details of the tools used to do this available on request). Co-designed approaches to healthcare that meets the needs of autistic people and clinicians should increase the opportunities for autistic people to access the personalised and precision health care that is increasingly available to all people in mental and physical health services. In turn, that will hopefully reduce the increased and premature morbidity and average earlier mortality for autistic people.

Author Details

Samuel Brice[1], Jacqui Rodgers[1], Barry Ingham[1,2], David Mason[1], Colin Wilson[1], Mark Freeston[3], Ann Le Couteur[1] & Jeremy R. Parr[1,2]

1. Population Health Sciences Institute, Newcastle University, UK
2. Cumbria, Northumberland, Tyne and Wear NHS Foundation Trust, UK
3. School of Psychology, Newcastle University, UK

Corresponding Author

Professor Jeremy R Parr

Email: Jeremy.Parr@ncl.ac.uk

Address: Level 3, Sir James Spence Institute, Royal Victoria Infirmary, Queen Victoria Road, Newcastle upon Tyne, NE1 4LP

Author contributions

JP, BI and JR conceptualised the adjustments study. JP and JR received funding for PAT-A. BI and JP received funding for IHOAP. JP and BI liaised with NHSE about adjustments. JR and MF supervised statistical analyses. SB and DM are researchers for PAT-A and IHOAP respectively and led data collection and undertook analysis. CW, MF and ALC provided

For peer review only

comment on PAT-A and adjustment study design and analyses. All authors contributed to adjustments study design, and writing of the manuscript, and reviewed the final version.

Declarations of Interest

MF reports personal fees from Honoraria from training organisations in the area of psychological therapies, personal fees from Royalties from books in the area of psychological therapies, outside of the submitted work. All other authors declare no conflicts of interest.

Acknowledgements

We are grateful to: all the participants who gave their time to complete the PAT-A and IHOAP surveys; the IHOAP investigators; the ASC-UK team including Dr Alex Petrou (who supported data collection), and the ASC-UK investigators, including Professor Helen McConachie. The Sponsor for the studies was Cumbria, Northumberland, Tyne and Wear NHS Foundation Trust.

Funding

Data from autistic adults were collected as part of two studies:

1. Personalised Anxiety Treatment for Autistic Adults study funded by the UK autism research charity Autistica. Funding was awarded to JP and JR as part of the autism spectrum adulthood and ageing research programme at Newcastle University (Grant ID: 7425).

2. Improving the Health of Older Autistic People study, funded by the Inge Wakehurst Trust, awarded to BI and JP (no grant number given)

Data Availability

For peer review only - http://bmjopen.bmj.com/site/about/guidelines.xhtml

Some data that support the findings of this study are available on reasonable request from the corresponding author [JP]. The data are not publicly available due to containing information that could compromise the privacy of research participants.

License Statement

I, the Submitting Author has the right to grant and does grant on behalf of all authors of the Work (as defined in the below author licence), an exclusive licence and/or a non-exclusive licence for contributions from authors who are: i) UK Crown employees; ii) where BMJ has agreed a CC-BY licence shall apply, and/or iii) in accordance with the terms applicable for US Federal Government officers or employees acting as part of their official duties; on a worldwide, perpetual, irrevocable, royalty-free basis to BMJ Publishing Group Ltd ("BMJ") its licensees and where the relevant Journal is co-owned by BMJ to the co-owners of the Journal, to publish the Work in BMJ Open and any other BMJ products and to exploit all rights, as set out in our licence.

The Submitting Author accepts and understands that any supply made under these terms is made by BMJ to the Submitting Author unless you are acting as an employee on behalf of your employer or a postgraduate student of an affiliated institution which is paying any applicable article publishing charge ("APC") for Open Access articles. Where the Submitting Author wishes to make the Work available on an Open Access basis (and intends to pay the relevant APC), the terms of reuse of such Open Access shall be governed by a Creative Commons licence – details of these licences and which Creative Commons licence will apply to this Work are set out in our licence referred to above.

References

1. American Psychiatric Association. Diagnostic and Statistical Manual of mental disorders (5th ed.). . Washington: APA; 2013.

2. Brugha TS, McManus S, Bankart J, Scott F, Purdon S, Smith J, et al. Epidemiology of autism spectrum disorders in adults in the community in England. Arch Gen Psychiatry. 2011;68(5):459-65.

3. Rydzewska E, Hughes-McCormack LA, Gillberg C, Henderson A, MacIntyre C, Rintoul J, et al. Prevalence of long-term health conditions in adults with autism: observational study of a whole country population. BMJ Open. 2018;8(8):e023945.

4. Hand BN, Angell AM, Harris L, Carpenter LA. Prevalence of physical and mental health conditions in Medicare-enrolled, autistic older adults. Autism. 2019;24(3):755-64.

5. Lawson LP, Richdale AL, Haschek A, Flower RL, Vartuli J, Arnold SR, et al. Cross-sectional and longitudinal predictors of quality of life in autistic individuals from adolescence to adulthood: The role of mental health and sleep quality. Autism. 2020;24(4):954-67.

6. Mason D, McConachie H, Garland D, Petrou A, Rodgers J, Parr JR. Predictors of quality of life for autistic adults. Autism Res. 2018;11(8):1138-47.

7. Croen LA, Zerbo O, Qian Y, Massolo ML, Rich S, Sidney S, et al. The health status of adults on the autism spectrum. Autism. 2015;19(7):814-23.

8. Hollocks MJ, Lerh JW, Magiati I, Meiser-Stedman R, Brugha TS. Anxiety and depression in adults with autism spectrum disorder: a systematic review and meta-analysis. Psychol Med. 2019;49(4):559-72.

9. Hudson CC, Hall L, Harkness KL. Prevalence of Depressive Disorders in Individuals with Autism Spectrum Disorder: a Meta-Analysis. J Abnorm Child Psychol. 2019;47(1):165-75.

10. Trembath D, Germano C, Johanson G, Dissanayake C. The Experience of Anxiety in Young Adults With Autism Spectrum Disorders. Focus on Autism and Other Developmental Disabilities. 2012;27(4):213-24.

11. Hirvikoski T, Mittendorfer-Rutz E, Boman M, Larsson H, Lichtenstein P, Bölte S. Premature mortality in autism spectrum disorder. British Journal of Psychiatry. 2016;208(3):232-8.

12. Bradshaw P, Pellicano E, van Driel M, Urbanowicz A. How Can We Support the Healthcare Needs of Autistic Adults Without Intellectual Disability? Current Developmental Disorders Reports. 2019;6(2):45-56.

13. Mason D, Ingham B, Urbanowicz A, Michael C, Birtles H, Woodbury-Smith M, et al. A Systematic Review of What Barriers and Facilitators Prevent and Enable Physical Healthcare Services Access for Autistic Adults. Journal of Autism and Developmental Disorders. 2019;49(8):3387-400.

14. Doherty M, Sullivan JD, Neilson SD. Barriers to healthcare for autistic adults: Consequences & policy implications. A cross-sectional study. medRxiv. 2020:2020.04.01.20050336.

15. Maisel ME, Stephenson KG, South M, Rodgers J, Freeston MH, Gaigg SB. Modeling the cognitive mechanisms linking autism symptoms and anxiety in adults. J Abnorm Psychol. 2016;125(5):692-703.

16. Nicolaidis C, Raymaker DM, Ashkenazy E, McDonald KE, Dern S, Baggs AE, et al. "Respect the way I need to communicate with you": Healthcare experiences of adults on the autism spectrum. Autism : the international journal of research and practice. 2015;19(7):824-31.

17. McCormack G, Dillon AC, Healy O, Walsh C, Lydon S. Primary Care Physicians' Knowledge of Autism and Evidence-Based Interventions for Autism: A Systematic Review. Review Journal of Autism and Developmental Disorders. 2019.

For peer review only

For peer review only - http://bmjopen.bmj.com/site/about/guidelines.xhtml

18. James Lind Alliance. Autism Top Ten 2016 [Available from: http://www.jla.nihr.ac.uk/priority-setting-partnerships/autism/top-10-priorities/.

19. Crane L, Adams F, Harper G, Welch J, Pellicano E. 'Something needs to change': Mental health experiences of young autistic adults in England. Autism. 2018:1362361318757048.

20. Nicolaidis C, Raymaker D, McDonald K, Dern S, Boisclair WC, Ashkenazy E, et al. Comparison of healthcare experiences in autistic and non-autistic adults: a cross-sectional online survey facilitated by an academic-community partnership. J Gen Intern Med. 2013;28(6):761-9.

21. National Development Team for Inclusion. Green Light Toolkit: A Guide to Auditing and Improving your Mental Health Service so that it is Effective in Supporting People with Autism and People with Learning Disabilities. 2017 [Available from: https://www.ndti.org.uk/uploads/files/Green_Light_Toolkit_2017.pdf.

22. National Institute for Health and Care Excellence. Autism spectrum disorder in adults: diagnosis and management. Clinical guideline [CG142]. 2012.

23. NHS England. The NHS Long Term Plan 2019 [Available from: https://www.longtermplan.nhs.uk/wp-content/uploads/2019/08/nhs-long-term-plan-version-1.2.pdf.

24. Gruber JNCCP. Social Responsiveness Scale–Second Edition (SRS-2). Torrance, CA: Western Psychological Services.; 2012.

25. Qualtrics. Qualtrics. Provo, Utah, USA2005.

26. NHS Digital. Reasonable Adjustment Flag [Available from: https://digital.nhs.uk/services/reasonable-adjustment-flag.

27. Mandell DS, Lawer LJ, Branch K, Brodkin ES, Healey K, Witalec R, et al. Prevalence and correlates of autism in a state psychiatric hospital. Autism. 2012;16(6):557-67.

28. Lorenzo-Seva U, Ferrando PJ. FACTOR 9.2: A Comprehensive Program for Fitting Exploratory and Semiconfirmatory Factor Analysis and IRT Models. Applied Psychological Measurement. 2013;37(6):497-8.

29. Baglin J. Improving your exploratory factor analysis for ordinal data: a demonstration using FACTOR. Practical Assessment, Research and Evaluation. 2014;19.

30. Osborne JW. What is Rotating in Exploratory Factor Analysis. Practical Assessment, Research and Evaluation. 2015;20.

31. Osborne JW. Best Practices in Exploratory Factor Analysis: Jason W. Osbourne; 2014.

32. Hoyle R, Duvall J. Determining the number of factors in exploratory and confirmatory factor analysis. 2004.

33. Srivastava A, Thomson SB. Framework analysis: a qualitative methodology for applied policy research. Journal of Administration and Governance. 2009;72.

34. Simes RJ. An Improved Bonferroni Procedure for Multiple Tests of Significance. Biometrika. 1986;73(3):751-4.

35. Walsh C, Lydon S, O'Dowd E, O'Connor P. Barriers to Healthcare for Persons with Autism: A Systematic Review of the Literature and Development of A Taxonomy. Developmental Neurorehabilitation. 2020:1-18.

36. Parr JR, Brice S, Welsh P, Ingham B, Le Couteur A, Evans G, et al. Treating anxiety in autistic adults: study protocol for the Personalised Anxiety Treatment–Autism (PAT-A©) pilot randomised controlled feasibility trial. Trials. 2020;21(1):265.

For peer review only - http://bmjopen.bmj.com/site/about/guidelines.xhtml

Supplementary Information

Supplementary Table 1: Demographic information for the mental health and physical health samples and comparison with non-responders

	Mental Health (PAT-A)		Physical Health (IHOAP)	
**Variable**	**Responders**	**Non-responders**	**Responders**	**Non-responders**
Total N	537	545	407	484
Gender N (%)				
Male	234 (43.6)	273 (50.1)	167 (41.0)	238 (49.1)
Female	281 (52.3)	254 (46.6)	227 (55.8)	236 (48.8)
Other/rather not say	22 (4.1)	18 (3.3)	13 (3.2)	10 (2.1)
Age: Mean (SD) [Range]	41.3 (13.8) [17.0-77.0]	37.6 (12.9) [17.0-84.0]	44.4 (13.4) [18.0-79.0]	41.0 (13.3) [18.0-90.0]
Ethnicity				
White	501 (93.3)	496 (91.0)	374 (91.9)	437 (90.3)
Asian	3 (0.6)	8 (1.5)	1 (0.2)	6 (1.2)
Black	2 (0.4)	3 (0.6)	3 (0.7)	2 (0.4)
Mixed	10 (1.9)	10 (1.8)	8 (2.0)	11 (2.3)
Other/rather not say	8 (1.5)	4 (0.7)	7 (1.7)	6 (1.2)
Not reported	13 (2.4)	24 (4.4)	14 (3.4)	26 (5.4)
Highest education				
Postgraduate degree	94 (17.5)	67 (12.3)	72 (17.7)	69 (14.3)
Bachelor's degree	135 (25.1)	122 (22.4)	97 (23.8)	97 (20.0)
Diploma of higher education	34 (6.3)	43 (7.9)	28 (6.9)	52 (10.7)
Certificate of higher education	18 (3.4)	27 (5.0)	17 (4.2)	19 (3.9)
A-level	88 (16.4)	115 (21.1)	76 (18.7)	93 (19.2)
GCSE	108 (20.1)	86 (15.8)	71 (17.4)	85 (17.6)
Basic skills	20 (3.7)	27 (5.0)	10 (2.5)	24 (5.0)
No formal qualifications	32 (6.0)	34 (6.2)	30 (7.4)	36 (7.4)
Other	8 (1.5)	24 (4.4)	6 (1.5)	9 (1.9)
Employment status N (%)				
Employed without support[a]	213 (39.7)	204 (37.4)	154 (37.8)	163 (33.7)
Employed with support	9 (1.7)	8 (1.5)	5 (1.2)	10 (2.1)
Volunteer	49 (9.1)	55 (10.1)	38 (9.3)	10 (2.1)
Unemployed	170 (31.7)	188 (34.5)	137 (33.7)	202 (41.7)
Retired	30 (5.6)	20 (3.7)	25 (6.1)	24 (5.0)
Other	58 (10.8)	57 (10.5)	38 (9.3)	62 (12.8)
No response	8 (1.5)	13 (2.4)	10 (2.5)	13 (2.7)
ASD diagnosis N (%)				
Formal diagnosis	443 (82.5)[d]	467 (85.7)	341 (83.8)[e]	415 (85.7)
Suspected/unsure/awaiting diagnosis	94 (17.5)[d]	78 (14.3)	66 (16.2)[e]	69 (14.3)
Age at diagnosis: Mean (SD) [Range]	35.8 (15.8) [3.0-73.0]	30.1 (15.5) [2.0-69.0]	38.7 (14.8) [2.0-68.0]	34.4 (15.8) [2.0-86.0]
SRS severity N (%)				
Normal	20 (4.2)	27 (7.2)	9 (2.5)	19 (5.7)
Mild	45 (9.4)	35 (9.3)	35 (9.7)	31 (9.2)
Moderate	172 (35.8)	112 (29.8)	115 (31.9)	97 (28.9)
Severe	244 (50.7)	202 (53.7)	201 (55.8)	189 (56.3)
SRS Mean (SD)	110.5 (25.6)	112.9 (29.2)	115.1 (25.8)	115.6 (28.2)
Support received[c] N (%)				
Home	152 (15.5)	162 (13.6)	115 (15.2)	142 (17.4)
Employment	50 (5.1)	78 (6.5)	36 (4.8)	53 (6.5)
Health	110 (11.2)	153 (12.8)	95 (12.6)	130 (16.0)
Finance	129 (13.2)	154 (12.9)	99 (13.1)	137 (16.8)
Social	75 (7.7)	115 (9.6)	57 (7.5)	88 (10.8)
Lifelong learning	67 (6.9)	75 (6.3)	47 (6.2)	60 (7.4)

For peer review only

Community	78 (8.0)	110 (9.2)	68 (9.0)	79 (9.7)
Organisation	73 (7.5)	110 (9.2)	55 (7.3)	93 (11.4)
Do not receive support	244 (24.9)	236 (19.8)	183 (24.2)	32 (3.9)
Mental health condition N (%)				
Diagnosis	-	-	351 (86.2)	414 (85.5)
No diagnosis	-	-	56 (13.8)	70 (14.5)
Physical health condition N (%)				
Diagnosis	397 (73.9)	402 (73.8)	-	-
No diagnosis	140 (26.1)	143 (26.2)	-	-
Response type				
Electronic	415 (77.3%	446 (81.8)	305 (74.9)	396 (81.8)
Paper	122 (22.7%)	99 (18.2)	102 (25.1)	88 (18.2)

Statistical comparison of key demographic factors within and between samples and between responders and non-responders

Tests of difference between responders and non-responders on key demographic variables

Significant differences are denoted with *

Age:
Mental health sample: $t(859)= 1.655$, $p=0.949$
Physical health sample: $t(668)= 3.369$, $p=0.001$*

Gender:
Mental health sample: M,F,O,R genders Mental health sample vs Physical health sample $\chi^2(3)=2.049$, $p=0.562$; males and females only: $\chi^2(1)=0.349$, $p=0.555$
Physical health sample: M,F,O,R genders Mental health sample vs Physical health sample $\chi^2(3)=4.481$, $p=0.214$; males and females only: $\chi^2(1)=1.007$, $p=0.316$

Autism Characteristics (SRS-2 total score):
Mental health sample*:* $t(641.5)= -1.135$, $p=0.257$
Physical health sample: $t(496)= -0.198$, $p=0.843$

Tests of difference in autism characteristics between those reporting an autism spectrum diagnosis and those who suspect that they are autistic or are awaiting diagnosis within subsamples

Mental health sample:
112.5 (autism diagnosis), 112.4 (no autism diagnosis). $t(479)= -0.002$, $p=0.998$

Physical health sample:
115.7 (autism diagnosis), 112.3 (no autism diagnosis). $t(358)= -0.893$, $p=0.372$)

For peer review only

Tests of difference between the mental health sample and the physical health sample on key demographic variables (Main Table 1)

Age:
$t(500) = -4.526$, $p<0.001$*

Gender:
$\chi^2(3)=2.493$, $p=0.477$

Autism Characteristics
$t(443) = -1.814$, $p=0.70$

For peer review only

Supplementary Table 2: Factor loadings for the three-factor solution for importance of adjustments to mental health services and four-factor solution for the importance of adjustments to physical health services

Adjustment	Factor Factor Code	Mental Health			Physical Health			
		F1 SE	F2 SC	F3 CKC	F1 SE	F2 SC	F3 CKC	F4 F
9. Change the sensory environment in the building that the appointment will take place in		**0.799**	0.064	0.006	**0.925**	-0.178	0.120	0.015
10. Locations (e.g. waiting rooms) with small numbers of people		**0.787**	-0.001	0.081	**0.621**	0.258	-0.212	0.245
11. Locations with low noise levels		**1.065**	-0.186	-0.054	**0.905**	-0.094	-0.051	0.064
12. Locations with low light levels		**0.718**	0.099	-0.055	**0.874**	0.109	0.076	-0.230
2. Changing the length of appointments to suit you		-0.100	**0.551**	0.212	-0.098	0.222	0.376	0.284
3. Offering appointments online or via apps		-0.164	**0.765**	-0.222	-0.002	**0.502**	0.009	-0.123
4. Changing how often you are asked to attend appointments		-0.062	**0.685**	-0.038	-0.145	**0.829**	0.063	-0.094
5. Give information to the clinician pre-appointment so that they can prepare		-0.159	**0.417**	0.378	-0.070	0.119	**0.671**	0.041
17. Provide support in relation to attending appointments (e.g. managing fears or uncertainties which might make attending difficult)		0.117	**0.511**	0.184	0.115	0.294	0.087	0.378
18. Appropriate distractions provided whilst waiting to be seen at appointment (e.g. tablet with headphones)		0.215	**0.680**	-0.162	0.292	**0.422**	0.001	0.039
1. Therapists who understand autism		0.047	-0.458	**0.970**	0.274	-0.044	0.282	0.202
6. Opportunity after the appointment to ask questions about conclusions		-0.244	-0.162	**0.838**	-0.137	0.010	**0.836**	-0.042
7. Appointments at an easily identified and accessible location		0.063	-0.057	**0.625**	0.026	-0.041	0.043	**0.668**
8. Appointments with an easily identified and familiar clinician		0.124	-0.110	**0.685**	-0.036	-0.253	0.026	**1.044**
13. Having a health summary document which can be shared with clinicians (e.g. hospital passport)		-0.019	0.168	**0.556**	0.143	-0.163	**0.861**	-0.188
14. A clinician who uses an approach which is informed by what you have said that you prefer (e.g. formal or informal)		0.035	0.016	**0.624**	0.066	-0.135	**0.736**	0.103
15. Identifying reasons that make it difficult to see a clinician or attend an appointment		0.096	0.193	**0.474**	0.053	0.230	**0.495**	0.041
16. Short waiting times to be seen when you attend appointments		0.268	0.169	0.295	0.002	**0.410**	-0.028	**0.421**

For peer review only

Key: SS = Sensory Environment, SC = Clinical and Service Context, CKC = Clinician Knowledge and Communication, F = Familiarity
Factor loadings that exceed the ≥ 0.4 loading criteria are denoted in bold

Supplementary Table 3: Inter-factors correlation matrices

	Mental Health			Physical Health			
	**F1**	**F2**	**F3**	**F1**	**F2**	**F3**	**F4**
**F1**	1.000			1.000			
**F2**	0.688	1.000		0.722	1.000		
**F3**	0.732	0.785	1.000	0.595	0.727	1.000	
**F4**				0.694	0.705	0.736	1.000

Supplementary Table 4: Model fit indices

Model fit statistic	Mental Health	Physical Health
**Root Mean Square Error of Approximation (RMSEA)**	0.032	0.032
**Non-Normed Fit Index (NNFI)**	0.994	0.995
**Comparative Fit Index (CFI)**	0.996	0.997
**Goodness of Fit Index (GFI)**	0.993	0.995
**Root Mean Square of Residuals (RMSR)**	0.0384	0.0361

The following cut-off values were used to determine acceptable goodness of fit:

RMSEA < 0.08
NNFI ≥ 0.95
CFI ≥ 0.90
GFI ≥ 0.95
RMSR < 0.08.

For peer review only

Supplementary Table 5: Response frequencies for the importance of adjustments in both samples

Adjustment	Mental Health Services Importance Response Frequencies (N)						Physical Health Services Importance Response Frequencies (N)					
	1 Low	2	3	4	5 High	M	1 Low	2	3	4	5 High	M
Clinicians who understand autism	1	0	8	57	468	3	2	4	8	64	322	7
Changing the length of appointments to suit you	19	40	102	181	194	1	12	20	52	135	177	11
Offering appointments online or via apps	89	66	128	120	120	14	65	45	84	73	121	19
Changing how often you are asked to attend appointments	32	33	173	163	130	6	51	42	110	109	69	26
Give information to the clinician pre-appointment so that they can prepare	11	17	34	147	322	6	20	13	43	130	190	11
Opportunity after the appointment to ask questions about conclusions	5	10	31	166	321	4	8	5	27	100	253	14
Appointments at an easily identified and accessible location	5	5	43	104	374	6	6	8	30	70	281	12
Appointments with an easily identified and familiar clinician	8	11	34	112	365	7	9	9	18	78	279	14
Change the sensory environment in the building that the appointment will take place in	32	28	83	178	209	7	23	23	56	123	166	16
Locations (e.g. waiting rooms) with small numbers of people	19	25	55	162	268	8	17	14	39	116	208	13
Locations with low noise levels	10	16	32	149	322	8	11	14	21	104	244	13
Locations with low light levels	43	42	103	153	185	11	20	27	81	121	138	20
Having a health summary document which can be shared with clinicians (e.g. hospital passport)	16	17	73	152	268	11	13	15	69	100	188	22
A clinician who uses an approach which is informed by what you have said that you prefer (e.g. formal or informal)	6	6	31	167	318	9	7	15	53	117	193	22
Identifying reasons that make it difficult to see a clinician or attend an appointment	13	8	70	174	260	12	10	8	48	114	202	25
Short waiting times to be seen when you attend appointments	7	20	44	168	293	5	7	14	34	111	227	14
Provide support in relation to attending appointments (e.g. managing fears or uncertainties which might make attending difficult)	20	21	58	146	282	10	18	14	50	109	198	18
Appropriate distractions provided whilst waiting to be seen at appointment (e.g. tablet with headphones)	62	66	142	121	132	14	51	31	101	93	106	25

Response option key: 1 = not at all important, 2 = not very important, 3 = neither important or unimportant, 4 = somewhat important, 5 = very important, M = missing response

For peer review only

Supplementary Table 6: Response frequencies for the availability of adjustments in both samples

Adjustment	Mental Health Services Availability Response Frequencies (N)							Physical Health Services Availability Response Frequencies (N)						
	1 Low	2	3	4	5 High	DK	M	1 Low	2	3	4	5 High	DK	M
Therapists who understand autism	81	105	32	27	14	261	17	86	84	30	23	10	146	28
Changing the length of appointments to suit you	102	52	29	32	10	292	20	96	68	25	27	16	134	41
Offering appointments online or via apps	94	32	25	26	18	321	21	76	41	35	36	45	125	49
Changing how often you are asked to attend appointments	50	46	32	37	16	335	21	65	34	21	19	8	192	68
Give information to the clinician pre-appointment so that they can prepare	73	50	31	42	23	301	17	92	56	25	29	21	139	45
Opportunity after the appointment to ask questions about conclusions	50	63	32	73	46	255	18	69	52	43	65	28	113	37
Appointments at an easily identified and accessible location	31	47	62	103	64	209	21	38	35	52	94	73	79	36
Appointments with an easily identified and familiar clinician	37	52	50	90	56	232	20	47	73	81	66	25	79	36
Change the sensory environment in the building that the appointment will take place in	108	63	26	27	13	279	21	114	55	16	14	9	124	45
Locations (e.g. waiting rooms) with small numbers of people	83	63	57	54	26	235	19	131	72	31	26	7	100	40
Locations with low noise levels	72	72	51	47	35	237	23	116	73	32	36	15	98	37
Locations with low light levels	101	60	31	36	19	261	29	129	76	12	23	6	111	50
Having a health summary document which can be shared with clinicians (e.g. hospital passport)	100	34	18	18	22	320	25	117	33	7	13	14	174	49
A clinician who uses an approach which is informed by what you have said that you prefer (e.g. formal or informal)	59	70	44	52	26	265	21	91	45	35	28	16	147	45
Identifying reasons that make it difficult to see a clinician or attend an appointment	72	55	29	37	19	300	25	92	52	23	20	11	152	57
Short waiting times to be seen when you attend appointments	78	72	43	69	25	228	22	102	97	50	29	8	79	42
Provide support in relation to attending appointments (e.g. managing fears or uncertainties which might make attending difficult)	95	51	32	35	22	280	22	100	67	24	12	15	136	43

For peer review only

Appropriate distractions provided whilst waiting to be seen at appointment (e.g. tablet with headphones)	145	36	14	21	13	281	27	138	38	10	9	13	137	62

Response option key: 1 = never available, 2 = rarely available, 3 = available about half the time, 4 = available most of the time, 5 = available all the time, DK = I don't know, M = missing response

For peer review only

Supplementary Table 7: McNemar tests showing a comparison of the adjustments that were most important to autistic people in mental and physical health services in a paired sample

Adjustment	N	Mental Health % very important	Physical Health % very important	P
Clinician who understands autism	217	87.6	80.2	.048
Changing the length of appointments to suit you	217	39.6	46.1	.211
Offering appointments online or via apps	206	23.8	28.6	.332
Changing how often you are asked to attend appointments	204	23.0	20.1	.526
Give information to the clinician pre-appointment so that they can prepare	214	64.5	49.5	.004
Opportunity after the appointment to ask questions about conclusions	214	62.6	64.0	.845
Appointments at an easily identified and accessible location	213	74.6	71.4	.520
Appointments with an easily identified and familiar clinician	211	69.7	72.0	.668
Change the sensory environment in the building that the appointment will take place in	210	40.0	44.8	.378
Locations (e.g. waiting rooms) with small numbers of people	213	50.2	51.2	.925
Locations with low noise levels	212	57.5	58.5	.924
Locations with low light levels	208	31.3	36.1	.373
Having a health summary document which can be shared with clinicians (e.g. hospital passport)	207	51.7	47.8	.497
A clinician who uses an approach which is informed by what you have said that you prefer (e.g. formal or informal)	206	64.6	46.6	**.001**
Identifying reasons that make it difficult to see a clinician or attend an appointment	202	47.0	51.5	.452
Short waiting times to be seen when you attend appointments	213	55.4	56.8	.848
Provide support in relation to attending appointments (e.g. managing fears or uncertainties which might make attending difficult)	208	55.8	50.0	.299
Appropriate distractions provided whilst waiting to be seen at appointment (e.g. tablet with headphones)	203	24.1	27.1	.572

* Significant P values following Simes adapted Bonferroni correction are highlighted in bold

For peer review only

Supplementary Table 8: McNemar tests showing a comparison of the adjustments that were least available to autistic people in mental and physical health services in a paired sample

Adjustment	N	Mental Health % never available	Physical Health % never available	P
Clinician who understands autism	65	29.2	40.0	.230
Changing the length of appointments to suit you	54	53.7	40.7	.296
Offering appointments online or via apps	43	46.5	34.9	.424
Changing how often you are asked to attend appointments	21	23.8	38.1	.549
Give information to the clinician pre-appointment so that they can prepare	54	35.2	38.9	.864
Opportunity after the appointment to ask questions about conclusions	72	20.8	25.0	.690
Appointments at an easily identified and accessible location	92	15.2	10.9	.503
Appointments with an easily identified and familiar clinician	82	14.6	17.1	.832
Change the sensory environment in the building that the appointment will take place in	51	60.8	51.0	.383
Locations (e.g. waiting rooms) with small numbers of people	75	32.0	49.3	.060
Locations with low noise levels	71	29.6	33.8	.711
Locations with low light levels	55	50.9	45.5	.701
Having a health summary document which can be shared with clinicians (e.g. hospital passport)	31	64.5	48.4	.302
A clinician who uses an approach which is informed by what you have said that you prefer (e.g. formal or informal)	58	29.5	31.0	.690
Identifying reasons that make it difficult to see a clinician or attend an appointment	43	48.8	46.5	1.000
Short waiting times to be seen when you attend appointments	91	29.7	36.3	.451
Provide support in relation to attending appointments (e.g. managing fears or uncertainties which might make attending difficult)	55	45.5	36.4	.442
Appropriate distractions provided whilst waiting to be seen at appointment (e.g. tablet with headphones)	51	74.5	72.5	1.000

For peer review only

STROBE Statement—Checklist of items that should be included in reports of ***cross-sectional studies***

	Item No	Recommendation	Page(s)	Line Number(s)
**Title and abstract**	1	(*a*) Indicate the study's design with a commonly used term in the title or the abstract	2	24
		(*b*) Provide in the abstract an informative and balanced summary of what was done and what was found	2-3	20-45
**Introduction**				
Background/rationale	2	Explain the scientific background and rationale for the investigation being reported	4-6	62-111
Objectives	3	State specific objectives, including any prespecified hypotheses	6	113-120
**Methods**				
Study design	4	Present key elements of study design early in the paper	6	124-126
Setting	5	Describe the setting, locations, and relevant dates, including periods of recruitment, exposure, follow-up, and data collection	6-8	128-175
Participants	6	(*a*) Give the eligibility criteria, and the sources and methods of selection of participants	6-7	129-162
Variables	7	Clearly define all outcomes, exposures, predictors, potential confounders, and effect modifiers. Give diagnostic criteria, if applicable	9-11	188-257
Data sources/ measurement	8*	For each variable of interest, give sources of data and details of methods of assessment (measurement). Describe comparability of assessment methods if there is more than one group	9-11	188-257
Bias	9	Describe any efforts to address potential sources of bias	10, 24	253, 490, 496
Study size	10	Explain how the study size was arrived at	8, 11	165-178, 238-239
Quantitative variables	11	Explain how quantitative variables were handled in the analyses. If applicable, describe which groupings were chosen and why	10, 11	213, 234-235, 243, 252-255
Statistical methods	12	(*a*) Describe all statistical methods, including those used to control for confounding	10-11	219-257
		(*b*) Describe any methods used to examine subgroups and interactions	10, 11	219-224, 249-257
		(*c*) Explain how missing data were addressed	11	235-238
		(*d*) If applicable, describe analytical methods taking account of sampling strategy	11	238-241
		(*e*) Describe any sensitivity analyses	N/A	N/A
**Results**				
Participants	13*	(a) Report numbers of individuals at each stage of study—eg numbers potentially eligible, examined for eligibility, confirmed eligible, included in the	7	146-150, 156-158, Table 2, Table 3

For peer review only

		study, completing follow-up, and analysed		
		(b) Give reasons for non-participation at each stage	7	146-150, 156-158
		(c) Consider use of a flow diagram	N/A	N/A
Descriptive data	14*	(a) Give characteristics of study participants (eg demographic, clinical, social) and information on exposures and potential confounders	13	Table 1
		(b) Indicate number of participants with missing data for each variable of interest	17, 19	Table 2, Table 3, Supplementary Tables 5&6
Outcome data	15*	Report numbers of outcome events or summary measures	N/A	N/A
Main results	16	(*a*) Give unadjusted estimates and, if applicable, confounder-adjusted estimates and their precision (eg, 95% confidence interval). Make clear which confounders were adjusted for and why they were included	21, Supplementary Info	403-455, Bonferroni Adjustments for McNemar (CIs N/A)
		(*b*) Report category boundaries when continuous variables were categorized	N/A	N/A
		(*c*) If relevant, consider translating estimates of relative risk into absolute risk for a meaningful time period	N/A	N/A
Other analyses	17	Report other analyses done—eg analyses of subgroups and interactions, and sensitivity analyses	12, 21	260-282, 400-408
**Discussion**				
Key results	18	Summarise key results with reference to study objectives	21-23	411-474
Limitations	19	Discuss limitations of the study, taking into account sources of potential bias or imprecision. Discuss both direction and magnitude of any potential bias	24	486-498
Interpretation	20	Give a cautious overall interpretation of results considering objectives, limitations, multiplicity of analyses, results from similar studies, and other relevant evidence	21-23	411-474
Generalisability	21	Discuss the generalisability (external validity) of the study results	21-22	419-427
**Other information**				
Funding	22	Give the source of funding and the role of the funders for the present study and, if applicable, for the original study on which the present article is based	27	565-571

*Give information separately for exposed and unexposed groups.

Note: An Explanation and Elaboration article discusses each checklist item and gives methodological background and published examples of transparent reporting. The STROBE checklist is best used in conjunction with this article (freely

For peer review only

For peer review only - http://bmjopen.bmj.com/site/about/guidelines.xhtml

available on the Web sites of PLoS Medicine at http://www.plosmedicine.org/, Annals of Internal Medicine at http://www.annals.org/, and Epidemiology at http://www.epidem.com/). Information on the STROBE Initiative is available at www.strobe-statement.org.

For peer review only
